# Deoxynivalenol Occurrence in Triticale Crops in Romania during the 2012–2014 Period with Extreme Weather Events

**DOI:** 10.3390/toxins13070456

**Published:** 2021-06-29

**Authors:** Valeria Gagiu, Elena Mateescu, Alina Alexandra Dobre, Irina Smeu, Mirela Elena Cucu, Oana Alexandra Oprea, Daniel Alexandru, Enuța Iorga, Nastasia Belc

**Affiliations:** 1 National Research & Development Institute for Food Bioresources—IBA Bucharest, 5 Baneasa Ancuta Street, 2nd District, 020323 Bucharest, Romania; alina.dobre@bioresurse.ro (A.A.D.); irina.smeu@bioresurse.ro (I.S.); mirela.cucu@bioresurse.ro (M.E.C.); enutai@yahoo.com (E.I.); nastasia.belc@bioresurse.ro (N.B.); 2 National Meteorological Administration (METEO—Romania), 97 Bucuresti-Ploiesti Street, 1st District, 013686 Bucharest, Romania; elena.mateescu@meteoromania.ro (E.M.); oprea@meteoromania.ro (O.A.O.); daniel.alexandru@meteoromania.ro (D.A.)

**Keywords:** deoxynivalenol, triticale, winter wheat, durum wheat, maize, rye, soil type, “Vb” cyclone, heavy precipitation, heavy flood, spatial and geographic distribution

## Abstract

This article aims to evaluate deoxynivalenol occurrence in triticale crops in Romania in years with extreme weather events (2012: Siberian anticyclone with cold waves and heavy snowfall; 2013 and 2014: “Vb” cyclones with heavy precipitation and floods in spring). The deoxynivalenol level in triticale samples (*N* = 236) was quantified by ELISA. In Romania, the extreme weather events favoured deoxynivalenol occurrence in triticale in Transylvania and the Southern Hilly Area (44–47° N, 22–25° E) with a humid/balanced-humid temperate continental climate, luvisols and high/very high risk of floods. Maximum deoxynivalenol contamination was lower in the other regions, although heavy precipitation in May–July 2014 was higher, with chernozems having higher aridity. Multivariate analysis of the factors influencing deoxynivalenol occurrence in triticale showed at least a significant correlation for all components of variation source (agricultural year, agricultural region, average of deoxynivalenol, average air temperature, cumulative precipitation, soil moisture reserve, aridity indices) (*p*-value < 0.05). The spatial and geographic distribution of deoxynivalenol in cereals in the countries affected by the 2012–2014 extreme weather events revealed a higher contamination in Central Europe compared to southeastern and eastern Europe. Deoxynivalenol occurrence in cereals was favoured by local and regional agroclimatic factors and was amplified by extreme weather events.

## 1. Introduction

Triticale (X *Triticosecale* Wittmack) is a cereal that combines the characteristics of wheat, i.e., superior biochemical properties and increased productivity, with those of rye, i.e., increased resistance to *Fusarium* spp. that produce mycotoxins deoxynivalenol–DON, zearalenone–ZEA, fumonisins–FUM, nivalenol–NIV, moniliformin–MON, T-2/HT-2 toxin and diacetoxyscirpenol–DAS. It is grown throughout the world in regions with unfavourable agroclimatic conditions (arid and semiarid areas, wetlands and acid soils), and is used for animal feed (pigs, poultry and ruminants such as cattle and sheep), human consumption (bread making, high-fibre extruded snacks, malting and brewing) and biofuel production [1,2,3]. Triticale is used in various proportions in animal feed formula, and can replace corn in the feeds for pig, chickens, cattle and sheep. The animal feed contains corn, barley, oats, wheat, rye, triticale and sorghum as the main cereals, minerals and mycotoxin binders [4].

According to statistical data from the Food and Agriculture Organization (FAO) for the 2012–2014 period, the average production share of triticale by region was Europe 93.5%, Asia 3.7%, Americas 1.4%, Oceania 1.3% and Africa 0.2%, and the top ten producers were Poland, Germany, France, Belarus, Russian Federation, China mainland, Hungary, Lithuania, Spain and Austria, with an average production of >353,924.67 tons [5]. Romania belonged to the second group of triticale producers, with an average production of ≤353,924.67 tons and yield of 2.8 tons/hectare in 2012, 3.4 t/ha in 2013 and 3.6 t/ha in 2014 [5]. The meteorological conditions caused massive contamination with mycotoxins in Europe, namely aflatoxins and ochratoxin A produced by *Aspergillus* spp. and *Penicillium* spp. in the extremely dry July–August 2012 and in 2013, with deoxynivalenol and fumonisin being produced by *Fusarium* spp. in the extremely rainy period of May–July 2014. These contaminations led to notifications and alerts in the Rapid Alert System for Food and Feed (RASFF), and to derogations from the European Commission’s maximum limits [6,7,8]. To prevent mycotoxins from entering the food and feed chains, universities and research institutes have developed national and international partnerships to determine the effect of climatic conditions in the field and postharvest contamination, and the European Commission and the National Sanitary Veterinary and Food Safety Authorities have developed regulations and a single integrated multiannual national control plan [7,9,10,11,12,13,14]. Research in meteorology and climatology has also been intensified to explain extreme weather events from a climate change perspective [15,16,17,18,19,20,21,22].

**Figure 1:**
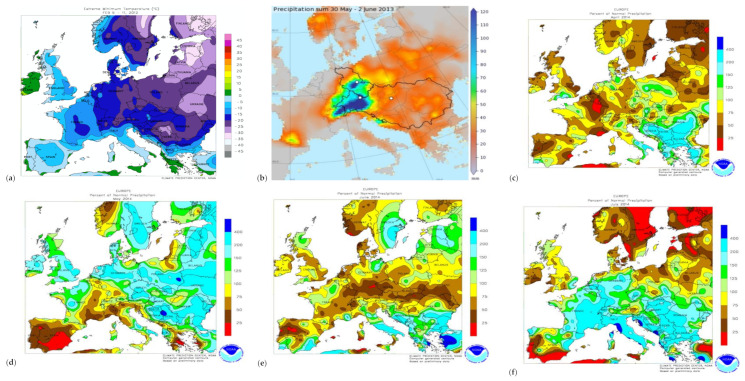
Spatial and geographic distribution of the extreme weather events in Europe in 2012–2014. (**a**) Extreme minimum temperature from 4 to 11 February 2012. (**b**) Extreme precipitation from 30 May to 2 June 2013. (**c**–**f**) Extreme precipitation in April, May, June and July 2014 [46,47] (GIS—Geographic Information System).

The years 2012, 2013 and 2014 are part of a 20 year trend in which the most significant positive temperature anomalies were registered. Globally, the positive land surface temperature anomalies in May–July of 2014, 2013 and 2012 year were in positions 10, 11 and 12 of the warmest years until 2020 (1880–2000 base period), and global mapping showed that the recorded anomalies in Europe had correspondence with conditions in western North America and northern Asia, which demonstrates the effect of large-scale atmospheric circulation [23]. In Europe, the positive land surface temperature anomalies were pronounced for May–July, the second warmest period being recorded in 2014 (May +2.23 °C, June +1.99 °C, July +1.97 °C) when considering the 1910–2000 base period [23]. In 2012–2014, Europe recorded extreme weather events that were synchronized with the North Atlantic Oscillation (NAO) and the Arctic Oscillation (AO) [17,18,20,22]. From 7 January to 17 February 2012, the whole of Europe was affected by boreal winter conditions produced by a Siberian anticyclone generated by the Arctic Oscillation. Extremely cold continental air from Russia brought ongoing frost to Eastern, Southeastern, Central and large parts of Western Europe, with heavy snowfall in Southeastern Europe including the Balkan Peninsula, Romania, Bulgaria, and Turkey (Figure 1a) [24,25,26,27]. From 20 to 29 May 2012, heavy precipitation and floods were recorded in Romania [28]. From 30 May to 2 June 2013, the North Atlantic Oscillation (the negative phase NAO^–^) generated the Mediterranean “Vb” cyclones named Dominik, Frederik and “Günther, which produced extreme precipitation and floods in Central Europe (Germany, the Czech Republic, Austria, Switzerland, Slovakia, Belarus, Poland, and Hungary) and Southeastern Europe (Serbia, Romania, and Bulgaria) (Figure 1b) [28,29,30,31,32,33,34]. From 11 May to 30 July 2014, the North Atlantic Oscillation (the negative phase NAO^–^) generated the Mediterranean “Vb” cyclone named Yvette, which caused heavy precipitation, storms and floods in Central Europe (Austria, Hungary, southeastern Germany, the Czech Republic, Slovakia, and southeastern Poland). The cyclone had a trajectory towards Ukraine, Belarus and European Russia (the Northwest and Central Federal Districts). It also generated the Mediterranean Vb(1c) cyclone named Tamara, which produced extreme precipitation and floods in Southeastern Europe (Croatia, Bosnia and Herzegovina, Serbia and Romania). This cyclone had a trajectory toward Crimea, Ukraine and European Russia, i.e., in the North Caucasus Federal District) (Figure 1c–f) [28,35,36,37,38]. The Mediterranean “Vb” cyclones from 2013 and 2014 caused heavy precipitation in Ukraine, Belarus, the Baltic States and European Russia (Figure 1b–f). In July and August of 2012 and 2013, the summer North Atlantic Oscillation (positive phase SNAO^+^) caused heat waves and severe drought in southeastern Europe (the Balkan Peninsula, Croatia, Serbia and Romania) [39,40,41].

The 2012–2014 weather events were classified as extreme weather events (cold waves, tropical cyclones, heat waves) because they had extreme values of meteorological importance, such as rare rates of occurrence in the 100-year return value, magnitude, temporal duration and timing, as well as spatial scale and multivariate dependencies, and caused loss of life and economic damages of millions of euros [16,23,41,42,43,44,45].

**Figure 2:**
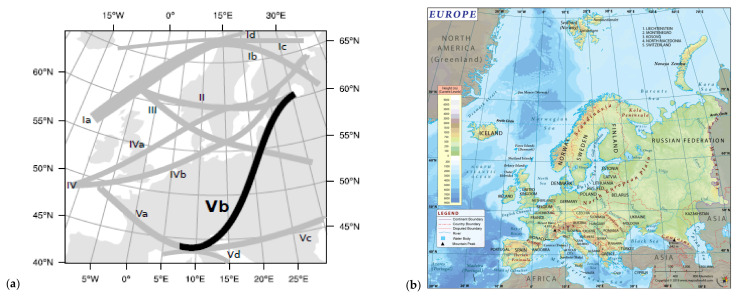
Map of cyclone tracks over Europe. (**a**) The divergence of track a “Vb” cyclone over the Ligurian Sea and northern Adriatic Sea [48]. (**b**) Map of Europe with countries and relief [51].

An in-depth analysis of extreme weather events in the countries with high levels of deoxynivalenol occurrence in cereals in 2012–2014 found that the Mediterranean “Vb” cyclones caused heavy precipitation and floods in June 2013 in Central Europe and May–July 2014 in central and southeastern Europe (Figure 2) [32,48,49]. A “Vb” cyclone is a large-scale air mass that forms in the western Mediterranean Sea (the Ligurian Sea in the Gulf of Genoa and the Adriatic Sea in the Gulf of Venice), and rotates around an intense centre of low atmospheric pressure, crosses northern Italy, leaves the Alps on the left side with a lower altitude (through Slovenia and Croatia), then propagates to the northwest (Central Europe: Austria, Hungary, the Czech Republic, and Slovakia) (Figure 2) [17,18,48,50]. “Vb” cyclones are rare events (2.3 per year and represent only 5% of all Central European cyclones) that can be traced first in the Atlantic Ocean critical region, and finally in northern Poland or even in European Russia (Figure 2) [18,48]. Their appearance seems to be synchronized with the North Atlantic Oscillation (NAO) and Arctic Oscillation (AO), both in the negative phase, that produce precipitation in the south [17,18,19,48].

The “Vb” cyclones have a standard route through Central Europe (43–47° N, 12–22° E: the Gulf of Genoa in the northern Adriatic Sea, and the Pannonian Plain in Poland) and three deviated routes (route 1a: Pannonian Plain in Romania, over the Western Carpathians and the Someș river basin, over the Eastern Carpathians in the Republic of Moldova–Ukraine; route 1b: The Pannonian Plain in Romania, through the Transylvania region, over the central part of the Eastern Carpathians in the Republic of Moldova, southern Ukraine and Crimea; route 1c: the Adriatic Sea near Bosnia and Herzegovina, Serbia, and Romania, through the Banat region, over the southern group of eastern Carpathians in the centre of the Republic of Moldova and the centre of Ukraine) [49]. Features of the Alps, Dinaric Alps and Carpathian Mountains (orography) influence the magnitude and spatial distribution of “Vb” cyclones and their effects (heavy precipitation and floods in central and southeast Europe) [18,48,49,50]. The activity area of the “Vb” cyclones has a transition climate resulting from the interaction of several types of cyclones (Atlantic, Mediterranean and “Vb”, generated by the ridges of the Alps, as well as polar and continental), with a predominance of Atlantic and Mediterranean cyclones in Central Europe, and a predominance of Mediterranean and continental cyclones in southeastern Europe [17,19]. The highest long-term total precipitation is produced by Atlantic and “Vb” cyclones, and the lowest is produced by continental and polar cyclones [19,48]. Floods in central and southeastern Europe occur between January and July, and depend on the amount of snowmelt in the Alps and Carpathians, the hydrological volume of rivers and streams, soil moisture saturation and heavy precipitation [52,53].

In Romania, the Atlantic and the “Vb” cyclones manifest in a weak form in the north of the West Plain and the northwest of Transylvania regions, as a result of the dam created by the Alps, Dinaric Alps and Carpathians Mountains [39,49]. Northwestern Romania is on the deviated routes 1a and 1b of the “Vb” cyclones, has a humidor balanced-humid temperate continental climate and represents an intersection location for the Atlantic air masses circulating through the Alps–Carpathian corridor (Germany, Austria, Slovakia, Hungary, Romania), with Mediterranean air masses circulating through the Pannonian corridor (Ligurian Sea, Northern Italy, Adriatic Sea, Dinaric Alps, Croatia, Bosnia and Herzegovina, Serbia, Hungary, Romania) and the Scandinavian air masses circulating through the Carpathian corridor (Poland, the Czech Republic, Slovakia, Ukraine, Hungary, Romania) [49,54]. In Romania, another precipitation area is located at the intersection of the Southern Hilly Area, the Southern Plain, and the Oltenia Plain regions [39,54,55].

Although triticale was developed for cultivation on moist, acid and arid soils, it is necessary to evaluate natural mycotoxin occurrence under the influence of typical weather conditions and extreme weather events.

Chernozem, phaeozem and luvisol are three of the 23 major soil types in Europe on which cereals are grown, and which differ in surface and depth horizons, textural differences in profiles, biological activity and geographic location [56,57]. Chernozems are soils with a significant accumulation of organic matter, a very dark brown or blackish surface horizon, CaCO_3_ deposits within 50 centimetres of the lower limit of the humus-rich horizon, basic pH, high porosity and good moisture-holding capacity. Chernozems cover 9% of Europe, especially in European Russia in the north Caucasus, southern and southeastern Ukraine (Eastern Europe), the Republic of Moldova, southeastern, southern and western Romania, and northern Serbia, Vojvodina (Southeastern Europe), as well as smaller areas in Hungary, Austria, the Czech Republic, Slovakia and Germany (Central Europe) [57]. Phaeozems are very similar to chernozems, with a closed surface horizon, rich in organic matter, without CaCO_3_ concentrations within 1 m and good moisture-holding capacity, but they are more intensively leached in wet seasons. Phaeozems cover 3% of Europe and are found in Central Europe, especially in Switzerland, Austria, Slovakia and Hungary [57]. Luvisols are characterised by lower organic matter content, a subsurface horizon of high activity clay accumulation and high base saturation and slightly acidic pH. Luvisols cover 6% of Europe and are found on well-drained landscapes on high subalpine and sub-Carpathian plateaus and sub-Balkans hills [56,57]. These agricultural soils characteristics are strongly influenced by the hydrological basins in which they are located, and by climate types [54,56,57,58]. In turn, soil characteristics influence the growth stages of cereals, which are earlier in arid regions of southern and southeastern Europe (with favourable conditions for *Aspergillus* spp. And *Penicillium* spp.) and delayed in central, eastern and northern Europe (with favourable condition for *Fusarium* spp.) [59,60,61,62,63].

During the 2012–2014 period with extreme weather events, natural deoxynivalenol occurrence in triticale was evaluated only in Romania (deoxynivalenol in 236 triticale samples of three successive crops) and Poland (26 mycotoxins in 20 samples of triticale, in 2014) (present study) [64]. Other European countries, including Italy, Switzerland, the Czech Republic, Slovakia, Hungary, Poland, Lithuania, Croatia, Bosnia and Herzegovina, Albania and Serbia, reported contamination with deoxynivalenol and other mycotoxins in maize, winter wheat, durum wheat, barley, soybean and spelt especially in extremely rainy 2014. Romania reported deoxynivalenol in winter wheat, durum wheat and rye crops [12,64,65,66,67,68,69,70,71,72,73,74,75,76,77,78,79,80,81,82,83]. The European and non-European countries that registered the deoxynivalenol-maize outbreak requested a derogation from the maximum allowed limit and received approval from the European Commission [6,66]. Since maize, wheat, triticale, rye, barley, oats and sorghum are components of animal feed, mycotoxin contamination in individual cereals was reflected in mixed animal feeds across Europe and Russia, with annual and regional differences [84,85,86].

Before 2012, natural deoxynivalenol occurrence in triticale was reported only among several kinds of cereal, namely maize, wheat, soybean, barley, bran, triticale, oat, rye and sunflower samples in Hungary in 1991–1998 [87], in bread wheat, durum wheat, triticale, rye, oat and barley samples in Poland in 2007 [88], in maize, wheat, barley, oat, soy, rye, sunflower, colza, rice and triticale samples from southeastern Romania in 2008–2010 [89] and twelve triticale genotypes in Romania [90]. Most publications present data on artificial infection and triticale breeding programs for resistance to *Fusarium* spp. [90,91,92,93,94,95,96].

This article aims to evaluate natural deoxynivalenol occurrence in triticale crops in Romania in the 2012–2014 period with extreme weather events. Romania’s data are used for knowledge transfer and to facilitate understanding of mycotoxin contamination in triticale and other cereals in some central, southeastern and eastern European countries affected by the extreme weather events in 2012–2014. These cereals are part of animal feeds and contribute to the cumulative contamination of mycotoxins transferred from feed to animals and then to humans.

The paper is the first article reporting natural contamination with deoxynivalenol in triticale crops and brings together deoxynivalenol occurrence in seven types of cereals under the influence of synergistic effects of some factors in fourteen European countries and three consecutive years. The article has a multidisciplinary approach (mycotoxin, climatology, agro-meteorology, agronomy, pedology, hydrology and geography) and contributes to raising awareness about the extreme weather events and their effects on deoxynivalenol occurrence in cereals and animal feed. Scientific data are useful for scientific research and legislative regulation, cereals producers and traders affected by climate change. The article is comprehensive due to the spatial and geographical distribution of deoxynivalenol in cereals in Europe during the 2012–2014 period with extreme weather events, its multidisciplinary approach and the need for stakeholders to understand and use it.

## 2. Results

### 2.1. Agrometeorologic Factors in Romania in the 2012–2014 Period with Extreme Weather Events

Through operational monitoring of extreme weather events in Romania during the 2012–2014 period, the National Meteorological Administration– NMA [97] issued short and medium-range forecasts (meteorological information, yellow weather alerts and orange weather warnings) and nowcasting warnings and alerts, using standard risk thresholds, according to the European meteorological norms and colour codes for each risk category. Most short and medium-range forecasts (*N* = 90) were issued in 2012 with three successive extreme weather events, and most nowcasting warnings and alerts (*N* = 2749) were issued in 2014, with extreme precipitation and floods in May–July (Figure S.1.1) [97]. Annual averages of air temperature and precipitation showed that 2012 was the coldest year due to the cold waves and heavy snowfall between January and February, the driest year due to the severe pedological drought of July to August (11.0 °C, 460 mm), and that the year 2014 was the wettest due to the extreme precipitation and floods in May to July (11.3 °C, 877 mm) (Figure S.1.2).

**Figure 3:**
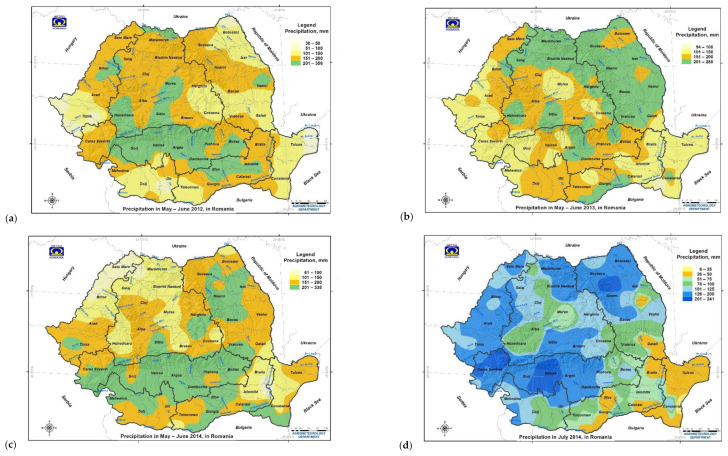
Spatial and geographic distribution of the cumulative precipitation in Romania in the 2012–2014 period with extreme weather events. (**a**–**c**) Cumulative precipitation in May–June in 2012, 2013 and 2014 (**d**) Cumulative precipitation in July 2014 (GIS—Geographic Information System).

In 2012, a severe winter with severe cold, frost and heavy snowfall in January–February occurred predominantly in Moldavia, the Southern Plain and Transylvania regions, which were more exposed to the Siberian anticyclone (Figure 1, Figure S.1.2 and S.1.3) [24,27]. In the spring, rising air temperatures and the heavy snow melt, coupled with heavy precipitation of 119.6 mm in May and cumulative heavy precipitation of 101–356 mm in May to June (Figure 3 and Figure S.1.2), led to increased soil moisture and river flow, followed by massive floods [28]. After the boreal winter and spring, with heavy precipitation and floods in May, Romania recorded a hot and dry summer (June–August: air temperature 23.8 °C, precipitation 39 mm, and accentuated heat stress) (Figure S.1.3), along with other countries from southeastern Europe (the Balkan Peninsula, Serbia and Croatia) [39,40,41]. In 2012, the NMA issued 90 short and medium-range forecasts (29 meteorological information, 41 yellow weather alerts and 20 orange weather warnings) and 1744 nowcasting warnings and alerts (Figure S.1.1) [97].

In 2013, the winter was mild, without cold waves and frost. In a moderate to normal spring, there was heavy precipitation at the end of May and the beginning of June (cumulative heavy precipitation of 101–285 mm in May to June) (Figure 3). Heavy precipitation increased river flows and caused massive floods in late May [28,46]. Besides, these regions were located at the eastern extremity of the “Vb” cyclones which produced large-scale heavy precipitation and floods in Central Europe [31]. Heat stress (air temperature ≥ 32 °C) was reduced or absent in most parts of the country. In 2013, the NMA issued 75 short and medium-range forecasts (26 meteorological information, 41 yellow weather alerts and eight orange weather warnings) and 2511 nowcasting warnings and alerts (Figure S.1.1) [97].

In 2014, the winter was mild, without cold waves and frost. In spring, a cumulative heavy precipitation of 101–338 mm was recorded in May to June in Transylvania, the Southern Hilly Area, the West Plain and northern Moldavia (Figure 3). Heavy precipitation continued in July 2014 (cumulative precipitation of 101–241 mm), which was considered the fourth wettest in the last 50 years in Romania, with torrential precipitation, storms, atmospheric electrical discharges and floods [28,98]. The extreme precipitation registered in July 2014 was between 101–241 mm in the west and centre of the Southern Hilly Area and the north of Moldavia, 51–125 mm in the Southern Plain, the centre of Transylvania and the south of Moldavia (Figure 3). The maximum cumulative precipitation were registered in the highest peaks of the Carpathian Mountains (orographic lifting precipitation), generating increase of river flow in the Southern Hilly Area and propagating floods in the Oltenia Plain and the Southern Plain. Therefore, the National Institute of Hydrology and Water Management issued red flood codes [98]. In 2014, the NMA issued 79 short and medium-range forecasts (28 meteorological information, 37 yellow weather alerts and 14 orange weather warnings) and 3740 nowcasting warnings and alerts [97]. Unlike in the previous years, the heat stress was moderate and reduced in June to August 2014, which delayed the ripening and harvesting of cereals. Low heat stress was recorded in the southern regions (Oltenia Plain, Southern Plain and Dobrogea) that are characterized by high historical aridity indices [55,99].

In Romania, the occurrence of Fusarium Head Blight disease and the production of deoxynivalenol mycotoxin in grains are favoured by agrometeorological factors during anthesis in May to June. From 1 September 2011 to 31 August 2014, the agrometeorological factors varied significantly amidst the agricultural regions. 

### 2.2. Deoxynivalenol Occurrence in the Triticale Crop in Romania in the 2012–2014 Period with Extreme Weather Events

A limit of DON ≥ 1000 µg/kg was used to evaluate the influence of extreme weather events and agroclimatic factors on natural deoxynivalenol occurrence in triticale crops in the 2012–2014 period with extreme weather events.

The use of a maximum limit of 1250 µg/kg, according to EC Regulation no. 1881/2006, would have omitted triticale samples with deoxynivalenol between 1000 µg/kg and 1250 µg/kg resulting from the soil leaching process or the reduction of the contamination level (the synergistic effect of the extreme precipitation and floods in May–July 2014, the soil types and the historical aridity indices on the “Vb(1c)” deviated route of cyclones in southern Romania and Southeastern Europe).

#### 2.2.1. Deoxynivalenol Occurrence in the Triticale Crop by Agricultural Year in Romania in 2012–2014

In the 2012–2014 period with extreme weather events, deoxynivalenol occurrence in triticale showed 83.1% positive samples (198/236) and ranged from <18.50 μg/kg to 3592.66 μg/kg, average 398.76 ± 651.89 μg/kg; the incidence of samples with DON ≥ 1000 μg/kg was 12.7% (30/236) (Table 1).

In 2012 and 2013, triticale had similar maximum contamination levels, although the weather events were very different in Romania. In 2012, deoxynivalenol occurrence in triticale recorded 85.5% positive samples, 4.3% samples with DON ≥ 1000 µg/kg, interval < 18.50–3378.40 µg/kg, with an average of 278.43 ± 575.30 µg/kg (Table 1).

**Figure 4:**
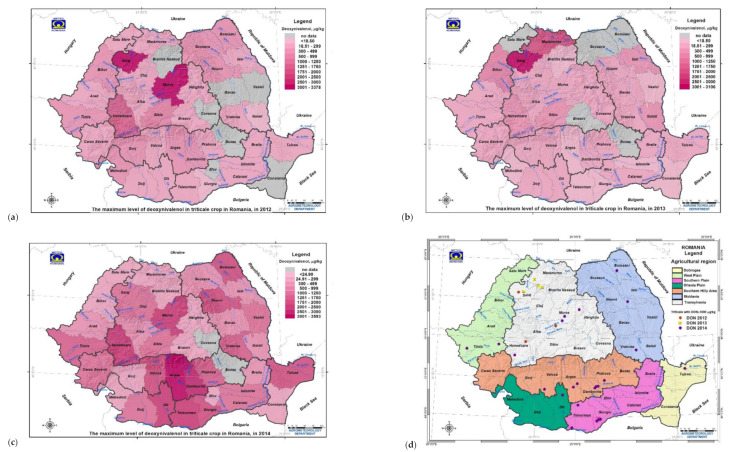
Spatial and geographic distribution of the maximum deoxynivalenol (DON) level in triticale crops in Romania in the 2012–2014 period with extreme weather events. (**a**–**c**) Maximum deoxynivalenol level in triticale crops by county and agricultural region in 2012, 2013 and 2014; (**d**) Origin of triticale samples with DON ≥ 1000 µg/kg in 2012–2014. River = blue colour; Danube River (Dunarea), in southern Romania (GIS—Geographic Information System).

In 2013, deoxynivalenol occurrence in triticale recorded 64.1% positive samples, 5.1% samples with DON ≥ 1000 µg/kg, interval < 18.50–3106.40 µg/kg, with an average of 205 ± 492.83 µg/kg (Table 1). Samples with DON ≥ 1000 μg/kg were detected only in Transylvania (45–47° N, 22.56–24.37° E; in the northwestern counties of Sălaj max. 3378.44 µg/kg in 2012 and 3106.44 µg/kg in 2013, Mureș max. 3170.89 µg/kg in 2012, and Maramureș max. 1961.94 µg/kg in 2013, and southwestern county of Hunedoara max. 1597.04 µg/kg) (Table 1 and Table S.2.1; Figure 4 and Figure S.2.1). In other agricultural regions with warmer climates and higher historical aridity indices, the maximum deoxynivalenol level did not exceed 500 µg/kg in 2012 and 2013, despite extreme weather events (Table 1 and Table S.2.1; Figure 1 and Figure 4 and Figure S.2.1).

In the extremely rainy 2014, deoxynivalenol occurrence in triticale was higher, with 96.7% positive samples, 25.6% samples with DON ≥ 1000 µg/kg, interval < 18.50–3592.70 µg/kg and average 661.90 ± 742.90 µg/kg (Table 1). In May–July 2014, Romania recorded heavy precipitation caused by the “Vb” and “Vb(1c)” cyclones in all agricultural regions of the country, the most affected being the Oltenia Plain, the Southern Plain, the Southern Hilly Area, northern Dobrogea and Moldavia. The extreme weather events determined an increase in deoxynivalenol occurrence in the triticale crop (96.7% positive samples, 25.6% samples DON ≥ 1000 µg/kg; interval < 18.50–3592.66 µg/kg). The maximum contamination varied between 1198.30–3592.70 µg/kg (Table 1 and Table S.2.1). The highest values of DON ≥ 1000 μg/kg were recorded in some counties in Transylvania (17.5%; max. 2399.60 µg/kg in Hunedoara, and max. 2165.68 μg/kg in Mureș) and the Southern Hilly Area (63.6%; max. 1006.77 μg/kg in Prahova, max. 2853.78 μg/kg in Dâmbovița, max. 3592.66 μg/kg in Argeș, and max. 1280.45 μg/kg in Vâlcea) (Table 1 and Table S.2.1; Figure 4 and Figure S.2.1). Unlike the previous two years, in 2014 there was high contamination in some counties in regions with warmer climates and higher aridity indices, but the number of samples and the level of maximum deoxynivalenol contamination were lower (West Plain—1198.34 μg/kg in Timiș; Oltenia Plain—1825.75 μg/kg in Olt; Moldavia—1533.76 μg/kg in Botoșani, 1301.69 μg/kg in Neamț, and 1041.67 μg/kg in Vrancea; Southern Plain—1924.29 μg/kg in Teleorman, and 1064.81–1250.76 μg/kg in Giurgiu; Dobrogea—1326.36 μg/kg in Tulcea) (Table 1 and Table S.2.1; Figure S.2.1).

**Table 1:** Deoxynivalenol (DON) occurrence in the triticale crop by agricultural region, geographic position, historical aridity indices (1900–2000) and agricultural year in Romania in the 2012–2014 period with extreme weather events.

Agricultural Region	Geographic Position		Aridity Indices, 1900–2000		Deoxynivalenol (DON) Occurrence in the Triticale Crop by Agricultural Region, Geographic Position, Historical Aridity Indices and Agricultural Year in Romania in the 2012–2014 Period with Extreme Weather Events															
					2012				2013				2014				2012–2014			
	Latitude, °N	Longitude, °E	Iar-dM, mm °C^−1^	CWD, mm	Analysed	Positive, %	≥1000 µg/kg, %	Interval Average ± SD, µg/kg	Analysed	Positive, %	≥1000 µg/kg, %	Interval Average ± SD, µg/kg	Analysed	Positive, %	≥1000 µg/kg, %	Interval Average ± SD, µg/kg	Analysed	Positive, %	≥1000 µg/kg, %	Interval Average ± SD, µg/kg
Dobrogea	44.6	28.5	20	−375	1	1 100	0 0	67.02	2	1 50	0 0	<18.50–19.43 18.97 ± 0.66	3	3 100	1 33	59.40–1326.40 486.74 ± 727.17	6	5 83.3	1 16.6	<18.50–1326.36 260.86 ± 522.54
Southern Plain	44.3	26.6	26	−258	13	11 84.6	0 0	<18.50–246.55 95.91 ± 75.24	11	5 45.5	0 0	<18.50–226.64 48.37 ± 61.08	21	21 100	5 23.8	24.37–1924.30 575.98 ± 510.96	45	37 82.2	5 11.1	<18.50–1924.29 308.32 ± 430.68
Moldavia	46.8	26.9	28	−194	9	6 66.7	0 0	<18.50–394.74 180.71 ± 173.48	13	11 84.6	0 0	<18.50–410.56 159.78 ± 140.45	13	13 100	3 23.1	151.75–1533.80 562.20 ± 449.54	35	30 85.7	3 8.6	<18.50–1533.76 314.63 ± 350.31
Oltenia Plain	44.4	23.7	37	−167	6	4 66.7	0 0	<18.50–482.75 124.46 ± 181.03	7	2 28.6	0 0	<18.50–54.06 27.73 ± 15.88	7	7 100	1 14.3	24.91–1825.80 610.03 ± 600.80	20	13 65	1 5	<18.50–1825.75 260.56 ± 439.90
West Plain	46.5	22.1	33	−150	8	7 87.5	0 0	<18.50–399.89 163.20 ± 140.47	7	6 85.7	0 0	<18.50–661.73 147.97 ± 231.12	11	9 82	2 18.2	<18.50–1198.30 359.75 ± 396.19	26	22 84.6	2 7.7	<18.50–1198.34 242.26 ± 302.81
Southern Hilly Area	45.1	24.7	39	−93	12	11 91.7	0 0	<18.50–498.08 224.05 ± 156.31	16	7 43.8	0 0	<18.50–245.55 40.84 ± 58.86	11	11 100	7 63.6	105.74–3592.70 1426.65 ± 1298.71	39	29 74.4	7 17.9	<18.50–3592.66 488.08 ± 901.96
Transylvania	46.4	24.3	46	−32	20	19 95	3 15	<18.50–3378.40 576.43 ± 1000.08	22	18 81.8	4 18.2	<18.50–3106.40 520.84 ± 840.07	23	23 100	4 17.4	26.57–2399.60 614.08 ± 719.10	65	60 92.3	11 16.9	<18.50–3378.44 570.94 ± 841.30
Romania	45.7	25.2	33	−181	69	59 85.5	3 4.3	<18.50–3378.40 278.40 ± 575.30	78	50 64.1	4 5.1	<18.50–3106.40 205 ± 492.83	89	87 96.7	23 25.6	<18.50–3592.70 661.90 ± 742.90	236	196 83.1	30 12.7	<18.50–3592.66 398.76 ± 651.89

#### 2.2.2. Deoxynivalenol Occurrence in the Triticale Crop by Geographic Position in Romania in 2012–2014

Transylvania (46.4° N, 24.3° E) was the agricultural region where the maximum deoxynivalenol level in triticale exceeded 1000 µg/kg each year (3378.40 µg/kg in 2012, 3106.40 µg/kg in 2013, and 2399.60 µg/kg in 2014). In the Southern Hilly Area (45.1° N, 24.7° E), triticale recorded DON ≥ 1000 µg/kg only in the extremely rainy 2014 (3592.70 µg/kg) (Table 1 and Table S.2.1; Figure 1 and Figure S.2.1).

In agricultural regions located at lower latitudes and higher longitudes (44.6–46.5° N, 22.1–28.5° E), triticale was contaminated with DON ≥ 1000 µg/kg only in the extremely rainy 2014 and had lower maximum values because these regions have higher historical aridity indices (West Plain max. 1198.34 µg/kg; Oltenia Plain max. 1825.75 µg/kg; Moldavia max. 1533.76 µg/kg; Southern Plain max. 1924.29 µg/kg; Dobrogea max. 1326.36 µg/kg) (Table 1 and Table S.2.1; Figure 4, Figures S.2.1, S.2.3 and S.3.2).

#### 2.2.3. Deoxynivalenol Occurrence in the Triticale Crop by Variety in Romania in 2012–2014

Of the 236 samples of triticale analyzed, 170 samples belonged to 21 certified varieties, and 66 samples had an unknown origin and were registered as “Other”. Regarding the resistance to *Fusarium* spp., according to the presentation forms of the triticale varieties by the producers, 6% were foreign varieties with very good resistance, 66% were Romanian varieties with good, medium or unreported resistance, and 28% “Other” varieties had no information (Table 2). The Haiduc, Titan, Stil and Gorun varieties were the most cultivated because they are autochthon varieties well adapted to Romania’s agroclimatic conditions. These triticale varieties have thick straw with prominent knots and an ear with pronounced thin hairs over a length of about 3–4 cm. They are recommended for acid soils with low fertility and are used in animal nutrition as concentrated feed, silage or green fodder [100].

In 2012 and 2013, triticale varieties had a low incidence of samples with DON ≥ 1000 µg/kg, and only in a few counties in the Transylvania region (in 2012 there were 3/68 samples: Trilstar 3378.44 µg/kg in Sălaj, Titan 3170.89 µg/kg in Mureș and 1597.04 µg/kg in Hunedoara; in 2013—4/78 samples: Titan 1534.42 µg/kg and 2053.30 µg/kg in Sălaj; Haiduc 3105.44 µg/kg in Sălaj, and Tremplin 1961.94 µg/kg in Maramureș) (Table 2, Tables S.2.1 and S.2.2). The Titan variety showed deoxynivalenol levels that were very different in Sălaj, Mureș and Hunedoara counties of the Transylvania region in 2012, 2013 and 2014, because the agroclimatic conditions of the counties are different (wet-balanced climate to humid climate) as a result of the geography of the Carpathian Mountains, which determine the different exposure to the air masses. The Sălaj, Maramureș and Mureș are neighbouring counties and part of northwestern Transylvania with the highest average annual precipitation, increased risk of floods caused by snow melting on the high peaks in the northeastern Carpathian Mountains and humid climate influences from Central Europe. At the national level, the incidence of deoxynivalenol-positive samples was higher in 2012 than in 2013 (85.3% and 65.4%, respectively), a possible explanation being water-saturated soils in spring as a result of snow melting and heavy precipitation in May 2012.

In 2014, with extreme precipitation and floods in the all regions of Romania, triticale had a higher incidence of samples with DON ≥ 1000 µg/kg (23/89) contamination being registered both in autochthon and foreign varieties (Haiduc 7/22, 24.91–2853.78 µg/kg; Titan 2/8, 42.25–2165.68 µg/kg; Stil 1/7, 136–2067.50 µg/kg; Gorun 1/6, <18.50–1866.29 µg/kg; Tulus 1/1, 1533.76 µg/kg; Cascador 1/1, 1353.65 µg/kg; Silver 1/1, 1198.34 µg/kg; ‘‘Other’’ variety 9/33, 24.37–3593.66 µg/kg) (Table 2, Tables S.2.1 and S.2.2). Moreover, triticale samples with DON ≥ 1000 µg/kg showed inadequate values for physico-chemical indicators and degrees of impurity (falling number, broken grains, sprouted grains, spoiled grains (data not shown)) as a possible result of torrential precipitation, storms, atmospheric electrical discharges and floods.

**Table 2:** Deoxynivalenol (DON) occurrence in the triticale crop by variety and agricultural year in Romania in the 2012–2014 period with extreme weather events.

Triticale Variety	Deoxynivalenol (DON) Occurrence in the Triticale Crop by Variety and Agricultural Year in Romania in the 2012–2014 Period with Extreme Weather Events.															
	2012				2013				2014				2012–2014			
	Analysed	Positive, %	≥1000 µg/kg, %	Interval Average ± SD, µg/kg	Analysed	Positive, %	≥1000 µg/kg, %	Interval Average ± SD, µg/kg	Analysed	Positive, %	≥1000 µg/kg, %	Interval Average ± SD µg/kg	Analysed	Positive, %	≥1000 µg/kg, %	Interval Average ± SD, µg/kg
Mungis	1	1 100	0 0	97.61	1	1 100	0 0	57.00	2	1 50	0 0	<18.50–104.47 61.49 ± 60.79	4	3 75	0 0	<18.50–104.47 69.40 ± 39.88
Odisej	-	-	-	-	3	0 0	0 0	<18.50	-	-	-	-	3	0 0	0 0	<18.50
Polego	1	1 100	0 0	174.25	2	2 100	0 0	<18.50–368.99 193.75 ± 247.83	3	3 100	0 0	51.19–106.41 78.98 ± 27.61	6	6 100	0 0	<18.50–368.99 133.11 ± 127.11
Amarillo	1	1 100	0 0	50.31	-	-	-	-	-	-	-	-	1	1 100	0 0	50.31
Gorun	-	-	-	-	4	2 50	0 0	<18.50–75.78 33.45 ± 28.24	6	5 83.3	1 100	<18.50–1866.29 560.23 ± 676.53	10	7 70	1 10	<18.50–1866.29 349.52 ± 573.18
Gorun 1	4	2 50	0 0	<18.50–245.50 82.45 ± 109.55	-	-	-	-	-	-	-	-	4	2 50	0 0	<18.50–245.50 82.45 ± 109.55
Haiduc	12	10 83.3	0 0	<18.50–482.75 151.39 ± 153.83	22	13 59.1	1 4.6	<18.50–3106.44 279.80 ± 651.61	22	22 100	7 31.8	24.91–2853.78 832.18 ± 738.03	56	45 80.4	8 14.3	<18.50–3106.44 469.29 ± 681.11
Trilstar	3	3 100	1 33.3	130.67–3378.44 1251.89 ± 1842.56	3	2 66.7	0 0	<18.50–77.16 47.48 ± 29.34	-	-	-	-	6	5 83.3	1 16.7	<18.50–3378.44 649.68 ± 1339.23
Trismart	-	-	-	-	-	-	-	-	3	3 *100*	0 *0*	26.57–552.53 *240.09 ± 276.58*	3	3 *100*	0 *0*	26.57–552.53 *240.09 ± 276.58*
Cascador	-	-	-	-	1	1 100	0 0	275.91	1	1 100	1 100	1353.65	2	2 100	1 50	275.91–1353.65 814.78 ± 762.08
Stil	5	5 100	0 0	48.18–778.60 267.91 ± 297.47	4	4 100	0 0	39.43–219.67 114.03 ± 89.28	7	6 85.7	1 14.3	136.33–2067.50 480.39 ± 704.38	16	15 93.8	1 6.3	39.43–2067.50 322.40 ± 497.86
Titan	19	16 84.2	2 10.5	<18.50–3170.89 405.95 ± 774.43	15	12 80	2 13.3	<18.50–2053.30 325.42 ± 613.63	8	8 100	2 25	42.25–2165.68 663.02 ± 690.14	42	36 85.7	6 14.3	<18.50–3170.89 426.15 ± 698.60
Tulus	-	-	-	-	-	-	-	-	1	1 100	1 100	1533.76	1	1 100	1 100	1533.76
Colina	-	-	-	-	1	1 100	0 0	47.80	-	-	-	-	1	1 100	0 0	47.80
Hercules	1	1 100	0 0	199.70	-	-	-	-	-	-	-	-	1	1 100	0 0	199.70
Plai	2	2 100	0 0	151.27–289.55 220.1 ± 97.78	1	1 100	0 0	26.14	1	1 100	0 0	201.35	4	4 100	0 0	26.14–289.55 167.08 ± 109.98
Silver	-	-	-	-	2	2 100	0 0	31.37–263.62 147.50 ± 164.23	2	2 100	1 50	329.70–1198.34 764.02 ± 614.22	4	4 100	1 25	31.37–1198.34 455.76 ± 511.32
Tarzan	-	-	-	-	-	-	-	-	1	1 100	0 0	663.16	1	1 100	0 0	663.16
Tremplin	1	1 100	0 0	231.32	2	2 100	1 50	21.91–1961.94 991.93 ± 1371.81	-	-	-	-	3	3 100	1 33.3	21.91–1961.94 738.39 ± 1064.79
Trialina	-	-	-	-	1	1 100	0 0	31.18	-	-	-	-	1	1 100	0 0	31.18
Trisidan	1	1 100	0 0	399.89	-	-	-	-	-	-	-	-	1	1 100	0 0	399.89
Other	17	14 82.4	0 0	<18.50–473.33 147.85 ± 139.12	16	7 43.8	0 0	<18.50–410.56 66.25 ± 108.96	33	33 100	9 27.3	24.37–3592.66 694.14 ± 866.67	66	54 81.8	9 13.6	<18.50–3592.66 392.93 ± 673.49
Romania	68	58 85.3	3 4.4	<18.50–3378.40 278.40 ± 575.30	78	50 65.4	4 5.1	<18.50–3106.40 204.98 ± 492.83	90	87 96.7	23 25.6	<18.50–3592.70 661.90 ± 742.90	236	196 83.1	30 12.7	<18.50–3592.66 398.76 ± 651.89

For the 2012–2014 period, the incidence of samples with DON ≥ 1000 µg/kg (30/236 samples) had corresponding behaviour of triticale varieties to *Fusarium* spp. in natural conditions with extreme weather events and the need to cultivate certified varieties (Haiduc, Titan, Stil and Gorun varieties) (Table 2, Tables S.2.1 and S.2.2). These triticale varieties showed similar average deoxynivalenol contamination in the periods 2010–2012 and 2012–2014, both with extreme weather events (Haiduc 555 µg/kg and 469.20 µg/kg, respectively; Titan 329 µg/kg and 426.15 µg/kg, respectively; Stil 248 µg/kg and 322.40 µg/kg, respectively; Gorun 271 µg/kg and 349.52 µg/kg, respectively; Cascador 508 µg/kg and 814.78 µg/kg in only two samples, respectively) (Table 2) [90].

#### 2.2.4. Deoxynivalenol Occurrence in the Triticale Crop and Other Cereals in Romania in 2012–2014

Compared to the other grains cultivated in Romania in 2012–2014, triticale showed intervals and incidence of DON ≥ 1000 µg/kg lower than in winter wheat (except for 2014), but higher than for rye and durum wheat (winter wheat 79/2504 samples, <18.50–5027.74 µg/kg; durum wheat 0/11, <18.50–483.99 µg/kg; triticale 30/236, <18.50–3592.66 µg/kg; and rye 1/75, <18.50–1217.70 µg/kg) (Table S.2.4; Figure S.2.3) [71,72,73].

Winter wheat showed deoxynivalenol contamination in almost all agricultural regions in 2012, with heavy precipitation in May (max. 1304.28–5027.74 µg/kg, except for the arid Dobrogea, max. 119.45 µg/kg) and in Moldavia, West Plain and Transylvania in 2013 with heavy precipitation in May–June (1878.34 µg/kg, 3602.56 µg/kg and 2125.02 µg/kg, respectively) (Table S.2.4; Figure S.2.3).

Durum wheat had deoxynivalenol contamination of less than 500 µg/kg in all three years, being cultivated in the areas with the highest historical aridity indices in the Dobrogea, Southern Plain, Moldavia, Oltenia Plain and West Plain (Table S.2.4; Figure S.2.3).

Rye showed deoxynivalenol contamination of less than 500 µg/kg in all regions in 2012 and 2013, and a maximum of 1217.70 µg/kg in Moldavia in extremely rainy 2014 (Table S.2.4; Figure S.2.3). 

In the extreme weather events of Spring 2012 and 2013, the triticale crops were contaminated with DON ≥ 1000 µg/kg only in Transylvania (3/69, max. 3378.40 µg/kg, and 4/78, max. 3106.44 µg/kg, respectively), but the contamination was higher in the interval and incidence of DON ≥ 1000 µg/kg in the conditions of extreme precipitation and floods from May–July 2014 (triticale 23/89, <18.50–3592.66 µg/kg; winter wheat 36/952, <18.50–3025.72 µg/kg; rye 1/30, <18.50–1217.70 µg/kg; durum wheat 0/3, 25.10–483.99 µg/kg). The highest values were recorded in the Southern Hilly Area, with a balanced-humid continental climate (max. 3592.66 µg/kg), and Transylvania, with a humid temperate continental climate (max. 2399.56 µg/kg), i.e., in the agricultural regions with the lowest historical aridity indices in Romania (Table S.2.4; Figure S.2.3). Triticale, a cereal resulted from the crossing of rye with wheat, had higher contamination than rye and less than winter wheat in 2012 and 2013, but the magnitude, duration and timing of extreme precipitation and floods and the cultivation in river meadows led to a higher deoxynivalenol level in the extremely rainy 2014 (Table S.2.4; Figure S.2.3).

#### 2.2.5. Deoxynivalenol Occurrence in the Triticale Crop by Hydrographic Basin in Romania in 2012–2014

Triticale samples contaminated with DON ≥ 1000 µg/kg were sampled from localities with heavy or extreme precipitation in hydrographic basins with flooding risk of mallow meadows (the Someș, Mureș, and Târnave rivers in Transylvania; the Timiș-Bega rivers in West Plain; the Prahova, Dâmbovița, Ialomița, Argeș, and Olt rivers in Southern Hilly Area; the Siret river and Moldavia tributary in Moldavia; the Olt, Vedea and Argeș rivers and the Danube River in the Southern Plain, the Danube Delta in Dobrogea) (Table S.2.2; Figure 3; Figure 4).

#### 2.2.6. Deoxynivalenol Occurrence in the Triticale Crop by Soil Type in Romania in 2012–2014

In Romania, extreme weather events in 2012, 2013 and 2014, caused the highest deoxynivalenol contamination in triticale grown on luvisol (<18.50–3592.66 µg/kg; 417.90 ± 733.75 µg/kg), followed by phaeozem (24.69–3170.89 µg/kg; 774.50 ± 966.58 µg/kg) and chernozem (<18.50–1924.29 µg/kg; 310.66 ± 405.93 µg/kg) (Table 3 and Table S.2.1; Figures S.2.2.b and S.3.4).

Luvisols favoured deoxynivalenol contamination in triticale crops in all the years: in 2012 with the boreal winter, and heavy precipitation and floods in May (92.3% positive; 7.7% samples with ≥1000 µg/kg; max. 3378.44 µg/kg); in 2013 with heavy precipitation in May–June (62.5% positive; 8.3% samples with ≥1000 µg/kg; max. 3106.44 µg/kg), and also in 2014 with extreme precipitation and floods in May–July (98.3% positive; 18.3% samples with ≥1000 µg/kg; max. 3592.66 µg/kg) (Table 3 and Table S.2.1; Figures S.2.2.b and S.3.4).

Triticales grown on phaeozems had higher deoxynivalenol contamination in 2012 (max. 3170.89 µg/kg) and in 2014 (max. 2165.68 µg/kg) compared to 2013 (max. 275.91 µg/kg) (Table 3 and Table S.2.1; Figures S.2.2.b and S.3.4). It is important to mention that all 15 samples of triticale grown on phaeozems are from the Mureș county in the Transylvania region.

Triticale grown on chernozems had low deoxynivalenol contamination in 2012 and 2013 (max. 367.48 µg/kg and max. 410.56 µg/kg, respectively), but they exceeded the maximum limit in 2014 (max. 1924.29 µg/kg) (Table 3 and Table S.2.1; Figures S.2.2.b and S.3.4).

**Table 3:** Deoxynivalenol (DON) contamination in the triticale crop by soil type and agricultural year in Romania in the 2012–2014 period with extreme weather events.

Soil Type (Scale 1:1,500,000)	Deoxynivalenol (DON) Contamination in the Triticale Crop by Soil Type and Agricultural Yearin Romania in the 2012–2014 Period with Extreme Weather Events. Interval; Average ± SD (Median); Positive Samples; Samples DON ≥ 1000 µg/kg			
	2012	2013	2014	2012–2014
Chernozem	<18.50–367.48 95.04 ± 95.13 (69.20) 22/29 (79.9%) 0/29 (0%)	<18.50–410.56 101.46 ± 127.53 (40.02) 14/24 (53.3%) 0/24 (0%)	<18.50–1924.29 599.73 ± 478.75 (426.60) 38/39 (97.4%) 10/39 (25.6%)	<18.50–1924.29 310.66 ± 405.93 (141.97) 74/92 (80.4%) 10/92 (10.9%)
Phaeozem (Luvic Pheozem)	170.75–3170.89 1373.41 ± 1586.05 (778.60) 3/3 (100%) 1/3 (33.3%)	24.69–275.91 177.93 ± 103.09 (213.11) 6/6 (100%) 0/6 (0%)	136.33–2165.68 1071.61 ± 922.45 (953.09) 6/6 (100%) 3/6 (50%)	24.69–3170.89 774.50 ± 966.58 (263.62) 15/15 (100%) 4/15 (26.7%)
Luvisol	<18.50–3378.44 333.33 ± 590.15 (169.68) 34/37 (91.9%) 2/37 (5.4%)	<18.50–3106.44 260.13 ± 616.57 (32.51) 30/48 (62.5%) 4/48 (8.3%)	<18.50–3592.66 661.13 ± 895.02 (228.34) 43/44 (97.7%) 10/44 (22.7%)	<18.50–3592.66 417.90 ± 733.75 (148.76) 107/129 (83%) 16/129 (12.4%)
Romania	<18.50–3378.40 278.40 ± 575.30 (124.73) 59/69 (85.5%) 3/69 (4.3%)	<18.50–3106.40 204.98 ± 492.83 (40.02) 50/78 (64.1%) 4/78 (5.1%)	<18.50–3592.70 661.90 ± 742.90 (329.70) 87/89 (96.7%) 23/89 (25.6%)	<18.50–3592.66 398.76 ± 651.89 (154.32) 196/236 (83.1%) 30/236 (12.7%)

### 2.3. Statistical Analysis

#### 2.3.1. Statistical Analysis of Agrometeorological Factors in Romania in 2012–2014

Although the extreme weather events affected the whole of Romania in 2012–2014, the classification of average air temperature and cumulative precipitation zones was influenced by geographic location, the humid temperate continental climate area and the disposition of the Carpathian Mountains, which create a dam and influences large-scale atmospheric circulation and agroclimatic aridity (Figure 1 and Figure 2) [39,49,54,55,56,58]. Therefore, the average annual air temperature had very significant differences by region and year (*p*-value < 0.001), and the cumulative precipitation had nonsignificant differences between the regions but very significant differences between the years (*p*-value < 0.001) (Tables S.3.1 and S.3.4).

2012 was the coldest (11.10 °C), with severe cold, frost and heavy snowfall in January to February (Table S.3.3; Figure 1 and Figure S.1.2.a). It recorded the highest amounts of average precipitation in May (119.6 mm) and heavy floods, followed by heat stress and severe drought during the summer, so that the cumulative precipitation per agricultural year was the lowest (461.38 mm) (Tables S.3.5 and S.3.6; Figure 3, Figures S.1.2.b and S.1.3). In 2012, Romania was under the influence of the boreal winter, with heavy precipitation and floods in May followed by heat stress and severe drought in June to August [24,25,26,27,28,97,99].

The year 2013 was the warmest (11.83 °C) and very significantly different from 2014 and 2012 (Table S.3.3; Figure S.1.2.a). It recorded the highest precipitation in June (102.9 mm), followed by a summer with lower heat stress and drought than in 2012 but higher than in 2014. The cumulative precipitation per agricultural year was 609.85 mm and significantly different from 2012 (Table S.3.6; Figure 1, Figure 3 and Figure S.1.2.b). In 2013, Romania was under the influence of the eastern front of “Vb” cyclones which produced extreme precipitation and floods in Central Europe (Figure 1 and Figure 3) [21,29,30,31,32,33,34].

The year 2014 had an average air temperature (11.48 °C) between the two years and significantly different from 2012 (Table S.3.3; Figure S.1.2.a). It had heavy precipitation in May, June and July (average 106.4 mm, 75 mm and 112.8 mm, respectively), followed by a summer with low or absent thermal stress. The cumulative precipitation per agricultural year was 705.96 mm and very significantly different from 2013 and 2012 (Table S.3.6; Figure 1, Figure 3 and Figure S.1.2.b).

The average air temperature showed very significant differences by agricultural region (*p*-value < 0.001), and the highest values were recorded in the regions with Mediterranean and Pontic climatic influences (Dobrogea, Southern Plain, and Oltenia Plain), followed by the regions with Atlantic and Mediterranean climatic influences (West Plain, and Southern Hilly Area) and those with Atlantic, Scandinavian–Baltic and continental climatic influences (Transylvania and Moldavia) (Tables S.3.1 and S.3.2). The highest and significantly different cumulative precipitation was recorded in the Southern Hilly Area (724.28 mm), followed by Oltenia Plain (616.41 mm), the regions most affected by the “Vb(1c)” cyclone in May–July 2014 (Tables S.3.5 and S.3.6; Figure 1, Figure 3 and Figure S.3.1). Heavy precipitation was also recorded in the West Plain, Transylvania, Southern Plain, and Dobrogea in May–July 2014 (Figure 1 and Figure 3). The southern West Plain, Oltenia Plain, Southern Hilly Area, Southern Plain, and Dobrogea regions are located at 42–45 °N, like the northern Adriatic Sea, southern Slovenia, Croatia, Bosnia and Herzegovina, and northern Serbia-Vojvodina, this being the route of the “Vb(1c)” cyclone in May–July 2014. In April to July 2014, Romania was affected by the “Vb” and “Vb(1c)” cyclones which produced extreme precipitation and floods in central and southeastern Europe [35,36,37,38,97].

Average annual air temperatures 11.10–11.83 °C (<15 °C), average cumulative precipitation 461.38–705.96 mm (>350 mm), weather conditions at anthesis in May–June (average air temperature 18.8 °C and precipitation 87 mm) influenced by the extreme weather events in 2012–2014, associated with a subhumid temperate continental climate and the geographic position, were factors that favoured the growth of *Fusarium* spp. and the production of deoxynivalenol (Tables S.3.2 and S.3.6; Figure S.1.2.a,b) [55,59,61].

#### 2.3.2. Statistical Analysis of Deoxynivalenol Occurrence in the Triticale Crop by Agricultural Year in Romania in 2012–2014

The highest average deoxynivalenol contamination was detected in triticale crops in the extremely rainy year 2014 (661.90 ± 742.90 µg/kg), followed by 2012 (278.40 ± 575.30 µg/kg) and 2013 (205 ± 492.83 µg/kg) (Table 1 and Table S.2.1; Figure S.2.2). The Kruskal-Wallis nonparametric test and mean rank for deoxynivalenol averages per year with the Chi-Square test, resulted in same order (2014, 2012, 2013) with very significant differences between the mean ranks (significance level = 0.000, *N* = 236) (Table S.3.8).

The average deoxynivalenol contamination was distinctly and significantly directly correlated with the soil moisture reserve in April (rxy = 0.211 **), distinctly and significantly inversely correlated with average air temperature in May (rxy = −0.294 **), distinctly and significantly directly correlated with average precipitation in May (rxy = 0.235 **) (Table S.3.9). The average deoxynivalenol contamination was distinctly and significantly directly correlated with cumulative precipitation (rxy = 0.331 **) and directly correlated with average soil moisture reserve (rxy = 0.153 *) (Table S.3.9). In 2014, the average deoxynivalenol contamination was robustly and very significantly directly correlated with average precipitation in April and May (rxy = 0.394 **, rxy = 0.318 **), and significantly correlated with average precipitation in June (rxy = 0.221 *), but there was no correlation with the average precipitation in July (rxy = 0.121) (Table S.3.10). These statistics proved that deoxynivalenol occurrence in the triticale crop was favoured by heavy precipitation and low average temperatures in May–June (anthesis) on water-saturated soils due to snow melting and precipitation in April (preanthesis), and the effect of heavy precipitation in July (postanthesis) was counteracted by high temperatures and historical aridity [101,102].

#### 2.3.3. Statistical Analysis of Deoxynivalenol Occurrence in the Triticale Crop by Geographic Position in Romania in 2012–2014

The average deoxynivalenol contamination in the triticale in Romania (43–48° N, 20–29° E) in 2012–2014 was distinctly and significantly directly correlated with historical aridity indices in 1900–2000, namely the de Martonne aridity index, Iar-dM and the climatic water deficit, CWD (rxy = 0.171 ** and rxy = 0.168 **, respectively) (Table S.3.9).

Transylvania (46.4° N, 24.3° E) and the Southern Hilly Area (45.1° N, 24.7° E) regions were classified as the first-class division of average deoxynivalenol contamination in all the years, in which deoxynivalenol occurrence was significantly inversely correlated with the average air temperature in May (rxy = −0.220 *), distinctly and significantly directly correlated with the average precipitation in May (rxy = 0.229 **) and distinctly and significantly directly correlated with cumulative precipitation (rxy = 0.333 **) (Tables S.3.7 and S.3.9). These regions recorded the highest deoxynivalenol level and have a humid temperate continental climate, luvisols and the lowest historical aridity indices (Table 1, Tables S.2.1 and S.2.2; Figure 4 and Figure S.3.2). In Transylvania, deoxynivalenol contamination was distinctly and significantly inversely correlated with average air temperature in May (rxy = −0.296 **) and significantly directly correlated with cumulative precipitation (rxy = 0.225 *) (Table S.3.9).

The West Plain, Oltenia Plain, Moldavia, Southern Plain and Dobrogea regions were classified as the second-class division of average deoxynivalenol contamination with nonsignificant differences between deoxynivalenol because the effect of heavy precipitation in July was counteracted by high historical aridity indices (Table 1, Tables S.2.1, S.2.2 and S.3.7; Figure S.3.2). This regional classification was maintained in 2014 with extreme precipitation and floods, but deoxynivalenol had an increased incidence and level even in the regions with high aridity indices (Table 1, Tables S.2.1, S.3.7 and S.3.8; Figure 4, Figures S.2.1, S.2.2.a and S.2.3).

As previously mentioned, Romania’s geographical positioning at 46° N, 25° E in Southeastern Europe, relief, hydrographic basins and large-scale atmospheric circulation are significant factors that condition climatic aridity indices (Iar-dM and CWD) in the agricultural regions of the country [39,49,54,55,56,58].

#### 2.3.4. Statistical Analysis of Deoxynivalenol Occurrence in the Triticale Crop by Variety in Romania in 2012–2014

Statistical analysis of triticale contamination with DON ≥ 1000 µg/kg (significance level = 0.834; *N* = 29) and DON ≤ 1000 µg/kg (significance level = 0.488; *N* = 207) in Romania showed that the distribution of deoxynivalenol was the same for all varieties in 2012–2014 (Figure S.3.3). One possible explanation may be that genotypes respond similarly to different temperature and precipitation regimes [60,64].

#### 2.3.5. Statistical Analysis of Deoxynivalenol Occurrence in the Triticale Crop by Soil Type in Romania in 2012–2014

The dependence of deoxynivalenol occurrence by soil type was analysed by the graphical method and the Kruskal-Wallis nonparametric test for independent samples, which revealed the same distribution of deoxynivalenol across soil types (significance level two-tails = 0.063; *N* = 236) (Table 3; Figures S.2.2.b and S.3.4). This distribution can be correlated with the fact that *F. graminearum* occurs on a wide range of soils and environmental conditions, and heavy and prolonged precipitation decreases soil pH by changing the water balance [103,104].

The luvisols in the northwest of Transylvania and the Southern Hilly Area were the most favourable for deoxynivalenol production, irrespective of pluviometric characteristics in the 2012–2014 period with extreme weather events (Figures S.2.2.b and S.3.4). Luvisols favour the growth of *Fusarium* spp. and deoxynivalenol level through acid pH and high humidity in plateaus and high hills, while chernozems favour bacterial populations and inhibit fungal growth by neutral to basic pH and low humidity in lowland regions [104,105]. Acid soils favour the saprophytic and virulent survival capacity of *Fusarium* spp. (FHB disease is inversely proportional to soil pH) but changing the soil pH to extremely acid or weak acid values decreases ascospore germination, and alternating periods with excessive humidity and severe drought changes microbiome structure [106,107]. Moreover, the soil pH is strongly influenced by geomorphological characteristics of the soil types, hydrological regime, precipitation and flooding, the edge of deciduous and softwood forests, crops residues and pollution [106]. In Europe, luvisols are found on plateaus and high hills of the Alps, Apennines Mountains, Carpathian Mountains, Balkan Mountains and Caucasus Mountains [56,57,108].

#### 2.3.6. Multivariate Tests of Between-Subjects Effects

Comparison of the averages of deoxynivalenol, air temperature, cumulative precipitation and soil moisture reserve, the physical context in which the contamination appears, as dependent variables, and the agricultural region and agricultural year as fixed factors, in a General Linear Model (GLM) Multivariate Analysis, was at the least significance (*p*-value < 0.05) for all components of variation (Table S.3.11).

## 3. Discussion

An in-depth analysis of deoxynivalenol occurrence in triticale in Romania and an extensive study of deoxynivalenol occurrence in cereals in Europe in 2012–2014 revealed mycotoxin occurrence in several types of cereals (triticale, winter wheat, durum wheat maize, soy, rye, barley, and spelt) that were affected by the same extreme weather events. Therefore, the scientific results for deoxynivalenol contamination in triticale crops in Romania could be extended to cereals cultivated in these countries and integrated at the European level to provide a large-scale picture of deoxynivalenol occurrence in cereals under the influence of the extreme weather events of 2012–2014 and a possible linkage to climate change. The integrated approach is advantageous because these cereals are major components of animal feed and are used in human consumption [1,2,3,4].

### 3.1. Extreme Weather Events in 2012–2014

In the 2012–2014 interval, severe weather events were recorded and classified as extreme weather events because they had a rare rate of occurrence in the 100-year return value, high magnitude, long temporal duration and timing, a continental spatial scale, multivariate dependencies, and produced damage with losses of both life and money [42,44,45].

The boreal winter of 2012 was attributed to the combined impacts of Arctic Ocean ice loss and the atmospheric circulation in the summer of 2011, as well as the movement of extremely cold air masses from Russia and heavy snowfall, which affected North Asia, the whole of Europe and North Africa (Figure 1) [24,25,26,27]. In Romania, the agricultural regions most affected by the boreal winter (frost, severe cold, heavy snowfall) were Moldavia, the eastern Southern Hilly Area and the Southern Plain, and Dobrogea, because they are most exposed to Siberian and continental air masses (Figure S.1.3.a). Despite the intercontinental scale of the boreal winter, deoxynivalenol occurrence in Europe and Romania was restricted because the abundant snow layer protected cereal crops and the summer had high heat stress and severe pedological drought (Figure S.1.3) [40,41]. Deoxynivalenol occurrence was reported in wheat in the Czech Republic, Switzerland and Lithuania, winter wheat and triticale in Romania, and durum wheat in northern Italy, but contamination is thought to be due to heavy precipitation in May–June on water-saturated soils because of snow melting (Table 1, Tables S.2.4 and S.3.9; Figure 3, Figure 4, Figures S.1.2, S.2.3 and S.4) [72,77,79,80,83,90].

The extreme floods in May–June 2013 in Central Europe were caused by heavy precipitation produced by the “Vb” cyclones after a cold and cloudy spring and were amplified by snow melting in the Alps and the Carpathian Mountains and by the water-saturation of soil, but no correlation with climate change was determined (Figure 1) [21,29,30,31,32,33,34]. The cyclonic front also affected southeast Europe (Serbia, Romania and Bulgaria), with heavy precipitation and floods (Figure 1 and Figure 3) [21,29,30,31,32,33,34]. The cyclone affected several regions of the European continent, but deoxynivalenol contamination was reported only in durum wheat in northern Italy, wheat, maize and cereals in the Czech Republic, maize in Switzerland, wheat in Hungary, winter wheat and triticale in northwestern Romania, and animal feed across Europe (Table 1, Tables S.2.1, S.2.4 and S.3.9; Figure 1, Figure 3, Figure 4, Figures S.1.2, S.2.1 and S.4) [71,74,77,79,80,83,84,85]. As in the previous year, the 2013 spring, with extreme precipitation and floods, was followed by summer with heat waves and severe drought (Figure S.1.3) [40,41].

In May–July 2014, extreme precipitation and floods were recorded caused by the “Vb” cyclone in Central Europe and the “Vb(1c)” cyclone in southeastern Europe (Figure 1, Figure 3, Figures S.1.2 and S.1.3) [35,36,37,38,109]. The meteorological and hydrological values of precipitation and floods were the highest in the last 100 years in Croatia, Bosnia and Herzegovina, and Serbia, and the highest in the last 50 years in Romania (Figure 1 and Figure 3) [35,36,37,38,98,109]. Analysis of climatic and meteorological factors that influenced the extreme floods in the Balkans showed that the “Vb(1c)” cyclone was stationary and produced extreme precipitation for several consecutive days, and that the floods were linked to planetary waves resonance [35]. In Romania, extreme precipitation and floods were recorded throughout the country, but the south was most affected, namely the Southern Hilly Area, the Oltenia Plain, the Southern Plain and Dobrogea (Figure 1 and Figure 3). Local precipitation during anthesis of cereals has been correlated with deoxynivalenol contamination in maize in northeastern Italy, wheat in Switzerland, wheat, grain and maize in the Czech Republic, wheat and maize in Hungary, wheat, triticale, winter barley and oats in Poland (Central Europe), unprocessed cereals and soybean in Croatia, wheat, barley, maize in Bosnia and Herzegovina, wheat in Albania, wheat and maize in Serbia, wheat, triticale and rye in Romania (southeastern Europe), wheat in Lithuania, and animal feed across Europe (Figure 1, Figure 4, Figures S.2.3 and S.4) [64,65,66,67,68,69,70,73,74,75,76,77,78,83,84,86]. From previous data, it was observed that the extreme weather events in 2012–2014 favoured the deoxynivalenol occurrence in all cereal types, but the extreme precipitation in July 2014 most affected maize crops (Figure S.4). Triticale in Romania and southeastern Poland was affected by precipitation in July 2014, but deoxynivalenol contamination was much lower than maize in other countries (present study) [64]. The highest frequency of heavy precipitation caused by the “Vb” cyclones was recorded in the Czech Republic, western Slovakia and southwestern Poland (7 out of 10 precipitation events) (Central Europe) [19], and this frequency can be correlated with the highest values of deoxynivalenol contamination in cereals in 2004–2018 (Figure S.4).

In the present study, the scientific data on deoxynivalenol occurrence in the triticale crops in Romania during 2012–2014 with extreme weather events were extended to other European countries affected by these events. Thus, deoxynivalenol occurrence in cereals under the influence of local and annual weather conditions was associated with large-scale weather events, geographic position, hydrographic basin and soil type.

### 3.2. Deoxynivalenol Occurrence in the Triticale Crop and Other Cereals by Agricultural Year

The highest incidences of triticale samples that were deoxynivalenol-positive and had DON ≥ 1000 µg/kg were recorded in 2014 with extreme precipitation and floods caused by the “Vb” and “Vb(1c)” cyclones (Figure 1, Figure 4 and Figure S.2.1; Table 1 and Table S.2.1), and in 2012 with the heavy snowfall in January–February and heavy precipitation and floods in May. In 2013, triticale with DON ≥ 1000 µg/kg was recorded only in northwestern Transylvania, which was under the influence of the eastern front of the “Vb” cyclones (Table 1 and Table S.2.1; Figure 1, Figure 4 and Figure S.2.1). In the 2012–2014 period with extreme weather events, only Romania and Poland reported natural contamination with deoxynivalenol in triticale, values being similar in the regions from northeastern Romania and southeastern Poland that have close historical agroclimatic conditions due to geographic position (present study) [64,90]. For the 2016–2018 period, Lithuania reported natural contamination with deoxynivalenol in triticale, especially during the harvest period with heavy precipitation, leading to increased mycotoxin levels [110].

The Siberian anticyclone in January–February 2012 did not directly influence deoxynivalenol contamination because all cereals and *Fusarium* spp. overwintered under a thick layer of snow (Figure S.4) [104]. In 2012, the contamination of durum wheat in northern Italy, winter wheat in Switzerland and wheat in the eastern Moravia of the Czech Republic, seemed to be determined by water-saturated soils due to snow melting and the local agroclimatic conditions (heavy precipitation and low air temperature at anthesis in May–June, acid soils, geographical position, and relief including plateaus, high hills and hydrographic basin) (Figure S.4) [72,77,79,80]. In Romania, the deoxynivalenol occurrence in triticale and winter wheat crops were produced by the same agroclimatic factors, to which there were added the extreme precipitation and floods in May (Table S.2.4; Figure S.4) [28,72].

In 2013, very high deoxynivalenol contamination in durum wheat in northern Italy, the high contamination of wheat in Switzerland, the very high contamination of wheat in eastern Moravia, of wheat in Hungary, and triticale and winter wheat in northwestern Romania, can be correlated with the “Vb” cyclones which produced extreme precipitation and floods in Central Europe [71,74,77,79,80].

In 2014, deoxynivalenol occurrence in cereals had a very high incidence and level, correlated with extreme precipitation and floods produced by the “Vb” cyclone in Central Europe and the “Vb(1c)” cyclone in southeastern Europe. Deoxynivalenol occurrence was reported in maize in northeastern Emilia Romagna in Italy (origin zone of the “Vb” cyclones), in cereals and soybean in Croatia, in winter wheat in the Bern canton of Switzerland, in cereals and maize in northern Bohemia of the Czech Republic, in triticale, wheat, winter barley and oats in the southeastern Polish regions of Borusowa, Małopolska (Central Europe), in wheat, maize and barley in Bosnia and Herzegovina, in maize in Albania, in wheat and maize in Vojvodina in northern Serbia, in triticale, winter wheat, durum wheat and rye in Romania (Southeast Europe), and in animal feeds in Russia, the Netherlands and globally [64,65,66,67,68,69,70,73,74,75,76,77,84,85,86].

### 3.3. Deoxynivalenol Occurrence in the Triticale Crop and Other Cereals by Geographic Position

In Romania, the Transylvania and the Southern Hilly Area regions were in the first-class division of average deoxynivalenol contamination in all the years (Table S.3.7) due to their geographic position (45–47° N, 22–25° E), acidic soil (luvisol, luvic phaeozem), humid and balanced-humid temperate continental climate given by low historical climate aridity (in 1900–2000: Iar-dM 39–46 mm °C^−1^; CWD −100–0 mm), and heavy to extreme precipitation during anthesis in May–June (Table 1, Tables S.2.1 and S.2.2; Figures S.2.1 and S.2.2). The West Plain, Oltenia Plain, Moldavia, Southern Plain and Dobrogea regions represented the second-class division of average deoxynivalenol contamination, with nonsignificant differences (Table S.3.7), because they have a semiarid and arid temperate continental climate given by the high climatic aridity indices (in 1900–2000: Iar-dM 20–33 mm °C^−1^; CWD −375–−100 mm), with chernozem soils in which the water balance is changed by prolonged heavy precipitation (Table 1, Tables S.2.1 and S.2.2; Figures S.2.1 and S.2.2).

A graphical analysis of the maximum and average deoxynivalenol contamination in the triticale crops in Romania showed a tendency for deoxynivalenol to increase at the geographical coordinates 45–47° N and 21–25° E, which correspond to the West Plain with a subhumid temperate continental climate, the western and central Southern Hilly Area with a balanced-humid temperate continental climate, and Transylvania with a humid temperate continental climate (Table 1 and Table S.2.1; Figure 4, Figures S.2.1 and S.2.2). The arable areas of these counties are located on plateaus and high hills in discontinuous areas of the Carpathian Mountains where luvisols are dominant and the climate is humid and determined by the interaction of cold intermontane air with the Atlantic and Scandinavian–Baltic air masses in the northwest, and with warm Mediterranean air masses in the southwest [39,49,54,55,56]. The West Plain and northwestern Transylvania are part of the standard and deviated (1a and 1b) Pannonian routes of the “Vb” cyclones. These regions are located at 44–47° N, 12–25° E and receive the most significant Atlantic influence on the western side of the western Carpathian Mountains, with the highest average annual precipitation and floods [17,49,53]. These Romanian regions and Central Europe (50.38° N, 14.97° E) have a similar climate, with strong Mediterranean influences in the southern part, and strong influences of Atlantic, Scandinavian, Baltic and Siberian continental climates in the northern part, where the influence of Alps, Dinaric Alps and the Carpathian Mountains are combined [17,19].

The maximum values of deoxynivalenol in cereals (winter wheat, durum wheat, maize, triticale, barley, rye, soy) were registered in 2014 on the trajectory of the “Vb” cyclone, which produced extreme precipitation and floods in Central Europe (Austria, Hungary, southeast Germany, the Czech Republic, Slovakia and southeast Poland; the cyclone had a trajectory towards northwestern Ukraine, Belarus, and European Russia in the northwest and the central Federal Districts) and the trajectory of the “Vb(1c)” cyclone which produced extreme precipitation and floods in southeastern Europe (Croatia, Bosnia and Herzegovina, Serbia and Romania; the cyclone had a trajectory towards Crimea, southern Ukraine and European Russia in the North Caucasus Federal District) (Figure 1c–f) [35,36,37,38].

Cereals and animal feed in Central Europe (45–52° N, 8–20° E) showed very high maximum values of deoxynivalenol contamination, especially between 49–52° N and 9–20° E (Eastern Moravia and northern Bohemia in the Czech Republic, Germany and southwestern Poland), compared to cereals in southeastern Europe (41–46° N, 15–25° E) and Eastern Europe (41–46° N, 15–25° E) (Figure S.4) [64,65,66,67,68,69,70,71,72,73,74,75,76,77,78,79,80,81,82,83,84,85,86,90,91,92,93,110,111,112,113]. The higher deoxynivalenol contamination in cereals in Central Europe (southern Germany, the Czech Republic, western Slovakia and southwestern Poland) is due to precipitation caused by the intersection of Atlantic air masses with the “Vb” cyclones and low historical agroclimatic characteristics that have been amplified by extreme weather events in 2012–2014 (Figure S.4) [19,54]. These climatic influences are also manifested in northwestern Romania (46–48° N, 23–24° E), favouring *Fusarium* spp. and deoxynivalenol production (Table S.2.1; Figure 4). Although Southern Europe (Emilia-Romagna in northern Italy) and southeastern Europe (Croatia, Bosnia and Herzegovina, Serbia and Romania) were under the influence of the “Vb(1c)” cyclone in May–July 2014, which produced the extreme floods in the last 100 and 50 years, respectively, deoxynivalenol occurrence in cereals was lower than in Central Europe due to the higher historical aridity (Figure S.4). In 2014, deoxynivalenol level was lower in lowland areas with heavy precipitation than in plateau and hill areas due to the alkaline soils, higher summer temperatures and historical climatic aridity that counteract the effect of heavy precipitation, or the heavy precipitation decreased the deoxynivalenol level from infected cereal ears (present study) [64,66].

The “Vb” and “Vb(1c)” cyclones caused heavy precipitation and floods over an area between 43–47° N and 12–22° E Europe, which covers most of Central Europe (45–52° N, 8–20° E; the standard route of cyclones) and southeastern Europe (41–47° N, 15–29° E; deviated routes of cyclones), but the eastern extremity of cyclonic fronts influenced eastern Europe (45–59° N, 30–40° E) (Figure 1 and Figure 2) [17,19]. Therefore, it can be considered that the spatial distribution of deoxynivalenol occurrence in cereals is correlated with the action area of the “Vb” cyclones. Furthermore, the geographic distribution of deoxynivalenol occurrence in cereals in 2012–2014, but also 2004–2018 (Figure S.4), corresponds to the epidemiological data on the geographical distribution of *F. graminearum* and *F. culmorum* in Europe in the period 2000–2013, which showed a dominance of 15-acetyl-deoxynivalenol chemotype trichothecenes at the latitude of 54.4 ± 10.8 °N, in strong correlation with the climatic conditions [59]. Eastern Europe, the Baltic States and northern Europe have lower average temperatures and precipitation, being under the influence of the Arctic climate, which stops the progression of Fusarium Head Blight disease and causes deoxynivalenol contamination to be sporadic [61,63,112,113]. In Romania, the region of Moldavia (46° N, 26.76° E) receives Scandinavian and Baltic and continental climatic influences, and the cold and dry climate is not favourable for the infection of cereals with *Fusarium* spp. and the production of deoxynivalenol (Table 1 and Table S.2.1). However, heavy precipitation produced by the “Vb” cyclones led to deoxynivalenol occurrence in several types of cereals in 2014 (Table 1, Tables S.2.1 and S.2.4; Figure 4, Figures S.2.1, S.2.2, S.2.3 and S.2.4) [71,72,73,90].

### 3.4. Deoxynivalenol Occurrence in the Triticale Crop by Variety

The triticales that showed deoxynivalenol contamination in 2012–2014 were represented by some certified varieties with no reported medium or good resistance to *Fusarium* spp. (Haiduc, Titan, Stil, Gorun, Trilstar, Tremplin, Tulus, and Cascador) and unidentified varieties (“Other”) (Table 2 and Table S.2.3). The certified Haiduc, Titan, Stil and Gorun varieties showed a medium deoxynivalenol contamination that was similar in the periods 2012–2014 and 2010–2012, both with extreme weather events (present study) [90]. The average deoxynivalenol contamination in triticales was similar in some varieties grown in five consecutive years with extreme weather events of different duration, intensity and timing, which demonstrates the importance and effectiveness of breeding programs for Fusarium head blight (FHB) resistance and deoxynivalenol contamination, and acclimatization programs to regional agroclimatic conditions [90,94,95].

The statistical distribution of deoxynivalenol contamination was the same for all triticale varieties in 2012–2014, but there were differences between agricultural regions with the same weather events and different values of the historical climatic aridity (Table 2 and Table S.2.3; Figure S.3.3). Triticale varieties in Romania showed much lower natural and artificial contamination than those in southern Germany and Poland (Central Europe with a humid climate), but also Lithuania (Baltic States with a cold and dry climate) because Romania has a drier climate that diminishes or counteracts the effect of heavy precipitation, especially in the warmer southern regions (Figure S.4) [64,71,72,73,90,91,92,93,96,110].

### 3.5. Deoxynivalenol Occurrence in the Triticale Crop and Other Cereals by Type

Compared to the other cereals cultivated in Romania in 2012–2014, triticale showed intervals and incidence of DON ≥ 1000 µg/kg lower than in winter wheat, except in 2014 with extreme precipitation and floods, but higher than durum wheat and rye (Table S.2.4; Figure S.2.3) [71,72,73]. In 2012 and 2013, triticale had lower maximum deoxynivalenol contaminations than in winter wheat, but in 2014 it showed higher maximum contamination due to extreme precipitation and floods, cultivation area in river meadows, and the thick straw with prominent knots.

In 2012–2014, winter wheat in Romania was less contaminated than wheat in Central Europe (Switzerland in the Bern canton, Slovakia, the Czech Republic in eastern Moravia and northern Bohemia, and Poland), but more contaminated than in other countries in Southeastern Europe (Bosnia and Herzegovina, Albania, and Serbia) and the Baltic States (Lithuania) (Figure S.4) [64,65,70,75,77,78,80,81,82,83,90].

In Romania, durum wheat is grown in very arid areas in Dobrogea, the Southern Plain, Moldavia, Oltenia Plain and the West Plain, and has shown deoxynivalenol contamination of less than 500 µg/kg in all the three years [71,72,73]. Contamination in 2012 and 2013 was minimal compared to that recorded in northern Italy with a humid climate with alpine influences, but similar to southern Italy with an arid Mediterranean climate (Figure S.4) [79].

Maize was the most affected by extreme precipitation and floods in May to June 2013 and May to July 2014. Like wheat and triticale, maize was the most contaminated in northern Italy, Central Europe (the Czech Republic in eastern Moravia) and Southeastern Europe (Albania, Croatia, Bosnia and Herzegovina, and northern Serbia in Vojvodina) (Figure S.4) [65,66,67,68,69,70,75,76]. In 2014, due to a very high level of deoxynivalenol and other mycotoxins in maize, these countries requested and received derogation from the European Commission [13,66].

Triticale is used in small proportions in animal feed formulas, but maize and wheat are major components. In 2012–2014, the increased level and incidence of mycotoxins in cereals were found in animal feed, with annual and regional differences. The highest feed contamination was recorded in central and northwestern Europe in 2014 and 2013, whereas southeastern and eastern Europe recorded lower values [84,85,86,114]. The massive contamination and negative experience of the RASFF notification of aflatoxin B in maize and animal feed, and aflatoxin M1 in cow’s milk, in Europe in 2013 (due to the severe pedological drought in Southeastern Europe in Croatia, Serbia, and Romania in July to August 2012) obliged the National Sanitary Veterinary and Food Safety Authorities to carry out rigorous controls of mycotoxins in animal feed and to prevent obtaining contaminated animal feed over the maximum allowed limits [8,14,85,115]. The control of cereals, raw materials and finished animal feed is mandatory because contamination level and toxicological effect of mycotoxins accumulate in animals, being transferred to humans through milk, meat and eggs. Moreover, when purchasing cereals, the interregional variation of mycotoxins must be considered because statistically significant differences of deoxynivalenol in pig urine from regions with different climatic conditions were determined [116]. The humid transitional climate in Central Europe causes an increased and constant incidence of deoxynivalenol contamination in cereals and animal feed, while the temperate continental climate in southeastern Europe reduces the incidence and level of deoxynivalenol contamination and favours aflatoxins and ochratoxin A contamination [8,13,14,84,114,117].

### 3.6. Deoxynivalenol Occurrence in the Triticale Crop and Other Cereals by Hydrographic Basin

Triticale with DON ≥ 1000 µg/kg was sampled from crops cultivated in the meadows of Someș and Mureș rivers in Transylvania, the Timiș–Bega rivers in the West Plain, the Olt, Argeș, Teleorman and Ialomița rivers in the Southern Hilly Area, the Vedea river in Oltenia Plain, the Olt, Vedea and Câlniște rivers in the Southern Plain and the Siret river in Moldavia (Table S.2.2; Figure 4d). The rivers in Transylvania and the West Plain are tributaries of the Tisza river, affluent of the Danube River, and the other rivers are affluents of the Danube River (Figure 4) [118]. These rivers spring from the Romanian part of the Carpathian Mountains, and their hydrographic basins, collect water from tributaries, precipitation or melting snow. The Danube River is Europe’s second-longest river after the Volga; it springs in Germany, crosses Central and Southeastern Europe and drains into the Black Sea, forming the Danube basin. The river basins in Transylvania and the West Plain regions present a high and very high flooding risk, along with regions from some Central European countries (Germany, Switzerland, Austria, the Czech Republic and Poland), the main causes of floods being prolonged heavy precipitation, snow melting, very wet soils, impermeable rock and deforestation [53].

In 2014, the maximum deoxynivalenol contamination in triticale in Romania was higher in upstream than downstream in the Argeș-Dâmbovița-Prahova hydrographic basin (Southern Hilly Area counties of Argeș max. 3592.66 µg/kg and max. 3264.41 µg/kg, and Dâmbovița max. 2853.78 µg/kg, versus the Southern Plain counties of Teleorman max. 1924.29 µg/kg and Giurgiu max. 1064.81–1250.76 µg/kg) (Table S.2.2; Figure 4c,d). Usually, the agroclimatic conditions in the extreme south of Romania, including Teleorman, Giurgiu, Olt and Dolj counties, are not favourable for the contamination of cereals with toxins produced by *Fusarium* spp. because the average annual temperatures exceed 24 °C, the region has a very high risk of pedological drought (the climatic water deficit is high, CWD −250–−200 mm) and soils have a coarse/very coarse sand texture and basic pH; conditions favourable for *Aspergillus* spp. and *Penicillium* spp. occurrence (Table 1 and Table S.2.1; Figure 4) [12,55,58,89].

From the geographic analysis of the European countries that presented deoxynivalenol contamination in cereals in 2012–2014, it was observed that they have hydrographic basins that were affected by extreme precipitation and floods during the analyzed period and had an increased risk of floods in 1987–2002 (Figure 1 and Figure S.4) [53]. Melting of the heavy snow in January to February, heavy precipitation in late May 2012, the heavy precipitation in May to June 2013 and the heavy precipitation in May to July 2014 led to heavy floods in these hydrographic basins in central and southeastern Europe (Figure 1) [27,28,29,30,31,32,33,34,35,36,37,38,98]. Moreover, amplification and prolongation of the heavy precipitation in July 2014 were associated with hail, storms, atmospheric electrical discharges with loss of life and damage amounting to millions of euros [35,36,109,119].

The hydrographic basins include luvisols in plateaus and high hills (upstream), phaeozems and chernozems (downstream), but alluvial deposits have led to the emergence of fluvisols, which are soils with slightly acid pH in the river meadows [57]. In Europe, fluvisols have the widest spread near the Rhine, Elbe, Vistula, Po, Danube and Volga Rivers [120,121]. Prolonged extreme precipitation causes an increase of the rivers’ flow and floods meadows, alters the soil water regime, lowers the soil pH, and favours structurally and functionally fungal populations. In these conditions, phyla *Ascomycota*, and class *Sordariomycetes* (including *F. graminearum* and *F. culmorum*) are dominant [122,123,124,125,126,127]. Moreover, the alternation of periods with excessive moisture and drought severely affects the soil microbiome [107].

### 3.7. Deoxynivalenol Occurrence in the Triticale Crop and Other Cereals by Soil Type

In Romania, extreme weather events in 2012–2014 determined the highest deoxynivalenol contamination in triticale grown on luvisol, followed by phaeozem (luvic phaeozem) and chernozem (Table 3 and Table S.2.1; Figure S.2.2.b). Deoxynivalenol occurrence in triticale on luvisols was detected every year, with the highest incidence of positive samples and ≥1000 µg/kg (Table 3 and Table S.2.1; Figure S.2.2.b). The average maximum values of deoxynivalenol in triticale were 1.75 times higher for luvisol (acid soil) compared to chernozem (neutral and slightly alkaline soil). The soils from Mureș county are classified as phaeozems, but local analysis showed they are luvic phaeozem. This subclassification and analysis on a scale of less than 1:1,500,000 may explain the maximum contamination between triticales grown on luvic phaeozems and luvisols (max. 3170.89 µg/kg and max. 3592.66 µg/kg, respectively) (Table 3) [57]. The very low deoxynivalenol contamination on chernozems in 2012 and 2013 can be attributed to their physico-chemical properties (basic pH, CaCO_3_ in the deep horizon, high porosity and good moisture-holding capacity) [57]. However, the abundant and prolonged precipitation in May–July 2014 favoured high contamination in triticale on arid chernozems in the northeastern Oltenia Plain (max. 1825.80 µg/kg), Southern Plain (max. 1924.30 µg/kg), Moldavia (max. 1533.80 µg/kg), and Dobrogea (max. 1326.40 µg/kg) (Table 1 and Table S.2.1; Figures S.2.1, S.2.2.b, S.2.4 and S.3.2), which were affected by the “Vb(1c)” cyclone.

In 2012–2014, luvisols and luvic phaeozems in plateaus and high hills favoured the highest incidence and level of deoxynivalenol in cereals (durum wheat, winter wheat, maize, triticale, unprocessed cereals) in northern Italy in northwestern Emilia-Romagna, in Central Europe in the Czech Republic, eastern Moravia and northern Bohemia, Slovakia, Hungary, and Poland in Borusowa in the Małopolska region, and in Southeastern Europe in Romania in Transylvania and the Southern Hilly Area regions, Serbia, Croatia, and Bosnia and Herzegovina [57,64,75,76,77,79,80,81,82,90,128,129,130,131,132,133]. The geographic distribution of *Fusarium* spp. in Europe in 2000–2013 supports this geographic distribution of deoxynivalenol occurrence in cereals in Europe in 2012–2014, which suggests that plateaus and high hills are favourable areas, such as the northern Ardennes Plateau in France and Belgium, the Bavarian, Renan and Bohemian Plateaus in Germany, the Małopolska and Lublin Plateaus in Poland, the Podolian Plateau in Ukraine and the Transylvanian and Moldavian Plateaus in Romania [59,108]. In 2014, the increased incidence and level of deoxynivalenol in cereals (winter wheat, triticale and maize) cultivated on chernozems in southern Romania in the Southern Plain and northern Serbia in Vojvodina (the main agricultural regions located at the same latitude of 43–44° N and having similar agroclimatic conditions in the two neighbouring countries) can be explained by the changing water balance because of prolonged heavy precipitation and floods on the principal affluents of the Danube River, followed by a leaching process which consists of water runoff through the soil with the removal of Ca^2+^ and relatively immobile Al^3+^ accumulation, as well as the sudden transition of soil pH from basic to acidic [12,35,36,37,38,67,68,70,73,103,109]. Deoxynivalenol occurrence in cereals in Romania and Serbia in the extremely rainy May to July of 2014 was significantly higher compared to the extremely rainy May to June of 2013 and the extremely dry summer of 2015 [12,67,68,73]. This aspect is essential because the alternation of periods of excessive moisture and drought severely affect the soil microbiome, and *Fusarium* spp. can survive on residues for more than two years, especially after maize cultivation [106,107]. Studies on the identification of *Fusarium* spp. in winter wheat, durum wheat and triticale in Romania in 2005–2009 and 2012–2013, showed that the *F. graminearum* 15-acetyl-DON chemotype had the highest prevalence of 75–85% and the *F. culmorum* 3-acetyl-DON chemotype had the highest aggressiveness. Other species identified were *F. proliferatum*, *F. moniliforme*, *F. anthophilum*, *F. subglutinans*, *F. poae*, *F. oxysporum* and *F.* *verticillioides* [134,135]. *F. graminearum* and *F. culmorum* were correlated with precipitation in some counties of Transylvania in Mureș and Brașov, the Southern Hilly Area in Argeș and the southern West Plain in Timiș, which are on the route of “Vb” and “Vb(1c)” cyclones and have luvisols in the area of plateaus and high hills. *F. poae*, *F. oxysporum* and *F. verticillioides* were found in the Oltenia Plain in Dolj and the Southern Plain in Călărași (arid climate, chernozem) [134,135]. It is possible that the high incidence of these *Fusarium* species in 2012–2013 was due to water-saturated soils due to snow melting and to the extreme precipitation and floods in May 2012, as well as to the extreme precipitation and floods in June 2013. Data correlate with the high incidence and level of deoxynivalenol in the triticale samples analyzed in the present study (Table 1, Tables S.2.1 and S.2.4) [134,135]. A new manuscript on wheat infestation with *Fusarium* spp. and deoxynivalenol under the influence of the agroclimatic conditions in Romania (2015–2016) is being developed. 

The dominant occurrence of *Fusarium* spp. and deoxynivalenol in cereals grown on acid soils and in animal feed in regions with humid temperate climate was found both in Europe and globally (present study) [59,61,84,85,86,103,136]. In Europe, the contamination of triticale and other cereals with *Fusarium* spp. and deoxynivalenol decreases from west to east due to the reduction of precipitation brought by Atlantic and Mediterranean cyclones and decreasing air temperature due to continental and polar cyclones, and from north to south due to increasing air temperature and soil temperature, decreasing precipitation and increasing soil alkalinity (increasing climate aridity) [19,48,59,61,84,85,86,103,136,137,138,139,140,141,142,143].

## 4. Conclusions

The period 2012–2014 was characterised by positive temperature anomalies globally, which led to extreme weather events represented by cold waves and heavy snowfall, extreme precipitation and floods in spring, and high thermal stress and severe pedological drought in summer. In 2012, the boreal winter was produced by a Siberian anticyclone generated by the Arctic Oscillation and correlated with climate change, and heavy precipitation was local on water-saturated soils in spring. The extreme precipitation and floods in 2013 and 2014 were produced by the “Vb” cyclones generated by the North Atlantic Oscillation (the negative phase NAO^–^) and planetary wave resonance, and they were not correlated with climate change. The “Vb” cyclones crossed Central Europe by the standard Pannonian route or Southeastern Europe 1b and 1c deviated routes, and produced extreme precipitation and floods, which favoured *Fusarium* spp. attack and deoxynivalenol occurrence in cereals. Heavy precipitation before and during cereal anthesis favoured deoxynivalenol occurrence, and the heavy precipitation delayed harvesting.

In Romania, extreme weather events favoured deoxynivalenol occurrence in triticale crops in Transylvania and the Southern Hilly Area, which are located between 44–47° N, 22–25° E, have a humid temperate continental climate due to Atlantic and Mediterranean influences, acid soils (luvisol, luvic phaeozem) and a rich hydrological network with a high and very high risk of floods. In Moldavia, the Southern Plain, Dobrogea, the Oltenia Plain and the West Plain, maximum deoxynivalenol contamination was lower, although the amounts of heavy precipitation in May–July 2014 were higher; however, chernozems have higher historical aridity indices. In these regions, deoxynivalenol contamination is sporadic and caused by prolonged heavy precipitation that changes the water balance of chernozems (alkaline soil), lowers pH and changes the soil microbiota (favours the fungal population and dominance of *Fusarium* spp.). Triticale varieties reacted similarly in the same weather and agroclimatic conditions and had similar average contamination in two periods with extreme weather events in 2009–2014, similar to triticale contamination in southeastern Poland in 2014. Compared to other cereals cultivated in Romania, triticale was less contaminated than winter wheat but more contaminated than durum wheat and rye in the extremely rainy 2014. The most significant effect on the deoxynivalenol contamination in triticale was the weather conditions before and during the anthesis (soil moisture reserve in April, average air temperature and precipitation in May and June), while the influence of postanthesis precipitation (July) was counteracted by higher summer temperatures and historical aridity indices, or the prolonged heavy precipitation decreased deoxynivalenol level from infected cereals ears. Multivariate analysis of the factors influencing deoxynivalenol occurrence in triticale showed at least a significant correlation for all components of variation source (average of deoxynivalenol, agricultural year, agricultural region, average air temperature, cumulative precipitation, soil moisture reserve and historical aridity indices) (*p*-value < 0.05).

In 2012–2014, deoxynivalenol occurrence in triticale was reported only in Romania and southeastern Poland under the influence of heavy precipitation in May–July 2014, but the most affected cereals in Europe were maize and wheat in 2014, especially when maize was the precursor crop. These cereals are major components in animal feed manufacturing, whose cumulative toxicological effect can be transmitted to humans.

The analysis of the spatial and geographical distribution, incidence and level of deoxynivalenol in cereals in the countries affected by the extreme weather events in 2012–2014 revealed higher contamination in the countries of Central Europe compared to southeastern and eastern Europe. The highest deoxynivalenol contamination in 2004–2018 was detected in cereals cultivated in countries located at 49–51° N, 15–20° E (southern Germany, the Czech Republic, western Slovakia, southeastern Poland), at geographical coordinates close to those from northwestern Romania (46–48° N, 23–24° E). These regions are located in plateaus and high hilly areas, on the standard route of the “Vb” cyclones, have strong Atlantic and Mediterranean climate influences, acid luvisol and luvic phaeozem soils, rich hydrological basins such as the Rhine, Elbe, Oder, Vistula, and Danube Rivers with a high and very high risk of floods, and alluvial soils with acid pH in meadows (fluvisols). Although “Vb” cyclones form in northern Italy (the Ligurian Sea and the Adriatic Sea), deoxynivalenol occurrence in cereals was lower than in Central Europe and southeastern Europe due to the direction of movement of warm air masses, the dam created by the Alps, Dinaric Alps and Carpathians Mountains that amplify precipitation (orographic lifting precipitation). The “Vb” cyclones in 2013 and 2014 also affected Eastern Europe (Lithuania and European Russia) and the heavy precipitation favoured cereal contamination that was found in the feed contamination over the maximum limit.

In summary, deoxynivalenol occurrence in cereals in Romania and Europe was favoured by local and regional factors (geographic position 46–51° N, 15–24° E; humid temperate climate, acidic soils in plateaus and high hills or rivers meadow, low historical aridity indices) and amplified by extreme weather events such as heavy precipitation and floods which had extreme values of meteorological importance.

The research adds a new and expanded understanding of the meteorological and agroclimatic factors that influence cereal contamination with *Fusarium* spp. and deoxynivalenol, and opens up new research opportunities in the context of present and future climate change. Identification and quantification of *Fusarium* spp., their mycotoxins in cereals and the correlation with regional agroclimatic factors, must be addressed in a standardised way to analyse large-scale weather influences. In this way, researchers, regulators, cereals producers and traders can have a comprehensive picture of the mycotoxin occurrence in cereals and establish appropriate measures to the challenges of climate change.

## 5. Materials and Methods

Research methods are presented in full to demonstrate the in-field and postharvest replicability of the scientific approach [12].

### 5.1. Triticale Sampling

Triticale crops were sampled (*N* = 236) by inspectors of the Ministry of Agriculture and Rural Development (MARD) in the frame of the Grain Quality Evaluation Programmes in 2012–2014 (Orders of the Minister no. 67646 from 23 May 2012, no. 77176 from 28 March 2013, and no. 109718 from 17 April 2014).

*Sampling procedure*. In each county, the areas cultivated with triticale were divided into a variable number of areas each of 3000 ha, depending on the pedoclimatic particularities, as well as the practical possibilities of organizing the sampling activity. For each 3000 ha, 10 to 15 plots representative from the point of view of the state of the culture, the applied technology, the size and the form of land ownership; the grains harvested on these surfaces represented a triticale lot. The establishment of these plots was done 2–3 weeks before harvesting, directly in the field by persons designated for the sampling and was highlighted in a table containing all necessary identification data (area harvested, triticale variety, owner). From each plot thus determined, an amount of up to 1 kg of triticale representing the elemental sample was taken directly from the combine. The composite sample representing the quantity of seeds of the same variety, consisted in bringing together and homogenizing all elemental samples taken from a lot. The number of composite samples was determined by dividing the area cultivated with each variety at 3000 ha and corresponding to the total area cultivated in each county and the weight occupied by each variety. The elementary samples harvested in the same area and for the same variety were pooled in preset places, homogenized and reduced to 6 kg each, constituting the composite sample. Each composite sample was packed in a canvas bag, labelled inside and outside, stating the variety, species and county where the composite sample was made. Thus formed, the composite samples were sent to the laboratory by each agricultural direction of MARD. The composite samples were sent with a map of the county on which the sampling areas and the localities from where they were formed were delimited. Given that the determination of grain quality indices and the interpretation of the results was done by crop and varieties, it was forbidden to mix the harvested samples between the areas and make mixtures of varieties. The sampling procedure was representative for the entire areas harvested with triticale in Romania, namely 48,019 ha in 2012, 72,529 ha in 2013, and 76,713 ha in 2014, and ensured the grading process of triticale crops.

In Poland, wheat, triticale, barley and oats were grown on experimental plots of 36 m^2^ each (with soil fertilization and fungicides) in five regions with different agroclimatic conditions [64]. Poland is the largest triticale producer globally, with areas harvested of 991,797 ha in 2012, 1,176,700 ha in 2013 and 1,306,025 ha in 2014 [5].

### 5.2. Mycotoxin Analysis

The samples were milled with a Retsch ZM 200 ultracentrifugal mill (Retsch, Haan, Germany) and analyzed for deoxynivalenol (DON) by the enzyme-linked immunosorbent assay (ELISA) coupled with a Sunrise microplate reader (absorbance 450 nm; Tecan, Salzburg, Austria). Mycotoxin analysis was performed with Ridascreen^®^ DON test kit, with a limit of detection of 18.50 µg/kg (R-Biopharm, Darmstadt, Germany). The maximum levels and guidance levels for mycotoxins in products intended for animal feed are established by European Commission Regulations [115].

The National Research & Development Institute for Food Bioresources– IBA Bucharest is accredited for the analysis of mycotoxins in cereal and food samples according to SR EN ISO/IEC 17025:2018 (Certificate No. LI 1210 from 19 November 2020; accreditation by the Romanian Accreditation Association (RENAR)) and participated in the FAPAS Proficiency Testing Programme (FERA, Sand Hutton, NY, UK) with satisfactory results, demonstrating laboratory performance and results comparable to laboratories worldwide that use different methods.

### 5.3. Geographic Coordinates

The northern latitude and eastern longitude (degrees) of each Romanian county (*N* = 41) were determined using Google Earth [144], and were grouped by agricultural regions (*N* = 7; Transylvania, Southern Hilly Area, Moldavia, Oltenia Plain, West Plain, the Southern Plain, and Dobrogea) based on their agroclimatic factors (Figure 4d; Table S.2.1).

European countries (*N* = 14) were located using Google Earth [144] and grouped by regions and the “Vb” cyclone routes: northern Italy as the origin of the cyclones; Central Europe (Switzerland, Germany, the Czech Republic, Slovakia, Hungary, and Poland) as the standard route of the cyclones; Southeastern Europe (Croatia, Bosnia and Herzegovina, Albania, Serbia, and Romania) as the deviated route of the cyclones; and Eastern Europe (Lithuania, and European Russia) as the eastern extremity of the standard and deviated routes of the cyclones (Figure S.4).

### 5.4. Agroclimatic Data

An agricultural year was defined to start on 1st September and end on 31st August of the subsequent calendar year. Agrometeorological factors (air temperature, °C; precipitation, mm; soil moisture reserve, mc/ha) were recorded by the official agrometeorological network of Meteorological Weather Stations (MWS) (*N* = 159 stations, from which 127 automated stations and 32 classical meteorological stations) using Ceres-Wheat and Decision Support System for Agrotechnology Transfer (DSSAT) v.3.5. software, from 1 September 2011 to 31 August 2014. The network of automatic stations belonging to the National Meteorological Administration is connected to the International Meteorological Telecommunication System, ensuring the connectivity and operative transfers of primary data and processing across the Meteorological Network.

The dominant agricultural soil types (chernozem, phaeozem and luvisol) in each county were set at a scale of 1:1,500,000 according to the Soil Atlas of Europe [57], which ensured statistical analysis at the regional level in Romania, as well as analysis and traceability at the European level. The soil types and pH were verified with official maps, scientific publications in pedology and other maps published online, but not mentioned because the number of references would have been higher [56,57,58,63,93,121,128,129,130,131,132,133,142]. In Romania, data on soil fertilization, pH correctors and other agricultural practices are not known because the triticale grading was done for sale.

The aridity indices (de Martonne aridity index—Iar-dM, mm °C^–1^; climatic water deficit—CWD, mm) of each county were estimated for cultivated areas based on data published for the 1900–2000 period [55] to determine the correlation with deoxynivalenol on a long-term basis.

### 5.5. Data Processing and Statistical Analysis

All data were collected in an Excel file with the following variables: Years (2012, 2013 and 2014, with extreme meteorological events), deoxynivalenol (DON), agricultural region, county, geographic coordinates (northern latitude, eastern longitude), and agroclimatic data (agrometeorological factors including air temperature, precipitation and soil moisture reserve; soil types of chernozem, phaeozem and luvisol and the aridity indices Iar-dM and CWD).

The influences of the geographic position and agroclimatic conditions on in field triticale contamination with deoxynivalenol in Romania were determined through statistical analysis using SPSS v.23 software (IBM, Armonk, NY, USA) (Statistical Package for the Social Sciences software with ANOVA, general linear model, Pearson correlation, statistical tests for the comparison of means, nonparametric Kruskal–Wallis and Jonckheere–Terpstra tests, graphical methods, ultivmariate tests of between-subjects effects) and class divisions [145]. Probability was considered to be statistically significant at *p* ≤ 0.05.

### 5.6. Spatial and Geographic Distribution

The spatial and geographic distribution of average precipitation values and maximum deoxynivalenol values in triticale crops in Romania in the 2012–2014 period with extreme meteorological events was achieved through the Geographic Information System (GIS) technology, using the Open Source ArcMap program version 10.1 (The Environmental Systems Research Institute (ESRI), Redlands, California, the United States) [146].

## Data Availability

Not applicable.

## Supplementary Materials

The following are available online at https://www.mdpi.com/article/10.3390/toxins13070456/s1, **S.1**: Extreme weather events in Romania during the 2012–2014 period (Figures S.1.1–S.1.3). **S.2**: Deoxynivalenol (DON) in the triticale crop in Romania during the 2012–2014 period with extreme weather events (Tables S.2.1–S.2.4; Figures S.2.1–S.2.3). **S.3**: Statistical analysis of deoxynivalenol (DON) in the triticale crop in Romania during the 2012–2014 period with extreme weather events (Tables S.3.1–S.3.11; Figures S.3.1–S.3.4). **S.4**: Deoxynivalenol occurrence in the cereal crops in Europe on the route of the ‘’Vb’’ cyclones (Figure S.4).
