# Supplementary material for: Deoxynivalenol Occurrence in Triticale Crops in Romania during the 2012–2014 Period with Extreme Weather Events
Source: Toxins (Basel). 2021 Jun 29;13(7):456. doi: 10.3390/toxins13070456 (PMC8310060; doi:10.3390/toxins13070456)
Supplement: Supplementary file 1 [file toxins-13-00456-s001.zip › toxins-1216305-supplementary.pdf]

toxins

MDPI

Supplementary Materials: Deoxynivalenol Occurrence in Triticale Crops in Romania during the 2012–2014 Period with Extreme Weather Events

Valeria Gagiu, Elena Mateescu, Alina Alexandra Dobre, Irina Smeu, Mirela Elena Cucu, Oana Alexandra Oprea, Daniel Alexandru, Enuța Iorga and Nastasia Belc

S.1: Extreme weather events in Romania during the 2012–2014 period

Note: An agricultural year is defined to start on 1st September and end on 31st August of the subsequent calendar year.

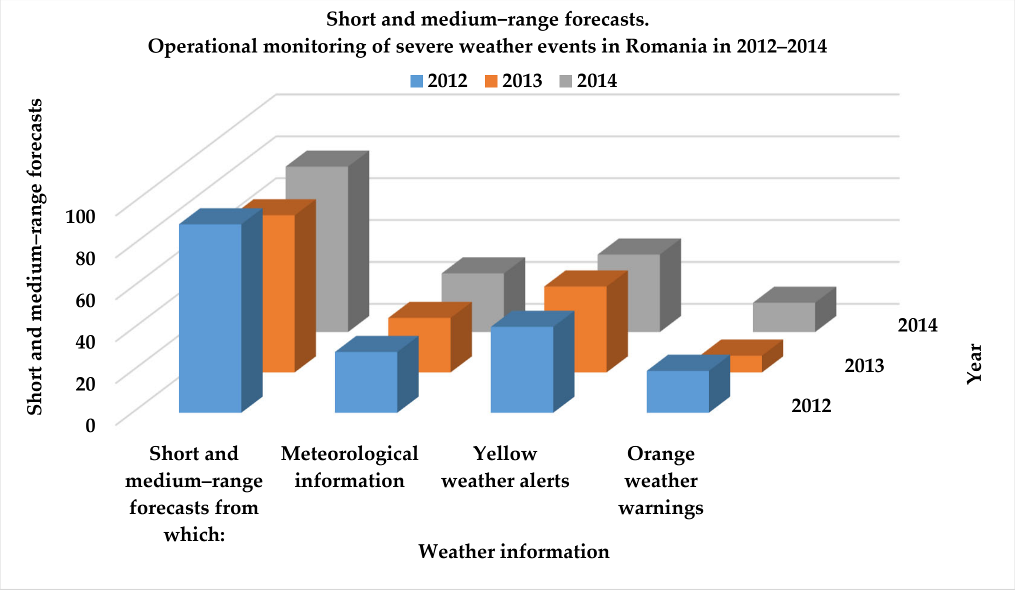

a)

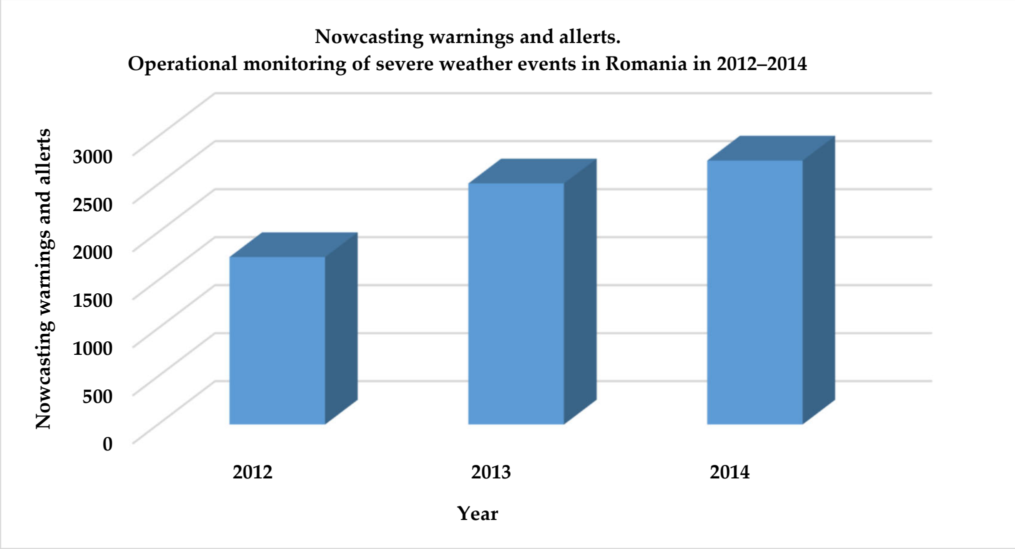

b)

Figure S.1.1. Operational monitoring of weather events in Romania during the 2012–2014 period: (**a**) Short and medium-range forecasts (meteorological information, yellow weather alerts and orange weather warnings), (**b**) Nowcasting warnings and alerts.

Red code—the forecast meteorological phenomena (strong wind, heavy precipitation, atmospheric electric discharges, hail, heavy snow, blizzard, extreme temperatures, frost, fog) will be dangerous, with a very high degree of intensity and disastrous effects.
Orange code—the forecast meteorological phenomena (strong wind, heavy precipitation, atmospheric electric discharges, hail, heavy snow, blizzard, extreme temperatures, frost, fog) will be dangerous, with a high degree of intensity.
Yellow code—the forecast meteorological phenomena (strong wind, heavy precipitation, atmospheric electric discharges, hail, heavy snow, blizzard, extreme temperatures, frost, fog) will be temporarily dangerous for certain activities, but otherwise are common for the period or area specified.
Green code—dangerous weather events do not forecast.
Nowcasting (immediate forecast): a description of the weather parameters for the next interval of maximum of 3 hours (*Source:* http://www.meteoromania.ro/avertizari/)

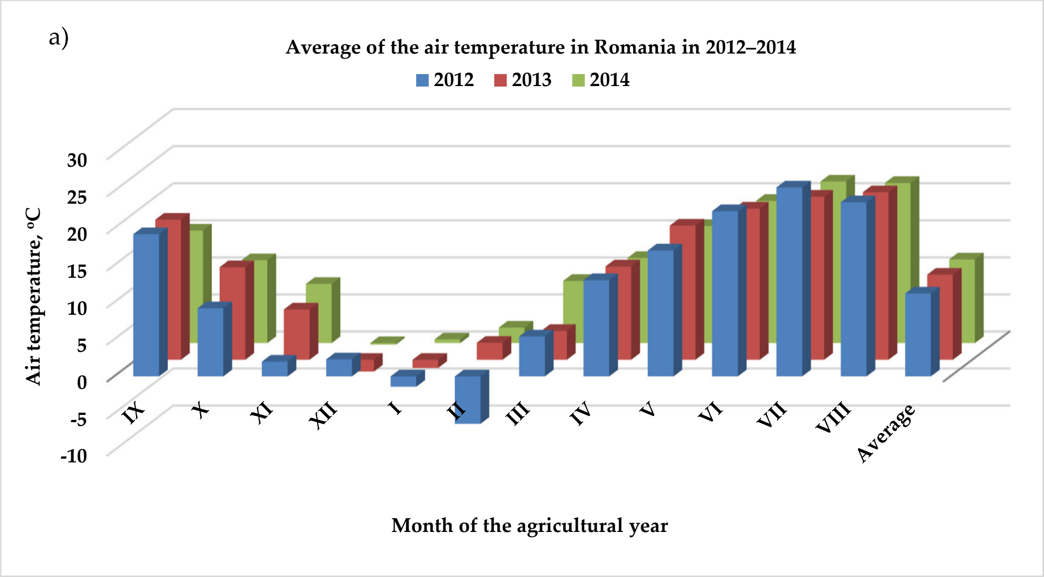

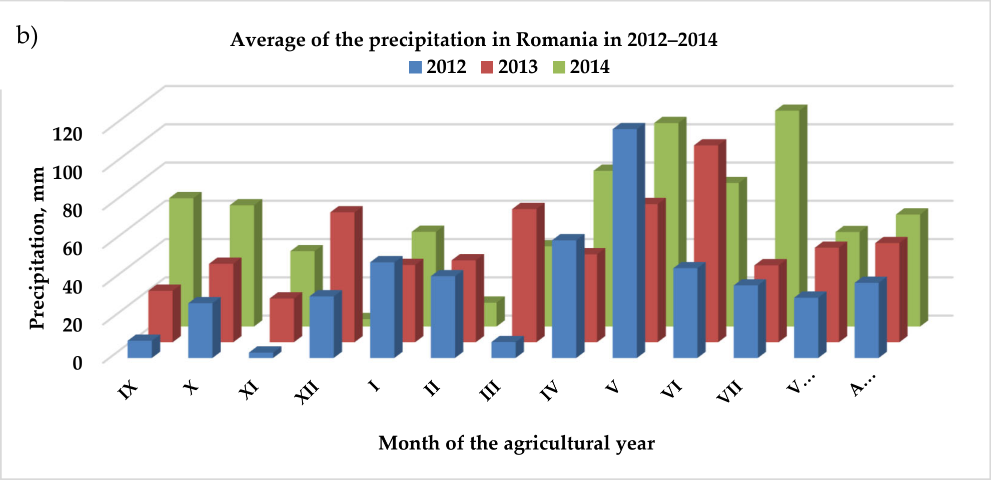

Figure S.1.2. Average weather parameters in Romania during the 2012–2014 period with extreme weather events: (**a**) Air temperature, (**b**) Precipitation.

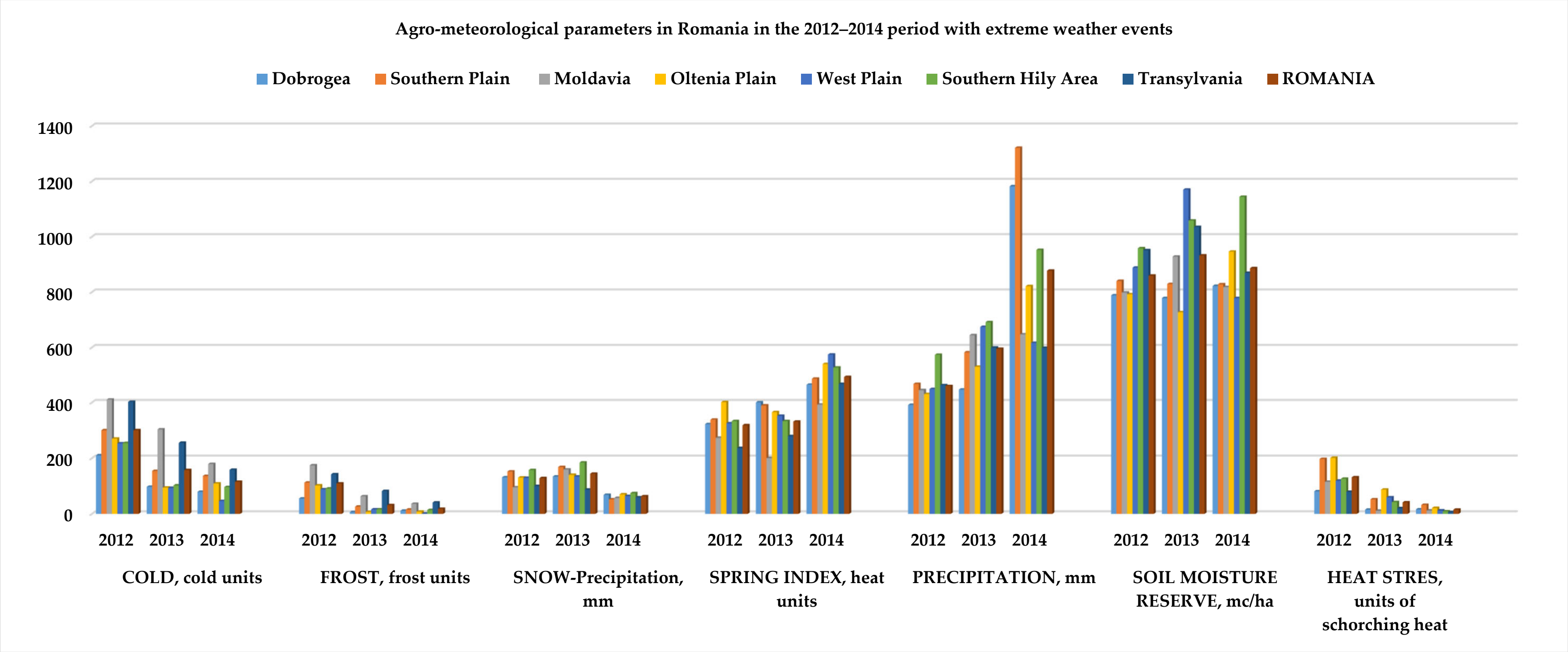

Figure S.1.3. Agro-meteorological parameters (Cold, frost, snow-precipitation, spring index, precipitation, soil moisture reserve, heat stress, and air temperature) in Romania during the 2012–2014 period with extreme weather events.

Cold = sum of daily minimum temperature ≤ 0 °C (1st November – 31st March), cold units; Frost = sum of daily minimum temperature ≤ −10–−15 °C (1st December – 28th February), frost units; Snow–Precipitation = sum of precipitation amounts as snow (1st November – 31st March), mm; Spring index = sum of average daily temp. ≥ 0 °C (1st March– 10th April), heat units; Air Temperature, °C; Heat Stress = sum of daily maximum temperature ≥ 32 °C (1st June – 31st August), units of scorching heat; Precipitation amounts by year = sum of precipitation amounts (1st September – 31st August), mm

S.2: Deoxynivalenol (DON) occurrence in the triticale crops in Romania during the 2012–2014 period with extreme weather events

The limit of DON ≥1000 μg/kg was used in order to evaluate the influence of extreme weather events and agroclimatic factors on the natural deoxynivalenol occurrence in triticale crops in the 2012–2014 period with extreme weather events.

The use of the maximum limit of 1250 μg/kg, according to EC Regulation no. 1881/2006, would have omitted triticale samples with deoxynivalenol between 1000 μg/kg and 1250 μg/kg, resulting from the soil leaching process or the reduction of the contamination level (the synergistic effect of the extreme precipitation and floods in May–July 2014, the soil types and the historical aridity indices on the deviated route of ''Vb'' cyclones in southern Romania and Southeastern Europe).

Table S.2.1. Deoxynivalenol (DON) occurrence in the triticale crop by county, agricultural region, geographic position, historical aridity indices and agricultural year in Romania in the 2012–2014 period with extreme weather events.

County and Agricultural Region	Geographic Position		Soil type	Aridity Indices, 1900–2000		Deoxynivalenol (DON) Occurrence in the Triticale Crop by County, Agricultural Region, Geographic Position, Historical Aridity Indices and Agricultural Year in Romania in the 2012–2014 Period with Extreme Weather Events			
						2012			
	Latitude, °N	Longitude, °E		Iar-dM, mm °C^{-1}	CWD, mm	Analysed	Positive, %	≥1000 µg/kg, %	Interval<br>Average ± SD, µg/kg
**Maramureș**	47.40	24.00	Lu	50	0	2	2<br>100	0<br>0	169.68 – 174.25<br>171.97 ± 3.23
**Sălaj**	47.12	23.02	Lu	50	0	3	2<br>66.7	1<br>33.3	<18.50 – 3378.44<br>1220.28 ± 1873.04
**Bistrița-Năsăud**	47.08	24.30	Lu	60	0	-	-	-	-
**Mureș**	46.35	24.37	Ph	40	−100	3	3<br>100	1<br>33.3	170.75 – 3170.89<br>1373.41 ± 1586.05
**Harghita**	46.22	25.48	Lu	40	0	2	2<br>100	0<br>0	21.16 – 32.12<br>26.64 ± 7.75
**Covasna**	45.54	26.02	Lu	60	0	-	-	-	-
**Brașov**	45.47	25.17	Lu	40	0	3	3<br>100	0<br>0	48.18 – 238.05<br>145.83 ± 95.05
**Sibiu**	45.00	24.00	Lu	40	0	1	1<br>100	0<br>0	858.80
**Alba**	46.46	23.35	Lu	30	−100	2	2<br>100	0<br>0	65.45 – 111.54<br>88.50 ± 32.59
**Cluj**	46.46	23.35	Ph	30	−100	2	2<br>100	0<br>0	70.73 – 89.04<br>79.89 ± 12.95
**Hunedoara**	45.47	22.56	Lu	40	−50	2	2<br>100	1<br>50	120.26 – 1597.04<br>858.65 ± 1044.24
**TRANSYLVANIA**	**46.23**	**24.15**		**44**	**−32**	**20**	**19**<br>**95**	**3**<br>**15**	**<18.50 – 3378.40**<br>**576.43 ± 1000.08**
**Buzău**	45.27	26.77	Ch	30	−200	-	-	-	-
**Prahova**	45.02	26.02	Lu	30	−100	1	1<br>100	0<br>0	207.70
**Dâmbovița**	44.53	25.28	Lu	40	−100	2	2<br>100	0<br>0	134.49 – 498.08<br>316.29 ± 257.10

County and Agricultural Region	2013				2014			
	Analysed	Positive, %	≥1000 µg/kg,%	Interval<br>Average ± SD, µg/kg	Analysed	Positive, %	≥1000 µg/kg, %	Interval<br>Average ± SD, µg/kg
**Maramureș**	2	2<br>100	1<br>50	368.99 – 1961.94<br>1165.47 ± 1126.39	3	3<br>100	0<br>0	26.57 – 51.19<br>40.00 ± 12.46
**Sălaj**	4	3<br>75	3<br>75	<18.50 – 3106.44<br>1678.17 ± 1285.28	3	3<br>100	0<br>0	237.86 – 776.42<br>515.49 ± 269.67
**Bistrița-Năsăud**	1	0<br>0	0<br>0	<18.50	2	2<br>100	0<br>0	79.33 – 106.41<br>92.87 ± 19.15
**Mureș**	6	6<br>100	0<br>0	24.69 – 275.91<br>177.93 ± 103.09	6	6<br>100	3<br>50	136.33 – 2165.68<br>1071.61 ± 922.45
**Harghita**	2	1<br>50	0<br>0	<18.50 – 46.41<br>32.46 ± 19.74	1	1<br>100	0<br>0	126.83
**Covasna**	1	1<br>100	0<br>0	285.21	-	-	-	-
**Brașov**	-	-	-	-	1	1<br>100	0<br>0	163.26 – 201.35<br>185.58 ± 19.87
**Sibiu**	1	1<br>100	0<br>0	306.96	2	1<br>50	0<br>0	780.15 – 793.18<br>786.67 ± 9.21
**Alba**	1	1<br>100	0<br>0	82.94	1	1<br>100	0<br>0	346.86
**Cluj**	2	1<br>50	0<br>0	<18.50 – 241.14<br>129.82 ± 157.43	1	1<br>100	0<br>0	838.53
**Hunedoara**	2	1<br>50	0<br>0	21.91 – 307.14<br>164.53 ± 201.69	1	1<br>100	1<br>50	2399.56
**TRANSYLVANIA**	**22**	**18**<br>**81.8**	**4**<br>**18.2**	**<18.50 – 3106.40**<br>**520.84 ± 840.07**	**23**	**23**<br>**100**	**4**<br>**17.4**	**26.57 – 2399.60**<br>**614.08 ± 719.10**
**Buzău**	-	-	-	-	-	-	-	-
**Prahova**	2	1<br>50	0<br>0	<18.50 – 21.82<br>20.16 ± 2.35	1	1<br>100	1<br>100	465.29 – 1006.77<br>736.03 ± 382.88
**Dâmbovița**	2	1<br>50	0<br>0	<18.50 – 21.02<br>19.76 ± 1.78	3	3<br>100	3<br>100	1522.56 – 2853.78<br>2080.88 ± 691.07

County and Agricultural Region	2012–2014			
	Analysed	Positive, %	≥1000 µg/kg,%	Interval<br>Average ± SD, µg/kg
**Maramureș**	7	7<br>100	1<br>14.3	26.57 – 1961.94<br>399.27 ± 699.25
**Sălaj**	10	8<br>80	4<br>40	<18.50 – 3378.44<br>1192.00 ± 1266.61
**Bistrița-Năsăud**	3	2<br>66.7	0<br>0	<18.50 – 106.41<br>68.08 ± 45.02
**Mureș**	6	6<br>100	4<br>26.7	24.69 – 3170.89<br>774.50 ± 966.58
**Harghita**	5	4<br>80	0<br>0	<18.50 – 126.83<br>49.00 ± 44.87
**Covasna**	1	1<br>100	0<br>0	285.21
**Brașov**	4	4<br>100	0<br>0	48.18 – 238.05<br>165.71 ± 65.16
**Sibiu**	4	3<br>75	0<br>0	306.96 – 858.80<br>684.77 ± 254.22
**Alba**	4	4<br>100	0<br>0	65.45 – 346.86<br>151.70 ± 131.49
**Cluj**	5	4<br>80	0<br>0	<18.50 – 838.53<br>251.59 ± 338.40
**Hunedoara**	5	4<br>80	2<br>40	21.91 – 2399.56<br>889.18 ± 1056.47
**TRANSYLVANIA**	**65**	**60**<br>**92.3**	**11**<br>**16.9**	**<18.50 – 3378.44**<br>**570.94 ± 841.30**
**Buzău**	-	-	-	-
**Prahova**	4	3<br>75	1<br>25	<18.50 – 1006.77<br>344.02 ± 413.01
**Dâmbovița**	7	6<br>85.7	3<br>42.9	<18.50 – 2853.78<br>987.82 ± 1109.19

County and Agricultural Region	Geographic Position		Soil type	Aridity Indices, 1900–2000		Deoxynivalenol (DON) Occurrence in the Triticale Crop by County, Agricultural Region, Geographic Position, Historical Aridity Indices and Agricultural Year in Romania in the 2012–2014 Period with Extreme Weather Events															
						2012				2013				2014				2012–2014			
	Latitude, °N	Longitude, °E		Iar-dM, mm °C⁻¹	CWD, mm	Analysed	Positive, %	≥1000 µg/kg, %	Interval Average ± SD, µg/kg	Analysed	Positive, %	≥1000 µg/kg,%	Interval Average ± SD, µg/kg	Analysed	Positive, %	≥1000 µg/kg, %	Interval Average ± SD, µg/kg	Analysed	Positive, %	≥1000 µg/kg,%	Interval Average ± SD, µg/kg
**Argeș**	45.00	24.07	Lu	40	–100	2	2<br>100	0<br>0	75.83 – 353.57<br>214.70 ± 196.39	3	0<br>0	0<br>0	<18.50	2	2<br>100	2<br>100	3264.41 – 3592.66<br>3428.54 ± 232.11	7	4<br>57.4	2<br>28.6	<18.50 – 3592.66<br>1048.85 ± 1632.72
**Vâlcea**	45.05	24.07	Lu	40	–100	1	1<br>100	0<br>0	289.55	2	1<br>50	0<br>0	<18.50 – 26.14<br>22.32 ± 5.40	1	1<br>100	1<br>100	1280.45	4	3<br>75	1<br>25	<18.50 – 1280.45<br>403.66 ± 154.33
**Gorj**	45.02	23.18	Lu	50	0	3	2<br>66.7	0<br>0	<18.50 – 473.33<br>211.28 ± 235.20	4	3<br>75	0<br>0	<18.50 – 245.55<br>101.31 ± 103.73	2	2<br>100	0<br>0	105.74 – 135.98<br>120.86 ± 21.38	9	7<br>77.8	0<br>0	<18.50 – 473.33<br>142.31 ± 143.74
**Caraș Severin**	45.09	22.04	Lu	40	–50	3	2<br>66.7	0<br>0	50.31 – 245.50<br>165.17 ± 102.07	3	1<br>33.3	0<br>0	<18.50 – 31.18<br>22.73 ± 7.32	2	2<br>100	0<br>0	216.55 – 663.16<br>439.86 ± 315.80	8	5<br>62.5	0<br>0	<18.50 – 663.16<br>180.43 ± 217.32
**SOUTHERN HILLY AREA**	**45.00**	**24.49**		**39**	**–93**	**12**	**11<br>91.7**	**0<br>0**	**<18.50 – 498.08<br>224.05 ± 156.31**	**16**	**7<br>43.8**	**0<br>0**	**<18.50 – 245.55<br>40.84 ± 58.86**	**11**	**11<br>100**	**7<br>63.6**	**105.74 – 3592.70<br>1426.65 ± 1298.71**	**39**	**29<br>74.4**	**7<br>17.9**	**<18.50 – 3592.66<br>488.08 ± 901.96**
**Timiș**	45.47	21.21	Ch	40	–200	2	2<br>100	0<br>0	226.94 – 316.44<br>271.69 ± 63.29	3	2<br>66.7	0<br>0	<18.50 – 53.21<br>34.36 ± 17.55	3	3<br>100	2<br>66.7	450.95 – 1198.34<br>893.69 ± 392.36	8	7<br>87.5	2<br>25	<18.50 – 1198.34<br>415.94 ± 459.14
**Arad**	46.22	21.48	Ch	30	–200	3	2<br>66.7	0<br>0	<18.50 – 47.29<br>34.32 ± 14.60	3	3<br>100	0<br>0	38.78 – 156.41<br>90.32 ± 60.15	4	3<br>75	0<br>0	<18.50 – 312.43<br>198.32 ± 132.92	10	8<br>80	0<br>0	<18.50 – 312.43<br>137.68 ± 114.50
**Bihor**	47.04	21.55	Lu	30	–100	2	2<br>100	0<br>0	97.61 – 399.89<br>248.75 ± 213.74	1	1<br>100	0<br>0	661.73	2	2<br>100	0<br>0	<18.50 – 104.47<br>61.49 ± 60.79	5	5<br>80	0<br>0	<18.50 – 661.73<br>220.58 ± 296.68
**Satu-Mare**	47.47	22.53	Lu	30	–150	1	1<br>100	0<br>0	161.76	-	-	-	-	2	1<br>50	0<br>0	141.16 – 218.82<br>179.99 ± 54.91	3	2<br>66.7	0<br>0	141.16 – 218.82<br>179.99 ± 54.91
**WEST PLAIN**	**46.55**	**21.69**		**33**	**–163**	**8**	**7<br>87.5**	**0<br>0**	**<18.50 – 399.89<br>163.20 ± 140.47**	**7**	**6<br>85.7**	**0<br>0**	**<18.50 – 661.73<br>147.97 ± 231.12**	**11**	**9<br>82**	**2<br>18.2**	**<18.50 – 1198.30<br>359.75 ± 396.19**	**26**	**22<br>84.6**	**2<br>7.7**	**<18.50 – 1198.34<br>242.26 ± 302.81**
**Mehedinți**	44.58	22.53	Lu	50	–100	1	1<br>100	0<br>0	482.75	1	0<br>0	0<br>0	<18.50	1	1<br>100	0<br>0	24.91	3	2	0<br>0	<18.50 – 482.75<br>175.39 ± 266.20
**Dolj**	44.10	23.42	Ch	25	–200	2	1<br>50	0<br>0	<18.50 – 49.22<br>33.86 ± 21.72	1	1<br>100	0<br>0	47.56	3	3<br>100	0<br>0	253.70 – 690.00<br>526.86 ± 238.05	6	5<br>83.3	0<br>0	<18.50 – 690.00<br>282.64 ± 307.17
**Olt**	44.10	23.42	Ph	30	–200	3	2<br>66.7	0<br>0	<18.50 – 138.13<br>65.43 ± 63.84	5	1<br>20	0<br>0	<18.50 – 54.06<br>25.61 ± 15.90	3	3<br>100	1<br>33.3	156.11 – 1825.75<br>888.25 ± 853.55	11	6<br>54.6	1<br>9.1	<18.50 – 1825.75<br>271.74 ± 551.10
**OLTENIA PLAIN**	**44.26**	**23.12**		**35**	**–167**	**6**	**4<br>66.7**	**0<br>0**	**<18.50 – 482.75<br>124.46 ± 181.03**	**7**	**2<br>28.6**	**0<br>0**	**<18.50 – 54.06<br>27.73 ± 15.88**	**7**	**7<br>100**	**1<br>14.3**	**24.91 – 1825.80<br>610.03 ± 600.80**	**20**	**13<br>65**	**1<br>5**	**<18.50 – 1825.75<br>260.56 ± 439.90**
**Suceava**	47.39	26.15	Lu	30	–100	1	1<br>100	0<br>0	390.42	-	-	-	-	1	1<br>100	0<br>0	329.70	2	2<br>100	0<br>0	329.70 – 390.42<br>360.06 ± 42.94
**Neamț**	46.58	26.24	Lu	30	–200	2	2<br>100	0<br>0	272.70 – 394.74<br>333.72 ± 86.30	2	2<br>100	0<br>0	148.76 – 200.02<br>174.39 ± 36.25	2	2<br>100	1<br>50	406.24 – 1301.69<br>853.97 ± 633.18	6	6<br>100	1<br>16.7	148.76 – 1301.69<br>454.03 ± 427.77

County and Agricultural Region	Geographic Position		Soil type	Aridity Indices, 1900–2000		Deoxynivalenol (DON) Occurrence in the Triticale Crop by County, Agricultural Region, Geographic Position, Historical Aridity Indices and Agricultural Year in Romania in the 2012–2014 Period with Extreme Weather Events															
						2012				2013				2014				2012–2014			
	Latitude, °N	Longitude, °E		Iar-dM, mm °C⁻¹	CWD, mm	Analysed	Positive, %	≥1000 μg/kg, %	Interval Average ± SD, μg/kg	Analysed	Positive, %	≥1000 μg/kg,%	Interval Average ± SD, μg/kg	Analysed	Positive, %	≥1000 μg/kg, %	Interval Average ± SD, μg/kg	Analysed	Positive, %	≥1000 μg/kg,%	Interval Average ± SD, μg/kg
**Bacău**	46.25	26.47	Lu	30	–200	-	-	-	-	2	2<br>100	0<br>0	57.00 – 65.85<br>61.43 ± 6.26	2	2<br>100	0<br>0	178.42 – 413.08<br>295.75 ± 165.93	4	4<br>100	0<br>0	57.00 – 413.08<br>178.59 ± 165.81
**Vrancea**	45.47	26.58	Ch	25	–200	1	1<br>100	0<br>0	124.73	2	2<br>100	0<br>0	39.43 – 40.61<br>40.02 ± 0.83	1	1<br>100	1<br>100	1041.67	4	4<br>100	1<br>25	39.43 – 1041.67<br>311.61 ± 488.34
**Botoșani**	47.50	26.49	Ch	30	–100	1	1<br>100	0<br>0	367.48	-	-	-	-	1	1<br>100	1<br>100	1533.76	2	2<br>100	1<br>50	367.48 – 1533.76<br>950.62 ± 824.68
**Iași**	47.15	27.19	Ch	30	–200	2	1<br>50	0<br>0	<18.50 – 20.85<br>19.68 ± 1.66	3	3<br>100	0<br>0	223.16 – 380.02<br>313.12 ± 80.93	2	2<br>100	0<br>0	188.37 – 365.54<br>276.96 ± 125.28	7	6<br>85.7	0<br>0	<18.50 – 380.02<br>218.95 ± 153.60
**Vaslui**	46.35	27.46	Ch	25	–250	-	-	-	-	1	0<br>0	0<br>0	<18.50	3	3<br>100	0<br>0	151.75 – 685.31<br>374.49 ± 277.47	4	3<br>75	0<br>0	<18.50 – 685.31<br>285.49 ± 288.11
**Galați**	45.47	27.47	Ch	25	–300	2	0<br>0	0<br>0	<18.50	3	2<br>66.7	0<br>0	<18.50 – 410.56<br>189.19 ± 200.88	1	1<br>100	0<br>0	426.60	6	3<br>50	0<br>0	<18.50 – 426.60<br>171.86 ± 196.74
**MOLDAVIA**	**46.52**	**26.76**		**28**	**–194**	**9**	**6<br>66.7**	**0<br>0**	**<18.50 – 394.74<br>180.71 ± 173.48**	**13**	**11<br>84.6**	**0<br>0**	**<18.50 – 410.56<br>159.78 ± 140.45**	**13**	**13<br>100**	**3<br>23.1**	**151.75 – 1533.80<br>562.20 ± 449.54**	**35**	**30<br>85.7**	**3<br>8.6**	**<18.50 – 1533.76<br>314.63 ± 350.31**
**Teleorman**	43.39	25.21	Ch	30	–250	3	3<br>100	0<br>0	88.22 – 145.44<br>107.47 ± 32.88	3	0<br>0	0<br>0	<18.50	4	4<br>100	2<br>50	24.37 – 1924.29<br>835.65 ± 947.36	10	7<br>70	2<br>20	<18.50 – 1924.29<br>372.05 ± 678.18
**Giurgiu**	44.10	25.54	Ch	25	–200	2	0<br>0	0<br>0	<18.50	1	0<br>0	0<br>0	<18.50	3	3<br>100	3<br>100	1064.81 – 1250.76<br>1132.91 ± 102.47	6	3<br>50	3<br>50	<18.50 – 1250.76<br>575.71 ± 613.82
**Ilfov**	44.37	26.07	Lu	25	–200	-	-	-	-	1	1<br>100	0<br>0	47.58	4	3<br>75	0<br>0	127.66 – 258.60<br>184.78 ± 57.15	5	4<br>80	0<br>0	47.58 – 258.60<br>157.34 ± 78.83
**Călărași**	44.20	27.33	Ch	25	–300	3	3<br>100	0<br>0	20.06 – 246.55<br>132.43 ± 113.26	3	2<br>66.7	0<br>0	<18.50 – 59.03<br>41.44 ± 20.79	3	3<br>100	0<br>0	222.12 – 951.49<br>624.42 ± 370.46	9	8<br>88.9	0<br>0	<18.50 – 951.49<br>266.09 ± 333.77
**Ialomița**	44.38	27.18	Ch	25	–300	2	2<br>100	0<br>0	69.20 – 231.32<br>150.26 ± 114.64	1	0	0	<18.50	2	2<br>100	0<br>0	611.87 – 652.92<br>632.40 ± 29.03	5	4<br>80	0<br>0	<18.50 – 652.92<br>316.76 ± 299.01
**Brăila**	45.07	27.41	Ch	25	–300	3	3	0<br>0	33.06 – 85.80<br>63.20 ± 27.17	2	2	0<br>0	41.01 – 226.64<br>133.83 ± 131.26	5	5<br>100	0<br>0	254.35 – 328.53<br>295.42 ± 29.96	10	10<br>100	0<br>0	33.06 – 328.53<br>193.48 ± 121.24
**SOUTHERN PLAIN**	**44.34**	**26.57**		**26**	**–258**	**13**	**11<br>84.6**	**0<br>0**	**<18.50 – 246.55<br>95.91 ± 75.24**	**11**	**5<br>45.5**	**0<br>0**	**<18.50 – 226.64<br>48.37 ± 61.08**	**21**	**21<br>100**	**5<br>23.8**	**24.37 – 1924.30<br>575.98 ± 510.96**	**45**	**37<br>82.2**	**5<br>11.1**	**<18.50 – 1924.29<br>308.32 ± 430.68**
**Tulcea**	45.00	28.00	Ch	20	–350	1	1<br>100	0<br>0	67.02	1	1<br>100	0<br>0	19.43	2	2<br>100	1<br>50	74.45 – 1326.36<br>741 ± 885.23	4	4<br>100	1<br>25	19.43 – 1326.36<br>371.82 ± 636.83
**Constanța**	44.16	28.19	Ch	20	–400	-	-	-	-	1	0<br>0	0<br>0	<18.50	1	1<br>100	0<br>0	59.40	2	1<br>50	0<br>0	<18.50 – 59.40<br>38.95 ± 28.92

County and Agricultural Region	Geographic Position		Soil type	Aridity Indices, 1900–2000		Deoxynivalenol (DON) Occurrence in the Triticale Crop by County, Agricultural Region, Geographic Position, Historical Aridity Indices and Agricultural Year in Romania in the 2012–2014 Period with Extreme Weather Events															
						2012				2013				2014				2012–2014			
	Latitude, °N	Longitude, °E		Iar-dM, mm °C^{-1}	CWD, mm	Analysed	Positive, %	≥1000 µg/kg, %	Interval Average ± SD, µg/kg	Analysed	Positive, %	≥1000 µg/kg,%	Interval Average ± SD, µg/kg	Analysed	Positive, %	≥1000 µg/kg, %	Interval Average ± SD, µg/kg	Analysed	Positive, %	≥1000 µg/kg,%	Interval Average ± SD, µg/kg
**DOBROGEA**	**44.58**	**28.10**		**20**	**–375**	**1**	**1**<br>**100**	**0**<br>**0**	**67.02**	**2**	**1**<br>**50**	**0**<br>**0**	**<18.50 – 19.43**<br>**18.97 ± 0.66**	**3**	**3**<br>**100**	**1**<br>**33**	**59.40 – 1326.40**<br>**486.74 ± 727.17**	**6**	**5**<br>**83.3**	**1**<br>**16.6**	**<18.50 – 1326.36**<br>**260.86 ± 522.54**
**ROMANIA**	**45.35**	**24.98**		**32**	**–183**	**69**	**59**<br>**85.5**	**3**<br>**4.3**	**<18.50 – 3378.40**<br>**278.40 ± 575.30**	**78**	**50**<br>**64.1**	**4**<br>**5.1**	**<18.50 – 3106.40**<br>**205 ± 492.83**	**89**	**87**<br>**96.7**	**23**<br>**25.6**	**<18.50 – 3592.70**<br>**661.90 ± 742.90**	**236**	**196**<br>**83.1**	**30**<br>**12.7**	**<18.50 – 3592.66**<br>**398.76 ± 651.89**

Ch— chernozem; Lu— luvisol; Ph—phaeozem.

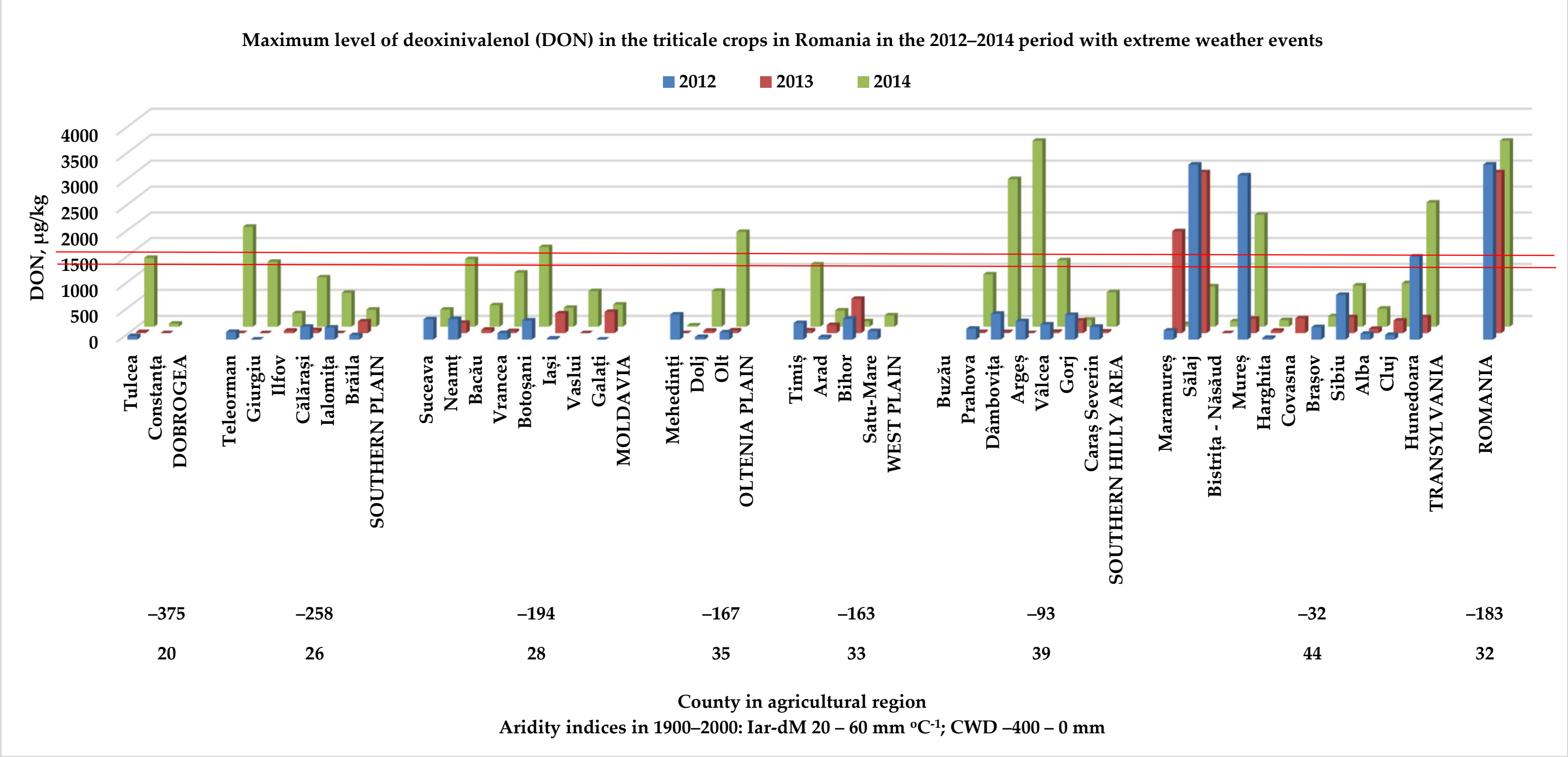

Figure S.2.1. Maximum level of deoxynivalenol (DON) in the triticale crop by county, agricultural region, historical aridity indices and agricultural year in Romania in the 2012–2014 period with extreme weather events. Red lines: maximum DON levels between 1000–1250 µg/kg.

Table S.2.2. Maximum level of deoxynivalenol (DON ≥1000 μg/kg)] in the triticale crops correlated with the geographic position (agricultural region, geographic coordinates, county and locality), aridity indices (de Martonne, Iar-dM; climatic water deficit, CWD), hydrographic basin and triticale variety in Romania in the 2012–2014 period with extreme weather events.

Agricultural Region	Geographic Coordinates	County	Aridity Indices, in 1900–2000		Locality	Hydrographic Basin	Triticale Variety	Deoxynivalenol and the de Martonne Index in 2012–2014			Precipitation at Meteorological Stations, mm			Air Temperature, °C			Meteo Station
			Iar-dM, mm °C-1	CWD, mm				DON, μg/kg	Iar-dM, mm °C-1	Year	May	June	July	May	June	July	
		Sălaj, SJ	50	0	Crasna	Valea Boului x Crasna	Trilstar	3378.44	24		96	96	28	16.5	21.1	24.1	Zalău - SJ
**Transylvania**	45–47 °N, 22–24 °E	Mureș, MS	40	–100	Târnăveni - Șeuca	Târnava Mică (trib. of Mureș)	Titan	3170.89	23	2012	159	58	22	15.4	20.7	23.3	Dumbrăveni - SB
		Hunedoara, HD	40	–50	Geoagiu	Geoagiu x Mureș	Titan	1597.04	26		157	78	57	16.1	20.9	24.2	Deva - HD
					Ileanda	Someș	Titan	1534.42	31		59	151	10	17.0	19.5	21.2	
**Transylvania**	45–47 °N, 22–24 °E	Sălaj, SJ	50	0	Zalău	Zalău (trib. of Crasna)	Haiduc	3106.44	31	2013	59	151	10	17.0	19.5	21.2	Zalău - SJ
					Năpradea	Someș	Titan	2053.30	31		59	151	10	17.0	19.5	21.2	
		Maramureș, MM	50	0	Șomcuta Mare	Bârsău (trib. of Someș)	Tremplin	1961.94	44		89	26	41	17.1	20.9	24.2	Baia Mare - MM
					Botorca	Târnava Mică (trib. of Mureș)	Stil	2067.50	30		131	62	110	14.5	17.5	19.9	Dumbrăveni - SB
**Transylvania**	45–47 °N, 22–24 °E	Mureș, MS	40	–100	Gheorghe Doja	Niraj (trib. of Mureș)	Titan	2165.68	30	2014	87	52	57	17.1	19.5	20.7	Tg. Mureș - MS
					Sângioargiu	Mureș	Cascador	1353.65	30		87	52	57	17.1	19.5	20.7	
		Hunedoara, HD	40	–50	Densuș	Densuș (trib. of Mureș)	Haiduc	2399.56	25		96	96	241	15.3	18.7	20.5	Caransebeș - CS
		Vâlcea, VL	40	–100	Diculești	Olteț (trib. of Olt)	Other	1280.45	55		179	150	205	15.7	19.2	21.7	Drăgășani - VL
**Southern Hilly Area**	45 °N, 22–26 °E	Argeș, AG	40	–100	Slobozia	Dâmbovnic (trib. of Neajlov – afl. of Argeș)	Other	3592.66	56	2014	143	122	240	15.7	19.3	21.6	Râmnicu Vâlcea - VL
					Buzoiești	Teleorman	Other	3264.41	56		143	122	240	15.7	19.3	21.6	
			40	–100	Dobra	Ialomița	Haiduc	2853.78	44		102	134	43	16.0	19.4	22.4	
		Dâmbovița, DB			Cornațelu	Teleorman (trib. of Vedea)	Gorun	1866.77	44		102	134	43	16.0	19.4	22.4	Băneasa - București
**Southern Hilly Area**	45 °N, 22–26 °E				Finta	Ialomița	Other	1522.56	44	2014	102	134	43	16.0	19.4	22.4	
		Prahova, PH	30	–100	Ploiești	Prahova (trib. of Ialomița)	Other	1006.77	38		117	71	110	16.0	19.3	22.5	Ploiești - PH
**West Plain**	45 °N, 21 °E	Timiș, TM	40	–200	Sânmihaiu Român	Bega	Silver	1198.34	30	2014	133	66	133	16.0	19.4	21.5	Lugoj - TM

Agricultural Region	Geographic Coordinates	County	Aridity Indices, in 1900–2000		Locality	Hydrographic Basin	Triticale Variety	Deoxynivalenol and the de Martonne Index in 2012–2014			Precipitation at Meteorological Stations, mm			Air Temperature, °C			Meteo Station
			Iar-dM, mm °C^{-1}	CWD, mm				DON, µg/kg	Iar-dM, mm °C^{-1}	Year	May	June	July	May	June	July	
					Fibiș	Beba Veche x Măgheruș (trib. of Bega)	Titan	1031.78	30		147	58	121	16.2	20.7	22.1	Timșoara - TM
**Oltenia Plain**	44 °N, 23 °E	Olt, OT	30	–200	Scornicești	Plapcea (trib. of Vedea)	Haiduc	1825.75	38	2014	132	66	182	15.8	19.3	22.0	Slatina - OT
**Moldavia**	45–47 °N, 26 °E	Botoșani, BT	30	–100	Bălușeni	Băiceni x Drăcșani	Tulus	1533.76	35	2014	144	19	172	16.1	18.6	21.1	Botoșani - BT
		Neamț, NT	30	–200	Roman	Siret x Moldova	Haiduc	1301.69	35		136	72	115	15.4	18.1	20.9	Roman - NT
		Vrancea, VR	25	–200	-	-	Other	1041.67	31		136	83	100	17.0	20.6	23.2	Focșani - VR
**Southern Plain**	43–44 °N, 25 °E	Teleorman	30	–250	Turnu Măgurele	Olt	Other	1924.29	65	2014	70	63	61	17.2	20.8	23.7	Turnu Măgurele - TR
					Ciolănești	Vedea	Other	1333.65	65		70	63	61	17.2	20.8	23.7	
		Giurgiu	25	–200	Stănești	Negrile x Ismar (trib. of Călniște)	Haiduc	1083.17	65		155	90	48	17.4	20.6	23.5	Giurgiu - GR
							Haiduc	1250.76	65		155	90	48	17.4	20.6	23.5	
							Haiduc	1064.81	65		155	90	48	17.4	20.6	23.5	
**Dobrogea**	45 °N, 28 °E	Tulcea, TL	20	–350	Sabangia	Danube Delta	Other	1326.36	47	2014	115	49	41	17.4	20.9	23.7	Tulcea - TL

trib.— tributary; affl.— affluent. The rivers of Transylvania and West Plains regions are the Tisza river's affluents, a significant affluent of the Danube River. The rivers of Southern Hilly Area, Oltenia Plain, the Southern Plain and Moldavia regions are affluents of the Danube River.

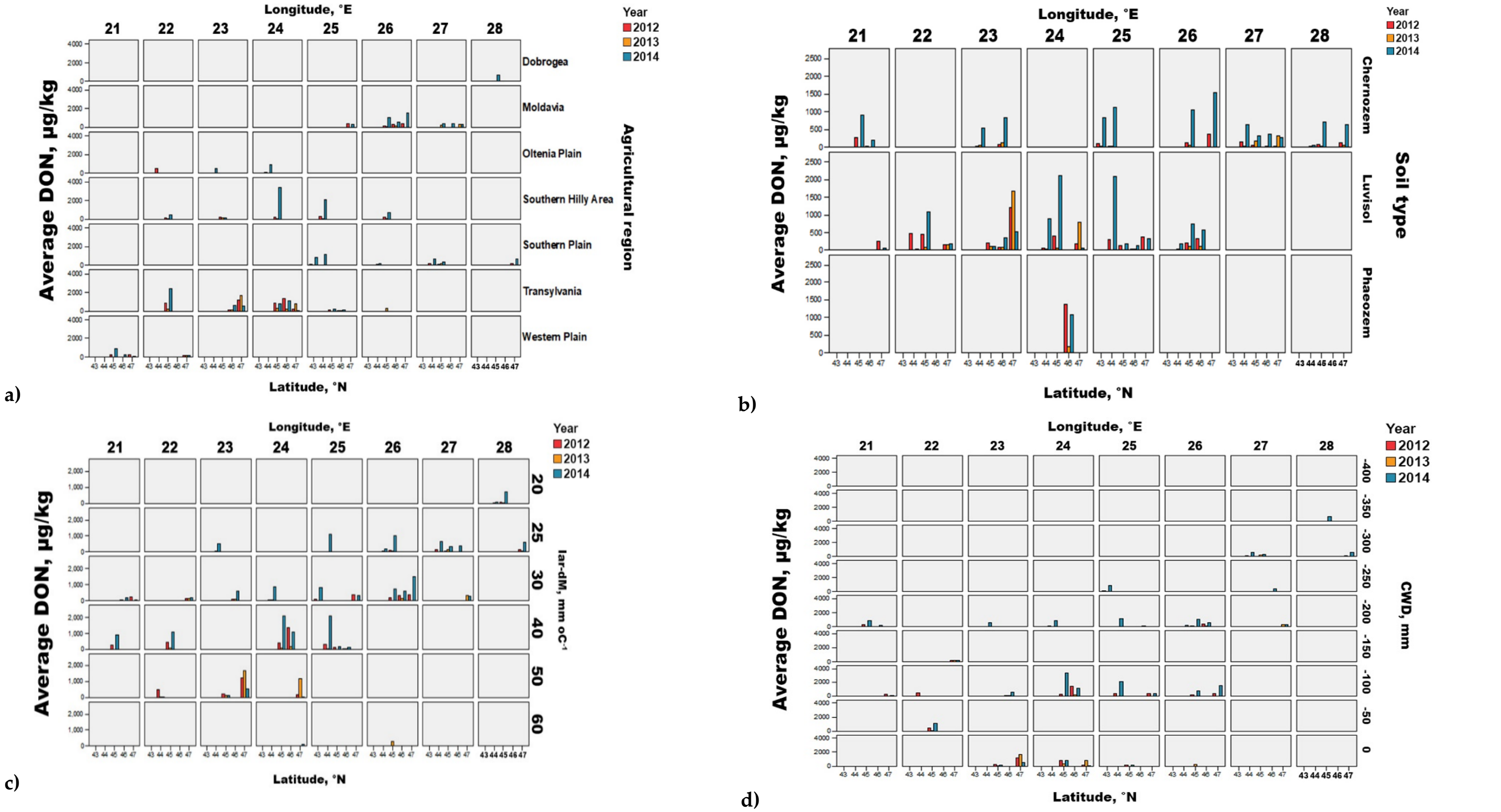

Figure S.2.2. Distribution of deoxynivalenol (DON) in the triticale crops in Romania in the 2012–2014 period with extreme weather events: (**a**) geographic coordinates (Northern latitude, °N and Eastern longitude, °E) and agricultural region; (**b**) soil type (Chernozem, phaeozem and luvisol), (**c**) historical de Martonne aridity index (Iar-dM, mm $^{o}C^{-1}$) and (**d**) historical climatic water deficit (CWD, mm) (graphical method).

Table S.2.3. Deoxynivalenol (DON) occurrence in the triticale crops by variety, agricultural region and agricultural year in Romania in the 2012–2014 period with extreme weather events.

Variety	2012							2013							2014							2012–2014							
	Deoxynivalenol (DON) Occurrence in the triticale Crops by Variety, Agricultural Region and Agricultural Year in Romania in the 2012–2014 Period with Extreme Weather Events																												
	Dobrogea	Southern Plain	Moldavia	Oltenia Plain	West Plain	Southern Hilly Area	Transylvania	Dobrogea	Southern Plain	Moldavia	Oltenia Plain	West Plain	Southern Hilly Area	Transylvania	Dobrogea	Southern Plain	Moldavia	Oltenia Plain	West Plain	Southern Hilly Area	Transylvania	Dobrogea	Southern Plain	Moldavia	Oltenia Plain	West Plain	Southern Hilly Area	Transylvania	2012–2014
**Mungis**	-	-	-	-	98 0/1	-	-	-	-	57 0/1	-	-	-	-	-	-	-	-	<18–105 0/2	-	-	-	-	57 0/1	-	<18–105 0/3	-	-	57–105 0/4
**Odisej**	-	-	-	-	-	-	-	-	-	-	-	<18 0/1	<18 0/2	-	-	-	-	-	-	-	-	-	-	-	-	<18 0/1	<18 0/2	-	<18.5 0/3
**Polego**	-	-	-	-	-	-	174 0/1	-	-	-	-	-	-	<18–369 0/2	-	-	-	-	-	-	51–106 0/3	-	-	-	-	-	-	<18–369 0/6	<18.5–369 0/6
**Amarillo**	-	-	-	-	-	50 0/1	-	-	-	-	-	-	-	-	-	-	-	-	-	-	-	-	-	-	-	-	50 0/1	-	50 0/1
**Gorun**	-	-	-	-	-	-	-	-	-	<18 0/1	<18 0/1	76 0/1	21 0/1	-	-	329 0/1	286 0/1	683 0/1	<18–179 0/2	1866 1/1	-	-	329 0/1	<18–286 0/2	<18–683 0/2	<18–179 0/3	21–1866 1/2	-	<18.5–1866 1/10
**Gorun 1**	-	<18 0/2	-	-	47 0/1	246 0/1	-	-	-	-	-	-	-	-	-	-	-	-	-	-	-	-	<18 0/2	-	-	47 0/1	246 0/1	-	<18.5–246 0/4
**Haiduc**	-	69–71 0/2	<18–395 0/5	138–483 0/2	-	-	71–171 0/3	-	<18–227 0/3	<18–380 0/8	<18–54 0/2	662 0/1	<18 0/3	<18–3106 1/5	-	276–1251 3/7	152–1302 1/5	25–1826 1/2	219 0/1	106–2854 1/2	238–2399 1/5	-	<18–1251 3/12	<18–1302 1/18	<18–1826 1/6	219–662 0/2	<18–2854 1/5	<18–3106 2/13	<18.5–3106 8/56
**Trilstar**	-	131–247 0/2	-	-	-		3378 1/1	-	<18–47 0/2	-	-	-	-	77 0/1	-	-	-	-	-	-	-	-	<18–247 0/4	-	-	-	-	77–3378 1/2	<18.5–3378 1/6
**Trismart**	-	-	-	-	-	-	-	-	-	-	-	-	-	-	-	-	-	-	141 0/1	-	27–553 0/2	-	-	-	-	141 0/1	-	553 0/2	27–553 0/3
**Cascador**	-	-	-	-	-	-	-	-	-	-	-	-	-	276 0/1	-	-	-	-	-	-	1354 1/1	-	-	-	-	-	-	1354 1/2	276–1354 1/2
**Stil**	67 0/1	-	-	-	-	208 0/1	48–779 0/3	-	-	39–41 0/2	-	156 0/1	-	220 0/1	-	303 0/1	-	-	-	-	136–2068 1/6	67 0/1	303 0/1	39–41 0/2	-	156 0/1	208 0/1	48–2068 1/10	39–2068 1/16
**Titan**	-	20 0/1	390 0/1	40 0/1	<18–162 0/3	<18–498 0/4	<18–3171 2/9	-	59 0/1	-	<18 0/2	39–53 0/2	34 0/1	<18–2053 2/9	-	-	-	156 0/1	284–1032 1/3	-	42–2166 1/4	-	20–59 0/2	390 0/1	<18–156 0/4	<18–1032 1/8	34–498 0/5	<18–3171 5/22	<18.5–3171 6/39
**Tulus**	-	-	-	-	-	-	-	-	-	-	-	-	-	-	-	-	1534 1/1	-	-	-	-	-	-	1534 1/1	-	-	-	-	1534 1/1

**Variety**	**Deoxynivalenol (DON) Occurrence in the triticale Crops by Variety, Agricultural Region and Agricultural Year in Romania in the 2012–2014 Period with Extreme Weather Events**																												
	**2012**							**2013**							**2014**							**2012–2014**							
	**Dobrogea**	**Southern Plain**	**Moldavia**	**Oltenia Plain**	**West Plain**	**Southern Hilly Area**	**Transylvania**	**Dobrogea**	**Southern Plain**	**Moldavia**	**Oltenia Plain**	**West Plain**	**Southern Hilly Area**	**Transylvania**	**Dobrogea**	**Southern Plain**	**Moldavia**	**Oltenia Plain**	**West Plain**	**Southern Hilly Area**	**Transylvania**	**Dobrogea**	**Southern Plain**	**Moldavia**	**Oltenia Plain**	**West Plain**	**Southern Hilly Area**	**Transylvania**	**2012–2014**
**Colina**	-	-	-	-	-	-	-	-	**48**<br>0/1	-	-	-	-	-	-	-	-	-	-	-	-	-	**48**<br>0/1	-	-	-	-	-	**48**<br>0/1
**Hercules**	-	-	-	-	-	**200**<br>0/1	-	-	-	-	-	-	-	-	-	-	-	-	-	-	-	-	-	-	-	-	**200**<br>0/1	-	**200**<br>0/1
**Plai**	-	-	-	-	-	**290**<br>0/1	**151**<br>0/1	-	-	-	-	-	**26**<br>0/1	-	-	-	-	-	-	-	**201**<br>0/1	-	-	-	-	-	**26–290**<br>0/2	**201**<br>0/1	**26–290**<br>0/3
**Silver**	-	-	-	-	-	-	-	-	-	-	-	**31**<br>0/1	-	**264**<br>0/1	-	-	**330**<br>0/1	-	**1198**<br>1/1	-	-	-	-	**330**<br>0/1	-	**31–1198**<br>**1**/2	-	**264**<br>0/1	**31–1198**<br>1/3
**Tarzan**	-	-	-	-	-	-	-	-	-	-	-	-	-	-	-	-	-	-	-	**663**<br>0/1	-	-	-	-	-	-	**663**<br>0/1	-	**663**<br>0/1
**Trialina**	-	-	-	-	-	-	-	-	-	-	-	-	**31**<br>0/1	-	-	-	-	-	-	-	-	-	-	-	-	-	**31**<br>0/1	-	**31**<br>0/1
**Trisidan**	-	-	-	-	**400**1	-	-	-	-	-	-	-	-	-	-	-	-	-	-	-	-	-	-	-	-	**400**<br>0/1	-	-	**400**<br>0/1
**Other**	-	<18–144<br>0/4	**<18–125**<br>**0/3**	**<18–49**<br>0/3	**<18–316**<br>0/2	**<18–473**<br>0/3	**<18–112**<br>0/2	**<18–19**<br>0/2	**<18–41**<br>0/4	**411**<br>**0/1**	**<18–48**<br>0/2	-	**<18–246**<br>0/7	-	**59–1326**<br>1/3	**24–1924**<br>2/12	**178–1042**<br>1/5	**254–690**<br>0/3	**451**<br>0/1	**136–3593**<br>5/8	**127**<br>**0/1**	**<18–1326**<br>**1**/5	**<18–1924**<br>**2**/20	**<18–1042**<br>**1**/9	**<18–690**<br>0/8	**<18–451**<br>0/3	**<18–3593**<br>**5**/18	**<18–127**<br>0/10	**<18.5–3593**<br>9/73
**ROMANIA**	**67**<br>0/1	**<18–247**<br>0/12	**<18–395**<br>0/9	**<18–483**<br>0/6	**<18–400**<br>0/8	**<18–498**<br>0/12	**<18–3378**<br>3/20	**<18**<br>0/2	**<18–227**<br>0/11	**<18–411**<br>0/13	**<18–54**<br>0/7	**<18–662**<br>0/7	**<18–246**<br>0/16	**<18–3106**<br>4/22	**59–1326**<br>1/3	**24–924**<br>5/21	**152–1534**<br>3/13	**25–1826**<br>1/7	**<18–1198**<br>2/11	**106–3593**<br>7/12	**27–2399**<br>4/23	**<18–1326**<br>**1**/6	**<18–1924**<br>**5**/44	**41–1534**<br>**3**/35	**<18–1826**<br>**1**/20	**<18–1198**<br>**2**/26	**<18–3593**<br>**7**/40	**<18–3106**<br>**11**/71	**<18.5–3593**<br>30/236

All <18 μg/kg DON values must be read as <18.50 μg/kg (LOD).

Table S.2.4. Deoxynivalenol (DON) occurrence in the triticale, winter wheat, durum wheat and rye crops in Romania in the 2012–2014 period with extreme weather events.

Crop *	Deoxynivalenol (DON) Occurrence in the Triticale, Winter Wheat, Durum Wheat and Rye Crops in Romania in the 2012–2014 Period with Extreme Weather Events. Interval; Average ± SD (Median); Positive samples; Samples with DON ≥1000 μg/kg		
	**2012**	**2013**	**2014**
**Winter Wheat**	<18.50 – 5027.74<br>178.56 ± 559.39 (37.23)<br>465/736 (63.18%)<br>25/736 (3.4%)	<18.50 – 3602.56<br>192.81 ± 309.45 (19.92)<br>420/816 (51.47%)<br>18/816 (2.21%)	<18.50 – 3025.72<br>209.43 ± 325.39 (84.04)<br>807/952 (84.77%)<br>36/952 (3.78%)
**Durum Wheat**	<18.50 – 402.35<br>133.18 ± 141.39 (95.41)<br>5/6 (83.33%)<br>0/6 (0%)	24.25 – 49.78<br>37.02 ± 18.05 (37.02)<br>2/2 (100%)<br>0/2 (0%)	25.10 – 483.99<br>228.14 ± 233.96 (175.34)<br>3/3 (100%)<br>0/3 (0%)
**Triticale**	<18.50 – 3378.44<br>282.23 ± 578.70 (127.70)<br>59/69 (85.51%)<br>3/69 (4.35%)	19.43 – 3106.44<br>204.98 ± 492.83 (40.02)<br>50/78 (64.10%)<br>4/78 (5.13%)	<18.50 – 3592.66<br>661.90 ± 742.90 (329.70)<br>87/89 (97.75%)<br>22/89 (24.72%)
**Rye**	<18.50 – 65.63<br>27.27 ± 17.23 (<18.50)<br>6/18 (33.33%)<br>0/18 (0%)	<18.50 – 360.70<br>69.48 ± 79.99 (29.72)<br>19/27 (70.37%)<br>0/27 (0%)	<18.50 – 1217.70<br>134.66 ± 251.55 (40.96)<br>23/30 (76.67%)<br>1/30 (3.33%)
**ROMANIA**	**<18.50 – 5027.74**<br>**183.46 ± 553.84 (41.95)**<br>**535/829 (64.54%)**<br>**28/829 (3.38%)**	**<18.50 – 3602.56**<br>**115.11 ± 325.44 (21.91)**<br>**491/923 (53.20%)**<br>**22/923 (2.38%)**	**<18.50 – 3592.66**<br>**244.89 ± 395.98 (93.26)**<br>**920/1074 (85.66%)**<br>**59/1074 (5.49%)**

Crop * — Data on DON occurrence in the winter wheat, durum wheat and rye crops in 2012–2014 were extracted from the ADER 8.1.1 project and have not been published so far. Winter wheat, durum wheat and rye crops were analysed simultaneously and in the same way as triticale crops. (LOD 18.50 μg/kg).

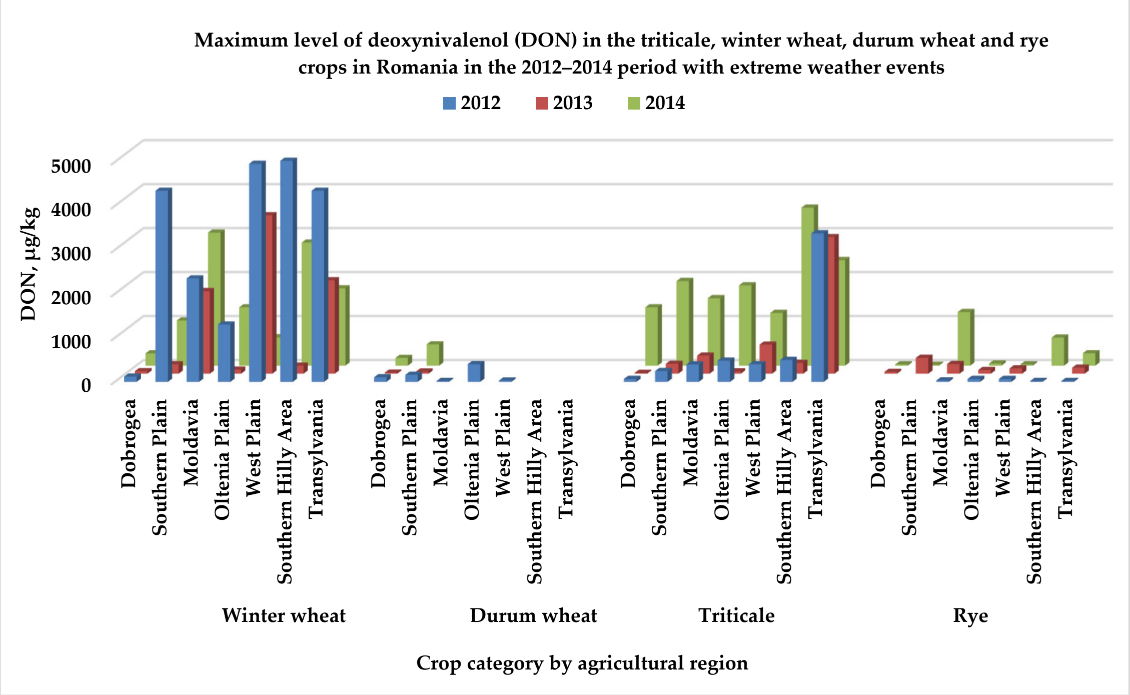

Figure S.2.3. Maximum level of deoxynivalenol (DON) in the triticale, winter wheat, durum wheat and rye crops in Romania in the 2012–2014 period with extreme weather events.

S.3: Statistical analysis of deoxynivalenol (DON) in the triticale crops in Romania during the 2012–2014 period with extreme weather events

Table S.3.1. Differences in the average air temperature by agricultural region and agricultural year in Romania in the 2012–2014 period with extreme weather events (ANOVA two-factors without replications).

Differences in the Average Air Temperature by Region and Year in Romania in 2012–2014						
**Source of Variation**	**SS**	**df**	**MS**	***F***	***p*-value**	**F crit.**
**Region**	12.03	6	2.01	29.82	0.000	3.00
**Year**	1.86	2	0.93	13.82	0.000	3.89
**Error**	0.81	12	0.07			
**Total**	14.70	20				

SS—sum of the squares; df—degrees of freedom; MS—the mean sum of squares; *F*—Fisher test; *p*-value—probability value; *F* crit.—the critical value of *F* test.

Table S.3.2. Class division of the average air temperatures by agricultural region in Romania in the 2012–2014 period with extreme weather events (*t*-test multiple).

Class Division	Agricultural Region	Average Air Temperature ± SD, °C	Differences in the Average Air Temperature by Region in Romania in 2012–2014					
			**Dif. 1**	**Dif. 2**	**Dif. 3**	**Dif. 4**	**Dif. 5**	**Dif. 6**
**I**	**Dobrogea**	12.24 ± 0.55	1.986 ***	1.848 ***	0.711 **	0.525 *	0.196 ns	0.149 ns
	**Southern Plain**	12.20 ± 0.38	1.837 ***	1.698 ***	0.562 *	0.375 ns	0.047 ns	0.000
	**Oltenia Plain**	12.05 ± 0.31	1.790 ***	1.652 ***	0.515 *	0.328 ns	0.000	
**II**	**West Plain**	11.72 ± 0.62	1.461 ***	1.323 ***	0.187 ns	0.000		
	**Southern Hilly Area**	11.53 ± 0.40	1.275 ***	1.137 ***	0.000			
**III**	**Moldavia**	10.40 ± 0.12	0.138 ns	0.000				
	**Transylvania**	10.26 ± 0.47	0.000					

DL 5% = 0.38, DL 1% = 0.57, DL 0.1% = 0.83

Dif.—difference; ns—non-significant difference; *—difference is significant at the 0.05 level (two-tailed); **—difference is significant at the 0.01 level (two-tailed); ***—difference is significant at the 0.001 level (two-tailed). DL—difference limit.

Table S.3.3. Class division of the average air temperature by agricultural year in Romania in the 2012–2014 period with extreme weather events (*t*-test multiple).

Class Division	Agricultural Year	Average Air Temperature ± SD, °C	Differences in the Average Air Temperature by Year in Romania in 2012–2014	
			Dif. 1	Dif. 2
I	2013	11.83 ± 0.95	0.729 ***	0.347 *
II	2014	11.48 ± 0.71	0.383 **	0.000
III	2012	11.10 ± 0.85	0.000	
DL 5% = 0.25, DL 1% = 0.37, DL 0.1% = 0.55				

Dif.—difference; ns—non-significant difference; *—difference is significant at the 0.05 level (two-tailed); **—difference is significant at the 0.01 level (two-tailed); ***—difference is significant at the 0.001 level (two-tailed). DL—difference limit.

Table S.3.4. Differences in the average cumulative precipitation by agricultural region and agricultural year in Romania in the 2012–2014 period with extreme weather events (ANOVA two-factors without replications).

Differences in the Average Cumulative Precipitation by Region and Year in Romania in 2012–2014						
Source of Variation	SS	df	MS	*F*	*p*-value	F crit.
Region	98710.82	6	16451.80	2.07	0.130	3.00
Year	212555.33	2	106277.67	13.34	0.000	3.89
Error	95595.40	12	7966.28			
Total	406861.56	20				

SS—sum of the squares; df—degrees of freedom; MS—the mean sum of squares; *F*—Fisher test; *p*-value—probability value; *F* crit.—the critical value of *F* test.

Table S.3.5. Class divisions of the cumulative average precipitation by agricultural region in Romania in the 2012–2014 period with extreme weather events (*t*-test multiple).

Class Division	Agricultural Region	Cumulative Average Precipitation, mm	Differences in the Cumulative Average Precipitation by Region in Romania in 2012–2014					
			Dif. 1	Dif. 2	Dif. 3	Dif. 4	Dif. 5	Dif. 6
I	Southern Hilly Area	724.28	208.88 *	185.40 *	150.09 *	147.35 *	142.63 *	107.86 ns
	Oltenia Plain	616.41	101.02 ns	77.54 ns	42.23 ns	39.48 ns	34.77 ns	0.00
II	Moldavia	581.65	66.25 ns	42.77 ns	7.47 ns	4.72 ns	0.00	
	Transylvania	576.93	61.54 ns	38.05 ns	2.75 ns	0.00		
	West Plain	574.18	58.79 ns	35.31 ns	0.00			
	Southern Plain	538.88	23.48 ns	0.00				
	Dobrogea	515.39	0.00					
DL 5% = 129.86, DL 1 % = 209.95, DL 0.1 % = 314.68								

Dif.—difference; ns—non-significant difference; *—difference is significant at the 0.05 level (two-tailed); **—difference is significant at the 0.01 level (two-tailed); ***—difference is significant at the 0.001 level (two-tailed). DL—difference limit.

Table S.3.6. Class division of the average cumulative precipitation by agricultural year in Romania in the 2012–2014 period with extreme weather events (*t*-test multiple).

Class Division	Agricultural Year	Average Cumulative Precipitation ± SD, mm	Differences in the Average Cumulative Precipitation by Year in 2012–2014

			Dif. 1	Dif. 2
**I**	**2014**	705.96 ± 153.45	244.574 ***	96.108 *
**II**	**2013**	609.85 ± 73.54	148.466 **	0.000
**III**	**2012**	461.38 ± 58.57	0.000	
DL 5% = 85.02, DL 1% = 127.91, DL 0.1% = 187.50				

Dif.—difference; ns—non-significant difference; *—difference is significant at the 0.05 level (two-tailed); **—difference is significant at the 0.01 level (two-tailed); ***—difference is significant at the 0.001 level (two-tailed); DL—difference limit.

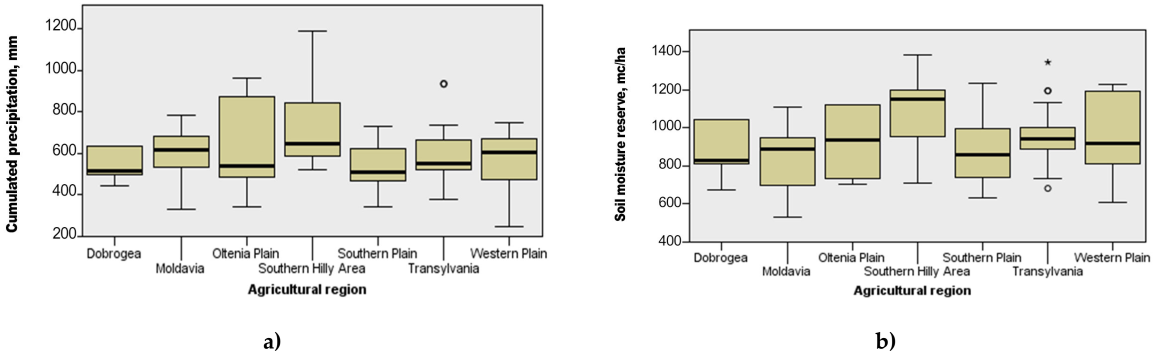

Figure S.3.1. Comparisons of the distribution of agrometeorological factors by agricultural region and agricultural year in Romania in the 2012–2014 period with extreme weather events: (**a**) Cumulative precipitation, mm; (**b**) Soil moisture reserve, mc/ha (Kruskal-Wallis non-parametric test for independent samples). ◦, ◦◦, and * ◦ — position of the extreme individual values.

Table S.3.7. Class divisions of the average deoxynivalenol (DON) by agricultural region in Romania in the 2012–2014 period with extreme weather events (*t*-test multiple).

Class Division	Agricultural Region	Average DON ± SD, μg/kg	Differences in the Averages Deoxynivalenol (DON) by Region in Romania in 2012–2014					
			Dif. 1	Dif. 2	Dif. 3	Dif. 4	Dif. 5	Dif. 6
**I**	**Transylvania**	570.94 ± 841.30	328.68 **	310.38 *	310.08 *	262.61 *	256.31 *	82.85 ns
	**Southern Hilly Area**	488.08 ± 901.96	245.82 ns	227.52 ns	227.22 ns	179.76 ns	173.45 ns	0.00
**II**	**Moldavia**	314.63 ± 350.31	72.37 ns	54.07 ns	53.77 ns	6.31 ns	0.00	
	**Southern Plain**	308.32 ± 430.68	66.06 ns	47.76 ns	47.46 ns	0.00		
	**Dobrogea**	260.86 ± 522.54	18.60 ns	0.30 ns	0.00			
	**Oltenia Plain**	260.56 ± 439.90	18.60 ns	0.00				
	**West Plain**	242.26 ± 302.81	0.00					
1st zone: DL 5% = 248.72, DL 1% = 327.40, DL 0.1% = 407.34								
2nd zone: DL 5% = 283.57, DL 1% = 373.27, DL 0.1% = 464.42								

Dif.—difference; *—the difference is significant at the 0.05 level (two-tailed); **—the difference is significant at the 0.01 level (two-tailed); ns—non-significant difference; DL—difference limit.

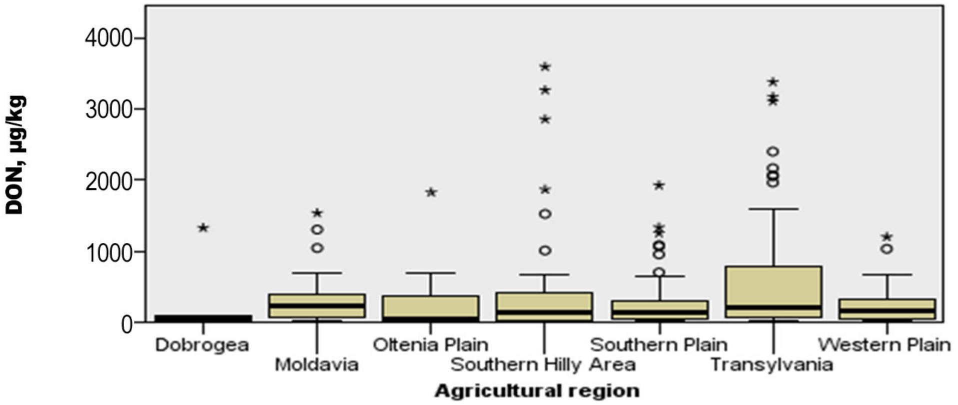

Figure S.3.2. Distribution of deoxynivalenol (DON, µg/kg) by agricultural region in Romania in the 2012–2014 period with extreme weather events (Kruskal-Wallis non-parametric test for independent samples). *, *◦ ◦, * * * *◦ ◦, * ** ◦◦ ◦ , * ** ◦ ◦◦◦, *◦ — position of the extreme individual values.

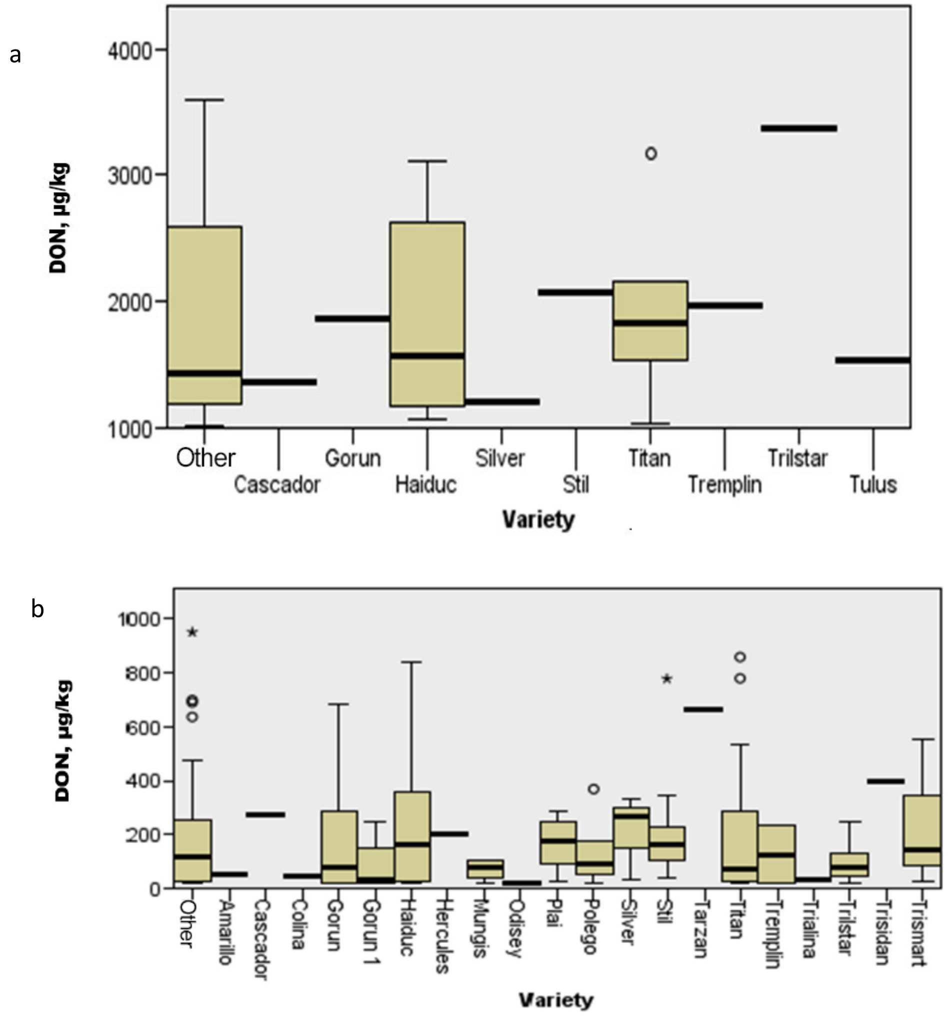

Figure S.3.3. Distribution of deoxynivalenol (DON) by triticale variety in Romania, in the 2012–2014 period with extreme weather events: (**a**) DON ≥1000 µg/kg; (**b**) DON ≤1000 µg/kg (Kruskal-Wallis non-parametric test for independent samples). ◦, ◦◦ ◦, ◦◦, and * — position of the extreme individual values.

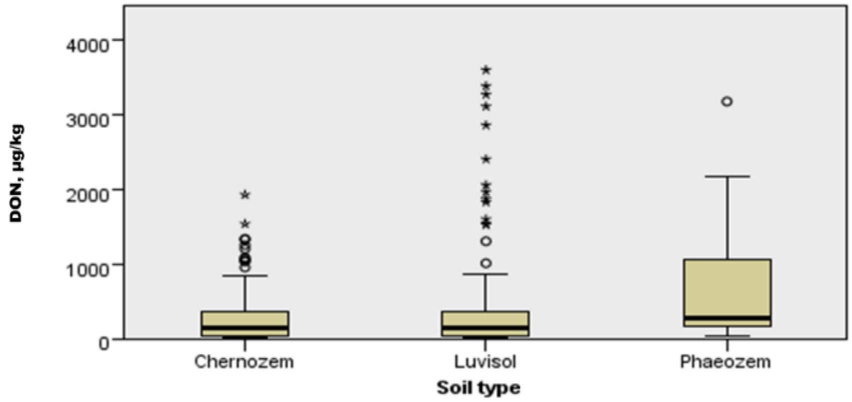

Figure S.3.4. Distribution of deoxynivalenol (DON, µg/kg) by soil type in Romania in the 2012–2014 period with extreme weather events (Kruskal-Wallis non-parametric test for independent samples). ◦◦◦◦◦ * *, ◦ ◦ ** *** * * *** *, ◦ — position of the extreme individual values.

Table S.3.8. Test Statistic Chi-Square for the mean rank of deoxynivalenol (DON) by agricultural year in Romania in the 2012–2014 period with extreme weather events.

Rank	Agricultural Year	N	Mean Rank	Test Statistic	DON, µg/kg
Deoxynivalenol, µg/kg	**2012**	69	108.30	**Chi-Square**	60.515
	**2013**	78	79.72	**df**	2
	**2014**	89	160.39	**Significance Level**	0.000
	**Total**	236			

df —degrees of freedom.

Table S.3.9. Correlations between deoxynivalenol (DON) in the triticale crop and the geographic position of the agricultural region, historical aridity indices (1900–2000), and agrometeorological factors by agricultural year in Romania in the 2012–2014 period with extreme weather events (Pearson correlation coefficient).

Correlations for Romania 43–45 °N, 20–29 °E		Agrometeorological Factors in 2012–2014					Aridity Indices, 1900–2000		Region [1)]
		Average Air Temperature in May	Cumulative Precipitation	Average Precipitation in May	Average of Soil Moisture Reserve	Soil Moisture Reserve in April	de Martonne (Iar-dM)	Climatic Water Deficit (CWD)	
DON, µg/kg	Pearson Correlation	–0.294 **	0.331 **	0.235 **	0.153 *	0.211 **	0.171 **	0.168 **	RO, 43–48 °N 20–29 °E
	Significance (two-tailed)	0.000	0.000	0.000	0.028	0.002	0.007	0.010	
	N	*236*	*236*	*236*	*208*	*208*	*236*	*236*	
DON, µg/kg	Pearson Correlation	–0.296 **	0.225 *	0.204	–0.007	0.086	0.052	0.009	Tr., 45–47 °N 22–24 °E
	Significance (two-tailed)	0.007	0.041	0.065	0.954	0.494	0.641	0.934	
	N	*83*	*83*	*83*	*65*	*65*	*83*	*83*	
DON, µg/kg	Pearson Correlation	–0.220 *	0.333 **	0.229 **	0.149	0.189	0.080	0.083	Tr. + S.H.A., 44–47 °N 22–25 °E
	Significance (two-tailed)	0.012	0.000	0.009	0.135	0.058	0.372	0.352	

N	*128*	*128*	*128*	*102*	*102*	*128*	*128*

Region 1): RO — Romania, Tr. — Transylvania, S.H.A. — Southern Hilly Area. *—correlation is significant at the 0.05 level (two-tailed); **—correlation is significant at the 0.01 level (two-tailed).

Table S.3.10. Correlations between deoxynivalenol (DON) in the triticale crop and precipitation by agricultural year in Romania in the 2012–2014 period with extreme weather events (Pearson correlation coefficient).

Correlations for Romania		Precipitation						
		March	April	May	June	July	August	Cumulative precipitation 1)
DON, 2012	Pearson Correlation	0.074	0.204	0.162	0.259 *	–0.003	–0.169	0.039
	Significance (two-tailed)	0.548	0.092	0.183	0.034	0.980	0.166	0.748
	N	69	69	69	67	68	69	69
DON, 2013	Pearson Correlation	0.428 **	0.258 *	0.104	0.195	–0.380 **	0.170	0.217
	Significance (two-tailed)	0.000	0.023	0.363	0.086	0.001	0.136	0.057
	N	78	78	78	78	78	78	78
DON, 2014	Pearson Correlation	0.206	0.394 **	0.318 **	0.221 *	0.121	–0.090	0.410 **
	Significance (two-tailed)	0.053	0.000	0.002	0.037	0.258	0.400	0.000
	N	89	89	89	89	89	89	89

Cumulative precipitation 1) = cumulative precipitation by agricultural year (from 1st September to 31st August of the subsequent calendar year).

Table S.3.11. Comparison of the effect of agrometeorological factors (Air temperature, cumulative precipitation and soil moisture reserve), agricultural region and agricultural year on deoxynivalenol (DON) level in the triticale crops in Romania in the 2012–2014 period with extreme weather events (Multivariate Tests of Between-Subjects Effects).

Source		Multivariate Tests of Between-Subjects Effects				
		Type III SS	df	MS	*F*	*p*-value
Intercept	Deoxynivalenol, µg/kg	15738010.72	1	15738010.72	43.93	0.000
	Air Temperature, °C	15 705.88	1	15705.88	49272.52	0.000
	Cumulative Precipitation, mm	41365925.36	1	41365925.36	6382.12	0.000
	Soil Moisture Reserve, mc/ha	101878165.60	1	101878165.60	5520.60	0.000
Agricultura l Year	Deoxynivalenol, µg/kg	9750386.25	2	4875193.13	13.61	0.000
	Air Temperature, °C	10.74	2	5.37	16.85	0.000
	Cumulative Precipitation, mm	1150878.56	2	575439.28	88.78	0.000
	Soil Moisture Reserve, mc/ha	166734.16	2	83367.08	4.52	0.012
Agricultura l Region	Deoxynivalenol, µg/kg	7940777.83	6	1323462.97	3.69	0.002
	Air Temperature, °C	115.13	6	19.19	60.20	0.000
	Cumulative Precipitation, mm	705217.82	6	117536.30	18.13	0.000
	Soil Moisture Reserve, mc/ha	1447798.98	6	241299.83	13.08	0.000
Interaction Year x Region	Deoxynivalenol, µg/kg	9289056.23	12	774088.02	2.16	0.015
	Air Temperature, °C	8.99	12	0.75	2.35	0.008
	Cumulative Precipitation, mm	785420.38	12	65451.70	10.10	0.000
	Soil Moisture Reserve, mc/ha	1277880.25	12	106490.02	5.77	0.000
Error	Deoxynivalenol, µg/kg	66999870.80	187	358288.08		
	Air Temperature, °C	59.61	187	0.32		
	Cumulative Precipitation, mm	1212045.92	187	6481.53		
	Soil Moisture Reserve, mc/ha	3450929.70	187	18454.17		
Total	Deoxynivalenol, µg/kg	132326034.17	208			
	Air Temperature, °C	26644.63	208			

**Cumulative Precipitation, mm**	78898778.10	208
**Soil Moisture Reserve, mc/ha**	188557159.35	208

SS—Sum of the Squares; df—Degrees of freedom; MS—Mean Square; *F*—Fisher t*est*.; *p*-value—Significance level.

S.4: Deoxynivalenol occurrence in the cereal crops in Europe on the route of the ''Vb'' cyclones

A). Origin region of the ''Vb'' cyclones

In 2011–2014, **Italy (IT; 43 °N, 12 °E)** reported the highest deoxynivalenol contamination in the durum wheat in 2013 (max. 14452 μg/kg) and maize in 2014 (max. 5336 μg/kg), in the northern country (Figure S.4) [66; 79]. Northern Italy is represented by the Po river basin, which bounds the Ligurian Sea in the west, the Adriatic Sea in the east (the two origin regions of the ''Vb'' cyclones), the Western Alps in the north and the Apennines in the south. The northern region has a humid subtropical climate with strong influences from the surrounding areas, a rich hydrological network formed by the Po and Parma rivers, and the dominant soils are luvisol, cambisol and fluvisol [130-131]. In May–June 2013, northern Italy was under the influence of the southern extremity of the ''Vb'' cyclones— named ''Dominik'', ''Frederik'' and ''Günther'' (Figure 1b) [29-34]. In 2014, the maize grown on the Adriatic coast of the Emilia Romagna region registered a lower deoxynivalenol contamination than in the northwestern region of Parma, although precipitation was the highest. This situation can be explained by the fact that the coastal area has greater historical aridity that counteracted heavy precipitation in July 2014, and the ''Vb'' cyclone— named ''Yvette'' and the ''Vb(1c)'' cyclone— named ''Tamara'' formed in the Gulf of Venice of the Adriatic Sea, that flew Central Europe and Southeastern Europe, respectively [21; 35-36; 38; 47; 66].

B). In Central Europe (50.38 °N, 14.97 °E), the deoxynivalenol occurrence in cereals was reported in countries where Atlantic, Mediterranean, Scandinavian–Baltic and alpine air masses intersect, producing large amounts of precipitation and floods.

Switzerland (CH; 46 °N, 08 °E) reported deoxynivalenol (average 473 ± 99 μg/kg) in wheat grown in the canton of Bern in 2012–2014, with strong interannual variations and minor regional variations. The highest contamination was recorded in 2012 (98%, max. 9880 μg/kg), without correlation with temperature and precipitation (Figure S.4) [77]. Deoxynivalenol contamination was lower in 2013 (56%, max. 5533 μg/kg) and 2014 (52%, max. 5096 μg/kg), even Switzerland was affected by the ''Vb'' cyclones in May–June 2013 and May–July 2014 [29-34; 38; 47]. The factor that favoured deoxynivalenol contamination in wheat was abundant precipitation during the anthesis period. Deoxynivalenol contamination can be correlated with the climate with strong influences of alpine, Atlantic and the ''Vb'' cyclones in 2013 and 2014, the plateau, high hills and plain between the Alps and the Jura Mountains, as well as with dominant phaeozem soils (*Germ*. - Braunerde, brown soils) with pH values 5.0–6.5 and a low aridity index due to the rich hydrological network (Rhine, Rhône, and Aare rivers, and many lakes) (Figure 1) [19; 57; 133]. It is possible that the high deoxynivalenol level in cereals in 2012 (max. 9880 μg/kg) was produced by heavy precipitation on the water-saturated phaeozems in spring as a result of snow melting [24; 25; 77].

In **Germany (DE; 51 °N, 09 °E)**, triticale is grown in the North-Rhine-Westphalia, Lower Saxony, and Bavaria regions, which have a very high humid climate due to the Atlantic and alpine influences, mixed relief— plains, high hills and plateaus of the Alps, the Carpathian Mountains, and the Black Forest Mountains, and dominant soils such as luvisol, phaeozem, chernozem and fluvisol [19; 57; 138]. Germany has a rich hydrological network, which presents a

very high risk of floods (Rhine, Elbe, and Danube Rivers with their affluents and tributaries), and in which fluvisols are strongly represented [53; 120; 121]. No publications on natural deoxynivalenol contamination in cereals were found in 2012–2014, although Germany was affected by extreme precipitation and floods in May–June 2013 and May–July 2014 (Figure 1) [29-35; 47]. However, in 2004 and 2017–2018, Germany reported the highest deoxynivalenol values in artificial infection with *F. culmorum* in triticale varieties (max. 63500 μg/kg in the Modus variety and 25450 μg/kg in the Lombardo variety, respectively), with regional differences determined by annual weather conditions and historical aridity in Europe (Figure S.4) [90-93; 96].

The Pannonian Basin (46.5 °N, 20 °E) recorded very high deoxynivalenol contamination in wheat and maize in 2011–2014, the region being affected by the cold waves caused by a Siberian anticyclone, and the extreme precipitation and floods caused by the ʼʼVbʼʼ cyclones in May–June 2013 and May–July 2014 (Figure 1) [27; 46-47; 69; 74; 78; 80; 82]. The Pannonian Basin has a high humidity because it represents the standard route of the ʼʼVbʼʼ cyclones (43–47 °N, 12–22 °E) and the intersection area of the Atlantic, Mediterranean, alpine and Scandinavian–Baltic air masses along the mountain corridors (Figures 1 and 2) [48; 49; 54]. The Pannonian Basin extends over several countries (the whole of Hungary, eastern Austria, western Slovakia, southern Czech Republic— Moravia, southeastern Poland, southwestern Ukraine, western Romania, northern Serbia— Vojvodina, northeastern Slovenia— Prekmurje, and northeast Croatia— Slavonia), has a characteristic climate— named Pannonian climate, plain and high hills surrounded by mountains (the Alps, the Dinaric Alps, the Carpathian Mountains, and the Balkan Mountains), rich hydrographic network (Danube River; Tisza, Sava, and Morava rivers and their tributaries), and dominant soils are chernozem, phaeozem, luvisol and fluvisol [54; 120-121; 129; 132]. Having similar agroclimatic conditions, the countries of the Pannonian Basin have a similar potential of mycotoxin occurrence in cereals, but with regional differences given by geographic position and local agroclimatic factors.

In **the Czech Republic (CZ; 49 °N, 15 °E)**, the maximum deoxynivalenol contamination in the wheat from the eastern Moravia was 13422 μg/kg in 2011, 10034 μg/kg in 2012 and 10174 μg/kg in 2013, and in the northern Bohemia it was 39900 μg/kg in 2014, and 1510 μg/kg in dry 2015 (Figure S.4) [78; 80]. Another study reported very high deoxynivalenol contamination in the grain and maize sampled from the whole of the Czech Republic for animal feed in 2013–2017 (max. 23350 μg/kg and 29630 μg/kg, respectively) [69]; most likely, maximum values of deoxynivalenol were recorded in 2013 and 2014. The highest deoxynivalenol accumulation in wheat was strongly correlated with maize as the preceding crop in eastern Moravia [69; 80].

Slovakia (SK; 48 °N, 19 °E) reported deoxynivalenol in wheat in 2010 and 2011 (max. 7880 μg/kg and 2120 μg/kg, respectively) and in wheat and oats in 2013 (max. 5100 μg/kg, respectively 490 μg/kg) (Figure S.4) [81-82]. The Czech Republic and Slovakia are small and neighbouring countries with similar agroclimatic conditions.

Hungary (HU; 47 °N, 20 °E) reported deoxynivalenol contamination in wheat in 2013 (average 329 ± 487 μg/kg), and in wheat and maize in 2014 (average of 259 ± 170 μg/kg and 1261 ± 952 μg/kg, respectively) [74].

Poland (PL; 52 °N, 20 °E) reported contamination with deoxynivalenol and other 25 mycotoxins in wheat, triticale, winter barley, and oat because of precipitation in May–June and July 2014. Triticale was the most contaminated grain among all the tested varieties (196–1326 μg/kg, average 573 μg/kg), and there were no statistically significant differences between the individual genotypes (Figure S.4) [64]. The contamination of triticale in southeastern Poland

was similar to that recorded in triticale in northeastern Romania— Moldavia region (151–1533.80 μg/kg, average 562 μg/kg), these regions having similar meteorological conditions in May–July 2014 and similar agroclimatic conditions— arid temperate continental climate. Deoxynivalenol occurrence in cereals in southeastern Poland may be associated with heavy precipitation caused by the northeastern extremity of the ''Vb'' cyclone during the anthesis in 2014, the geographic position of Borusowa in the Małopolska region (Subcarpathian foothills, with strong Scandinavian and Siberian influences and weak oceanic influences) and the Upper Vistula Basin with high flood recurrence, and acid soils (podzol, fluvisol, luvisol) (Figure 1) [38; 47; 53-54; 57; 64; 128]. Other factors that could have favoured grain contamination with *Fusarium* spp. and deoxynivalenol occurrence in Borusowa were the extreme rainfall (150 mm) in the Tatra Mountains in May 2014, increased flow of the Vistula River and floods of the river meadows with acid soils [38; 120-125].

C). Eastern European countries have similar agroclimatic conditions, being located in the Eastern European Plain or the Russian Plain that stretches from the Carpathian Mountains to the Ural Mountains [57; 108].

Lithuania (LT; 55 °N, 24 °E) reported high deoxynivalenol contamination in wheat and their products in 2012 (max. 8845.1 μg/kg) when it recorded a Siberian anticyclone situation with frost and heavy snowfall in January–February, followed by excessive soil moisture in spring and moderate summer temperatures (Figures 1 and S.4) [24; 83]. The damaging effect of humidity during the critical periods of vegetation was determined in the case of spring triticale in 2017 when harvesting was delayed, and the deoxynivalenol level increased 3.5 times [110].

In **European Russia (Eu-RU; 55 °N, 40 °E)**, triticale is grown in the Central District— the western part and the Volga District— the southeastern part, along with wheat, rye and maize [139-140]. Until 2010, scientific publications had reported a low incidence and level of deoxynivalenol in wheat in the Russian Federation because of the unfavourable agroclimatic conditions for Fusarium Head Blight (FHB) disease: frequent epidemic— in the North Caucasus and the Far East; sporadic disease— in the central and northwestern parts of Russia, at the Urals, in the Baltic countries (Lithuania, Latvia, and Estonia), in southwestern Ukraine, and in the Republic of Belarus; and the lack of the disease— in Asia of Middle and Kazakhstan [63; 113]. In recent years, as a result of the global warming process, wheat contamination was observed in Western Siberia and Northern Kazakhstan, which have an arid subarctic climate that does not favour FHB disease [61; 112; 141]. A global mycotoxin survey in 2008–2017 showed that Eastern Europe (European Russia, Ukraine, and Belarus) had the highest level of deoxynivalenol 600 μg/kg in animal feed in 2013 [84]. Also, animal feeds in Russia had high contamination with mycotoxins in the heavy rainy 2014 (max. DON 5000 μg/kg, FUM 5000 μg/kg, AF 80 μg/kg, T-2 toxin 1300 μg/kg, ZEN 170 μg/kg, and OTA 16 μg/kg) (Figure S.4) [86]. In 2017, analysis of wheat from the Volga, the Ural and the Western Siberian regions showed contamination with *Fusarium* spp. and deoxynivalenol, with significant regional differences; the highest contamination was recorded in southwestern Siberia (96.1% *Fusarium* spp. in Novosibirsk Oblast and Altai Krai; 0–2228 and 2239 μg/kg deoxynivalenol in Krasnoyarsk Krai [112]. European Russia is an agricultural area of the Russian Federation; it is characterized by a humid continental climate (mean annual temperature 6–10 °C, precipitation 500–1000 mm), with chernozem, phaeozem, fluvisol and luvisol (pH >8.5–4.5, from the south to the north), a rich hydrological network— Volga, Irtysh and Ob rivers, and numerous lakes, and a mixed relief— the East European Plain and the West Siberian Plain, plateaus and hills of the Caucasus Mountains and the Ural Mountains [57; 139-140; 142]. In 2012–2014, European Russia was entirely under the influence of a Siberian anticyclone in

January–February 2012, at the northeastern extremity of the ''Vb'' cyclone in June 2013, and at the eastern extremity of the ''Vb'' and ''Vb(1c)'' cyclones in May–July 2014 (Figure 1) [27; 46-47]. The increased deoxynivalenol occurrence in animal feed in the Russian Federation in 2013 and 2014 may be associated with weather events in the western-southwestern European Russia— the Northwest, Central and North Caucasus Federal Districts, which determined the soil leaching process and favoured cereal contamination with mycotoxins over the maximum allowed limits (Figures 1 and S.4) [84; 86; 103; 122-123; 125].

D). In **Southeastern Europe (42 °N, 22 °E)**, a massive deoxynivalenol contamination in cereals was recorded in Croatia, Bosnia and Herzegovina, and Serbia which were affected by historical precipitation and floods caused by the ''Vb(1c)'' cyclone in May–July 2014 (Figure 1).

Bosnia and Herzegovina (BA; 44 °N, 18 °E) and **Croatia (HR; 45 °N, 15 °E)** are located on the northeastern coast of the Adriatic Sea, the origin place of the ''Vb(1c)'' cyclone in 2014. In Bosnia and Herzegovina, the maximum deoxynivalenol contamination was 2123 μg/kg in wheat, 578 μg/kg in barley and 8529 μg/kg in maize in 2013–2015, the highest deoxynivalenol contamination 1611 ± 1825 μg/kg being recorded in the extremely rainy 2014 (Figure S.4) [75]. In Croatia, deoxynivalenol contamination was 1461 ± 2265 μg/kg in unprocessed cereals and 2687 ± 2731 μg/kg in soybean in 2014–2015 (Figure S.4) [76]. Cereals are grown in the north and northeast of the two countries, in the lowlands— with podzo-luvisol, fluvisol, gleysol and hilly zones— with luvisol [57]. In 2014, Croatia and Bosnia and Herzegovina recorded their highest precipitation and floods in 120 years [38]. Deoxynivalenol contamination in cereals in Croatia and Bosnia and Herzegovina was determined by extreme precipitation and historical floods on the Bosna and Sava rivers in 2014 and by the high to extreme humidity in cereals growth and harvesting in 2015 [38,47,75–76].

The ''Vb(1c)'' cyclone produced heavy precipitation in **Albania (AL; 41 °N, 20 °E)** and **Greece (39 °N, 22 °E)**, but high historical aridity had not favoured or inhibited deoxynivalenol occurrence in cereals [65,143].

The highest contamination was recorded in **Serbia (RS; 44 °N, 21 °E)** in the extremely rainy 2014, when the deoxynivalenol incidence in cereals was very high (96% positive and 45.6% over the maximum limit), and the maximum contamination was 1440 μg/kg in wheat and 9050 μg/kg in maize (Figure S.4) [67–68,70]. Maize was the most contaminated in the northern Serbia— Vojvodina (428–16350 μg/kg, average 3522 ± 2668 μg/kg) (Figure S.6) [67]. Massive deoxynivalenol contamination was due to extreme precipitation and historical floods caused by increased river flow in western, southwestern, central and eastern Serbia (Drina, Lim, Kolubara, Zapadna Morava, Velika Morava, Mlava, Ibar, Timok and Sava rivers, and the Danube River), produced by the ''Vb'' and ''Vb(1c)'' cyclones in May–July 2014 [35–36,38,47,67–68,70,109].

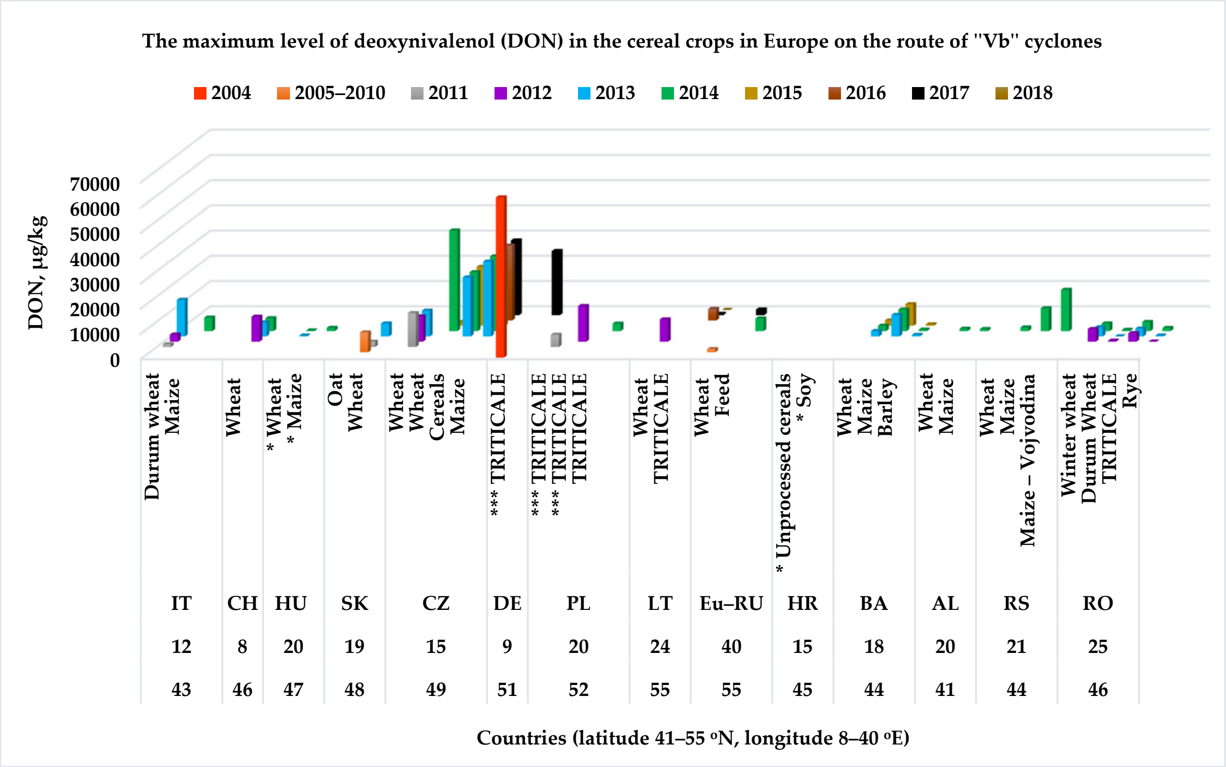

Figure S.4. Spatial and geographic distribution of the maximum level of deoxynivalenol (DON) in the cereal crops in Europe on the route of the "Vb" cyclones. * Average values of deoxynivalenol in cereals; *** TRITICALE—artificial infection with *Fusarium culmorum*. Southern Germany, the Czech Republic, western Slovakia and southwestern Poland recorde the highest frequency of heavy precipitation caused by the "Vb" cyclones (7 out of 10 precipitation events) [19].
